# An accurate alignment-free protein sequence comparator based on physicochemical properties of amino acids

**DOI:** 10.1038/s41598-022-15266-8

**Published:** 2022-07-01

**Authors:** Saeedeh Akbari Rokn Abadi, Azam Sadat Abdosalehi, Faezeh Pouyamehr, Somayyeh Koohi

**Affiliations:** Department of Computer Engineering, Sharif University of Technology, Tehran, Iran

**Keywords:** Computational biology and bioinformatics, Classification and taxonomy, Protein analysis

## Abstract

Bio-sequence comparators are one of the most basic and significant methods for assessing biological data, and so, due to the importance of proteins, protein sequence comparators are particularly crucial. On the other hand, the complexity of the problem, the growing number of extracted protein sequences, and the growth of studies and data analysis applications addressing protein sequences have necessitated the development of a rapid and accurate approach to account for the complexities in this field. As a result, we propose a protein sequence comparison approach, called PCV, which improves comparison accuracy by producing vectors that encode sequence data as well as physicochemical properties of the amino acids. At the same time, by partitioning the long protein sequences into fix-length blocks and providing encoding vector for each block, this method allows for parallel and fast implementation. To evaluate the performance of PCV, like other alignment-free methods, we used 12 benchmark datasets including classes with homologous sequences which may require a simple preprocessing search tool to select the homologous data. And then, we compared the protein sequence comparison outcomes to those of alternative alignment-based and alignment-free methods, using various evaluation criteria. These results indicate that our method provides significant improvement in sequence classification accuracy, compared to the alternative alignment-free methods and has an average correlation of about 94% with the ClustalW method as our reference method, while considerably reduces the processing time.

## Introduction

One of the most important disciplines in bioinformatics is protein classification, which is used to discover phylogenetic and evolutionary relationships amongst species^1,2^. Furthermore, accurate classification of a protein sequence among large protein sequence database is critical while developing pharmaceutical products^3^, such as vaccines, which is becoming increasingly important, particularly in the wake of the coronavirus epidemic. Without current redundancy, the protein sequence database contains over 190 million inputs, and the number of unique functional domains is much larger^4^. Any attempt to annotate protein function has many issues due to the huge amount of data connected with these proteins. In this manner, protein classification into sequence and structural classes has long been used as a means of simplifying the challenge. As a result, a variety of approaches are used, as explored below.

The process of obtaining a large number of protein sequences has been made easier for researchers because of advances in sequencing technologies^5–7^. As a result, comparing and phylogenetic analysis of these biological sequences becomes a new issue, posing challenges in a variety of areas, including processing time and resource management, due to the large number of data to be considered^5^. Various methods have been proposed up to this point, which can be divided into two categories: alignment-based methods and alignment-free methods^8^. For a clear description, Table 1 provides a list of online tools developed for some of the alignment-based and alignment-free methods, as well as the summary of their corresponding comparison algorithm. Alignment-based methods relying on multiple sequence alignment, which commonly uses some sort of evolutionary tools including sequence similarity search tools (e.g., BLAST^9^, FASTA^10^), multiple sequence aligners (e.g., ClustalW^11^, MUSCLE^12^, MAFFT^13^), sequences’ profile search programs (e.g., PSI-BLAST^14^, HMMER/Pfam^15^), and whole-genome aligners (e.g., progressive Mauve, BLASTZ^16^, TBA^17^). Although these methods achieve up-to-scratch results in evolutionary relationships discovery, they are generally time and resource consuming and rely on multiple assumptions about the evolution of the sequences to be compared (i.e. various parameters should be set, such as substitution matrices, gap penalties, and threshold values for statistical parameters which are somewhat arbitrary)^2^. As a consequence, several alignment-free methods have been presented to prevail over these drawbacks. Alignment-free approaches include any method for evaluating sequence similarity/dissimilarity that does not apply or produce sequence alignment at any step of the algorithm; instead, they use feature extraction to extract the required information from the query sequences^2,5^. As mentioned before, the alignment-based methods provide high accuracy at the cost of being time-consuming and expensive in memory usage. In contrast, alignment-free methods are fast in computational speed and have been introduced to overcome the complication of sequence alignment^2,5^. However, they confront the obstacle of accurate comparison and classification. In other words, one of the outstanding concerns in this field is to develop an accurate alignment-free approach that can be utilized in practice, and hence, studies in the field of developing alignment-free methods are mostly focused on this.

**Table 1:** A list of online tools developed for some of the alignment-based and alignment-free methods, as well as the summary of their comparison algorithm.

Category	Tool	Algorithm
Alignment-based	ClustalW^11^	It employs progressive alignment techniques, which begin with the most similar sequences and work their way down to the least similar sequences until a global alignment is achieved
	Muscle^12^	It creates a progressive alignment, followed by a horizontal refinement
	Clustal Omega^18^	This multiple sequence alignment tool uses seeded guide trees and HMM profile-profile approaches to produce alignments, and is appropriate for medium-to-large alignments
	T-Coffee^19^	It is a multiple sequence alignment technique based on the consistency model that tries to avoid the drawbacks of progressive alignment approaches, and it is appropriate for small alignments
Alignment-free	FFP^20^	It is a whole genome/proteome comparison tool using Feature Frequency Profile-based measurements
	CVTree^21,22^	It utilizes word composition to build phylogenies from the whole genome sequences
	NASC^23^	It is a set of six alignment-free approaches, including 4 word-based measures (e.g. Mahalonobis distance), and 2 Information Theory-based measures (e.g. Kolmogorov complexity)
	kmacs^24^	It is a alignment-free sequence comparison tool which uses the k-mismatch average common substring method
	Squared Euclidean distance^34^	It is a combination of word-based encoding and squared Euclidean distance

Feature extraction from a protein sequence is the challenging part of protein classification studies in any of the approaches discussed above; as a result, various alignment-free methods have been developed in this area. These techniques can extract features in one of two ways^25^: (1) the protein's amino acid composition, which includes the frequencies of the 20 distinct amino acids within the sequence as well as their physicochemical qualities, or (2) the order and positional information of amino acids within the sequence. So, the physicochemical properties of amino acids can be used to derive features from a protein sequence. As is obvious, a protein sequence is made up of amino acids, each of which has its own set of physicochemical properties that influence protein structures, functions, folding, protein–protein interactions, and evolutionary patterns^26,27^. As a result, amino acid physicochemical properties play an essential role in protein sequence similarity analysis, protein subcellular localization prediction, and protein structural class prediction. Another aspect to consider is that relying solely on the amino acids’ physical qualities results in the loss of various information, such as the number of amino acids, their location in a string, and so forth^25^. This positional information, on the other hand, can significantly affect the accuracy of similarity analysis between two sequences. As a result, by relying simply on either physicochemical characteristic or positional information, some data is lost, and the information embedded within a protein sequence is not fully utilized.

In this regard, many studies have been conducted in this area, each of which has used one of these two methodologies or a combination of them. One of these state-of-the-art methods, DCGR^27^, for example, is built upon the chaos game model based on the physicochemical features of amino acids. The Energy matrix approach^2^ is another study that is based on physicochemical properties and the position-feature Energy Matrix. Apart from these two studies, methods^8,25,28^, and many others have utilized the physicochemical features in various ways in their methods. The main drawback of these studies is that they just use the value of physicochemical properties of amino acids in an indirect manner, which results in some information loss. Moreover, there are other concerns, such as the high complexity and increasing volume of calculations in these methods, such as DCGR.

On the other hand, other studies, such as natural vector based method^29^ that is based on the k-mer natural vector, fuzzy integral (FI) based method^5^ that is based on the fuzzy integral and Markov chain, and^7^ method, and many more, do not consider physicochemical properties of the amino acids. Even though these properties provide essential factors for predicting the function and structure of protein sequences.

As a consequence, based on the previous studies, it appears that combining physicochemical properties of amino acids with other properties of protein sequences can assist to enrich the features derived from the sequences^2,27^. After retrieving the attributes of the protein sequences, numerous methods have been proposed to leverage these features to complete the target tasks and perform the desired calculations. These approaches either require large memories or are not optimal in terms of algorithm execution time, although they achieve acceptable accuracy among various alignment-free methods^2,5,27^. To build an efficient algorithm for protein classification, several tradeoffs between memory requirement, time consumption, and high accuracy, must be evaluated, which necessitates effective feature extraction.

Given aforementioned challenges of the available alignment-free methods, including clustering accuracy, accuracy of the resultant similarity/dissimilarity scores in comparison to the alignment-based methods, computation speed, considering each protein sequence as a single unit to be processed, and thus, ignoring individual indels' effect, and finally, not involving the physicochemical properties of the amino acids, we propose a simple but efficient vector-based method named PCV (PhysicoChemical properties Vector) to numerically characterize a protein sequence, utilizing the value of the amino acids' physicochemical properties, as well as the positional information of the letters. In more details, we utilize all physicochemical properties of the amino acids, we split each protein sequence into fixed-size blocks, and encode each block of protein applying the proposed encoding method. As a result, PCV takes into account both the influential amino acids' physicochemical properties and the local sequence comparisons, none of which have been considered by most of the existing alignment-free methods. In this work, we also attempt to incorporate the key advantages of the alignment-free methods, including increased processing speed and reduced resource utilization, in comparison to the alternative alignment-based and alignment-free methods.

The steps of our approach are as follows:

1. Extract physicochemical properties from the AAindex.
2. Cluster properties into 110 items.
3. Split a sequence into fixed-length blocks.
4. Calculate statistical or positional characteristics and produce vectors based on the physicochemical properties.
5. Calculate the distance metric between different spices vectors to perform the evolutionary analysis.

Going through above steps, PCV offers several key advantages: (1) simple feature extraction by using the value of the amino acids’ physicochemical properties, and (2) preserving more sequence information by using the amino acids’ physicochemical properties and moment values as a combination of protein composition and positional information, (3) incorporating locational information of amino acids to fully utilize all information embedded within a protein sequence, (4) providing computational parallelism as the result of sequence partitioning into fixed-length blocks, which facilitates parallel operation on various blocks through iterative steps, (5) capable of handling numerous mutations in compared sequence, (6) reduced runtime, compared to alternative methods.

As a comparative study, we evaluate PCV on a variety of datasets with diverse sequence lengths and numbers of sequences. Also several comparison metrics (e.g. Correlation Coefficient and Robinson Foulds distance) are applied to compare our results with those of alternative methods, such as fuzzy integral based method^5^ and ClustalW as the reference method^11^. The results confirm that PCV offers higher accuracy, provides correct evolutionary relationships of different kinds of species, and offers high speed comparison. The rest of the paper is organized as follows. Details of the proposed method, i.e. PCV, are introduced in “Method and materials” section. The experimental setup, simulation results, and comparative studies are explored in “Results” section. Finally, the paper is concluded in “Discussion” and “Conclusions” sections.

## Method and materials

**Figure 1:**
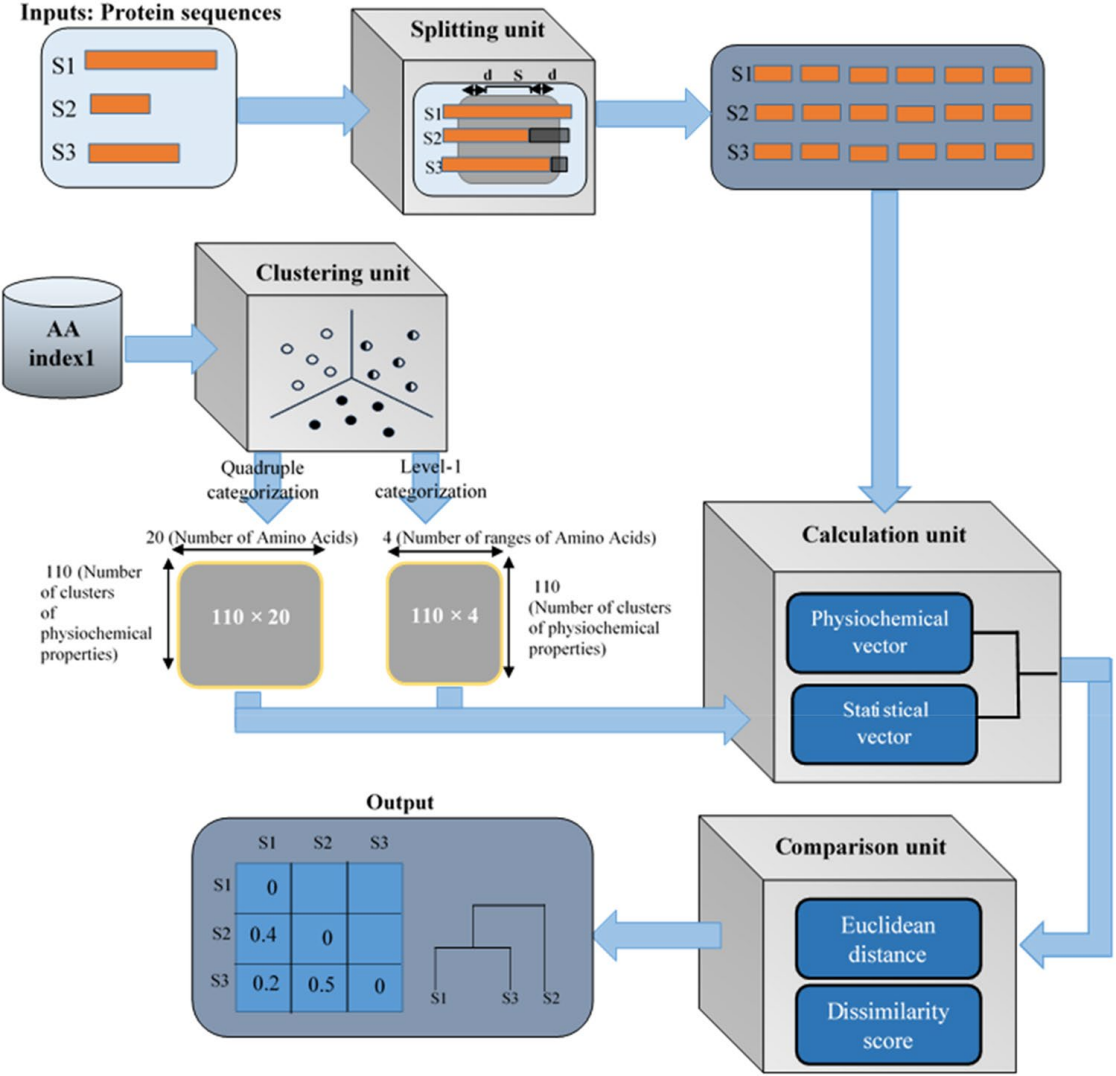
The overall schematic of PVC algorithm with its four units: (**a**) clustering unit, (**b**) splitting unit, (**c**) calculation unit, and (**d**) comparison unit.

As the main target of this paper, we attempt to develop an accurate alignment-free approach to boost the speed of protein comparison, while preserving resource efficiency. In this case, we utilize two different types of data: (1) physical and chemical characteristics of amino acids, and (2) statistical information of the amino acids, such as their spatial frequency within the sequences. For this purpose, as shown in Fig. 1, we present four units to utilize and process this information in our proposed method, called as PCV:

1. Clustering unit categorizes physicochemical features of the amino acids. This unit categorizes 566 amino acid features into 110 classes. As a result, although all features are utilized, the amount of data fed to the comparison algorithm is reduced.
2. Splitting unit splits protein sequences into fixed-size blocks: Splitting sequences preserves local information, while enables indel modeling.
3. Calculation unit generates statistical information vector for each block: In this unit, statistical information of the amino acids and their order are calculated based on the corresponding physicochemical features.
4. Comparison unit computes dissimilarity/similarity metric. This unit computes Euclidean distance of physicochemical and statistical information vectors for each pair of blocks of two sequences, and so, utilizes the extracted distance to compute the dissimilarity metric between input sequences.

The process of PCV algorithm is such that first of all, its Splitting unit elongates the input sequences to a specific length and splits them into pieces of fixed length. Afterwards, the Calculation unit encodes these components using the physicochemical properties clustered by the Clustering unit. Finally, the PCV algorithm calculates a score as the degree of similarity / dissimilarity between each two sequences. To clarify these functionalities, Fig. 1 shows the relationship between various units of the PCV algorithm. In the following, we provide a detailed description of each unit of the PCV algorithm.

### PCV’s units

#### Clustering unit

The physicochemical properties of amino acids demonstrate the characteristics of biochemical reactions and have been widely used in bioinformatics research. AAindex is a database of numerical indices representing diverse physicochemical and biochemical properties of amino acids^26^. This database consists of three sections: (1) AAindex1 for the amino acid indices, as we require in this paper, (2) AAindex2 for the amino acid substitution matrices, and (3) AAindex3 for the amino acid contact potentials.

AAindex1 includes 566 properties for amino acids^26^. Although it can be used in various applications, such as sequence comparison involving high number of sequences, this massive volume of data arises resource consumption and computational complexity concerns. In this manner, we propose grouping them together to overcome these challenges. For this purpose, we first calculate the correlation between each pair of 566 properties to obtain a 566 × 566 correlation matrix. Then, we use this matrix to calculate a pairwise Euclidean distance in a 566-dimensional space to achieve a 1-d array. This array is then used to feed the “linkage method” (this is also known as the Farthest Point Algorithm or Voor Hees Algorithm)^30,31^, as is discussed in more details in the supplementary materials section "AAindex1 clustering". Finally, we form flat clusters from the hierarchical clustering defined by the given linkage matrix, provided that each flat cluster has a cophenetic distance not greater than 0.2 × max_distance (maximum Euclidean distance from the 1-d array). The details of clustering these 566 properties into 110 groups can be seen in Table S1 of the supplementary materials.

**Figure 2:**
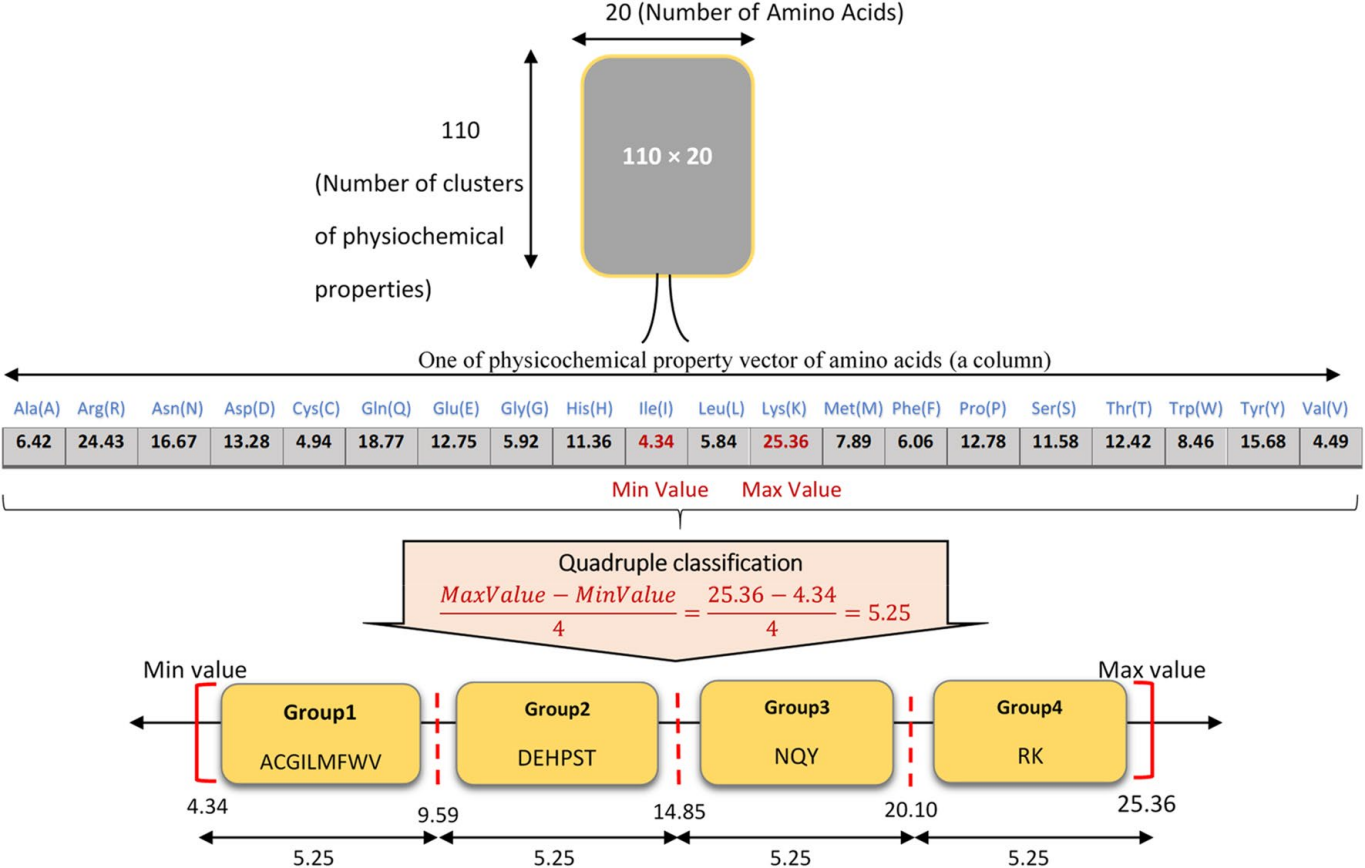
An example of categorizing a sample class into four groups of amino acids.

Once the physicochemical properties are clustered, to use each class, a representative value of all the attributes in that class should be determined. In this paper, we employ the average values of all attributes of each class for each amino acid, resulting in a 110 × 20 matrix, which replaces the 566 × 20 matrix used by AAindex1 to feed the PCV. In addition, these values are normalized by Studentized residual to unify the impact of the physicochemical properties. This clustered 110 × 20 matrix is called “level-1 categorization”, which is still a significant amount of data to be used through the sequence comparison process. To resolve the issue, we also partition the numerical range of each of these 110 classes into four equal ranges, and allocate each amino acid to one of the four ranges at which its corresponding numerical value is located. This categorization scheme, called as quadruple classification, is utilized in other parts of the PCV as well. It is worth noting that the number of amino acids in each of the four categories might be different. Figure 2 depicts an example of this categorization scheme for one sample class of the 110 classes.

#### Splitting unit

Splitting unit partitions protein sequences into blocks of constant length. This partitioning preserves the locality information of protein sequences through the comparison process, which is also empowered by the physicochemical characteristics of amino acids. However, due to the different lengths of input sequences, first of all, lengths of all sequences are made equal to the length of the longest sequence by appending a meaningless substring to their tails.

**Figure 3:**
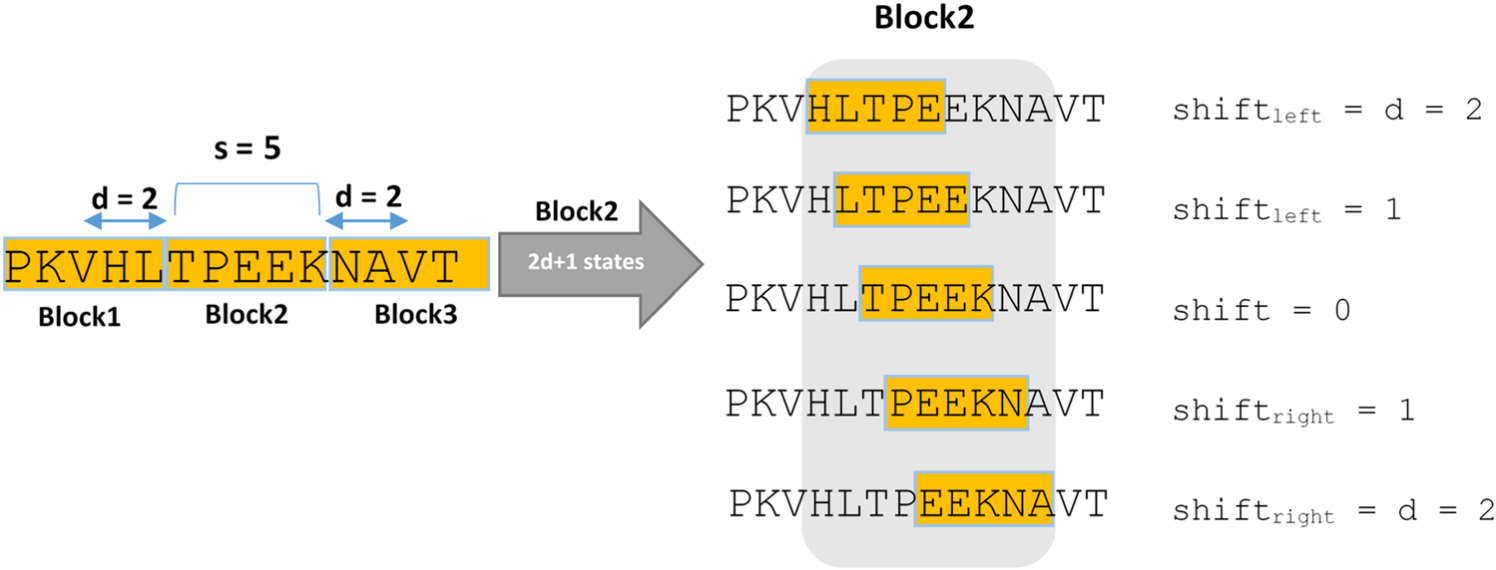
An example of sequence splitting into fixed size (S) blocks by various right and left shifts (d as maximum shift); in this example S = 5 and d = 2.

The occurrence of indels within the sequences and their diagnosis raises some challenges since sequence partitioning into fixed length blocks might prevent their appearances. To resolve this issue, as shown in Fig. 3, we adopt a sliding window (with fixed size S) with maximum shift of d characters to the right and the left of a fixed point, rather than selecting each block from a fixed location. In other words, each initial block is left and right shifted by equal or less than d characters, and so, results in a total of 2d + 1 states, all of them are fed to the PCV, whose best result, in terms of the distance between the blocks, is reported. It should be noted that block size and d value should be pre-determined, as presented in section "Block size and shift analysis" of the supplemental materials. Analyzing various datasets, we chose block size of 50 with maximum shift value d equal to 5, as the suitable values of parameters.

#### Calculation unit

Once the sequences have been split into blocks, they must be encoded and compared with each other. For this purpose, the Calculation unit performs the encoding and the comparison unit compares the sequences. Each block is encoded as a vector, consisting of two parts: (1) physicochemical vector, representing values of the block's physicochemical attributes collected and preprocessed from AAindex1, and (2) statistical vector, representing statistical information of the block's amino acid groups. In the following, we describe these two parts of the vector in more details.

##### Physicochemical vector

**Figure 4:**
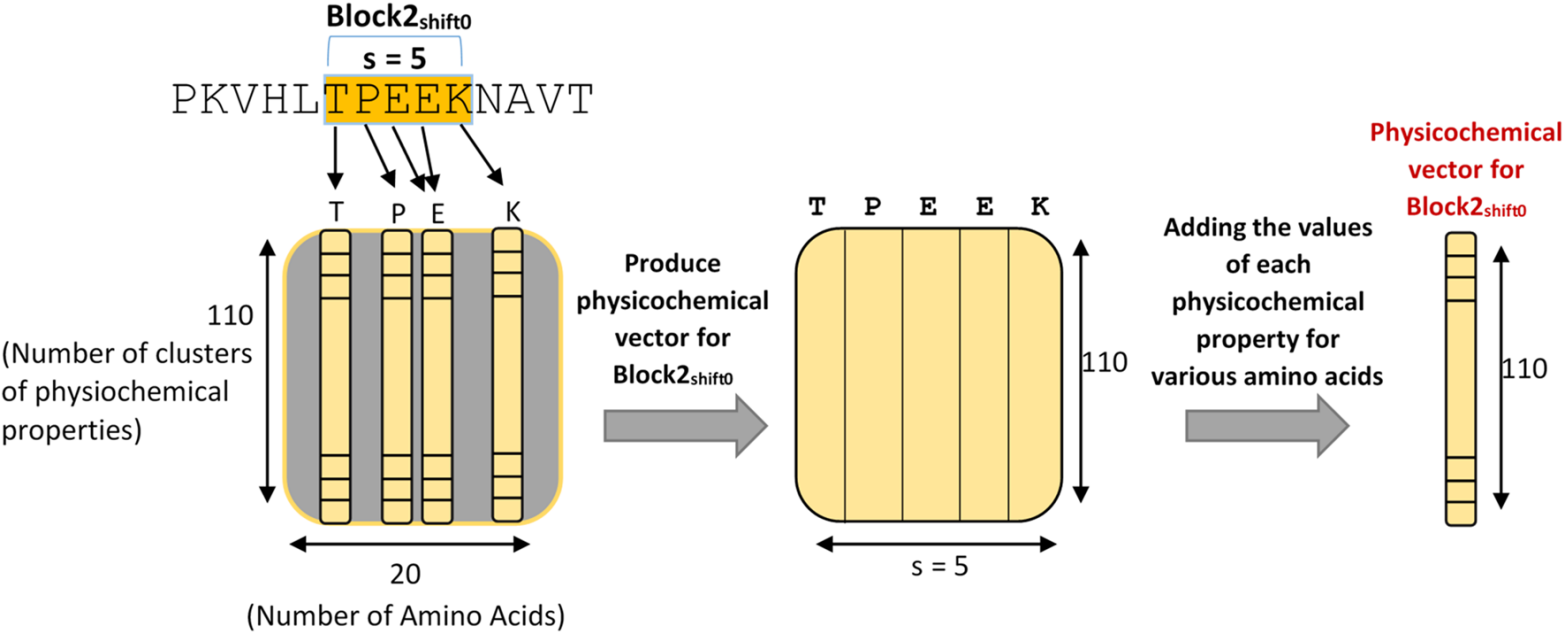
Producing physicochemical vector for each block.

As described in the clustering unit, the 566 properties provided for each amino acid in AAindex1 are clustered into 110 classes. As a result, for each amino acid in the split block, the calculating unit allocates a vector of length 110 to represent its physicochemical properties. In this manner, for a block of length |S|, a matrix of size 110 ×|S| is generated. As the next step for each block, the values of each physicochemical property for various amino acids are added up. It should be noted that this process is performed for all possible shifts of each block. Figure 4 depicts the aforementioned step-by-step procedure.

##### Statistical vector

**Figure 5:**
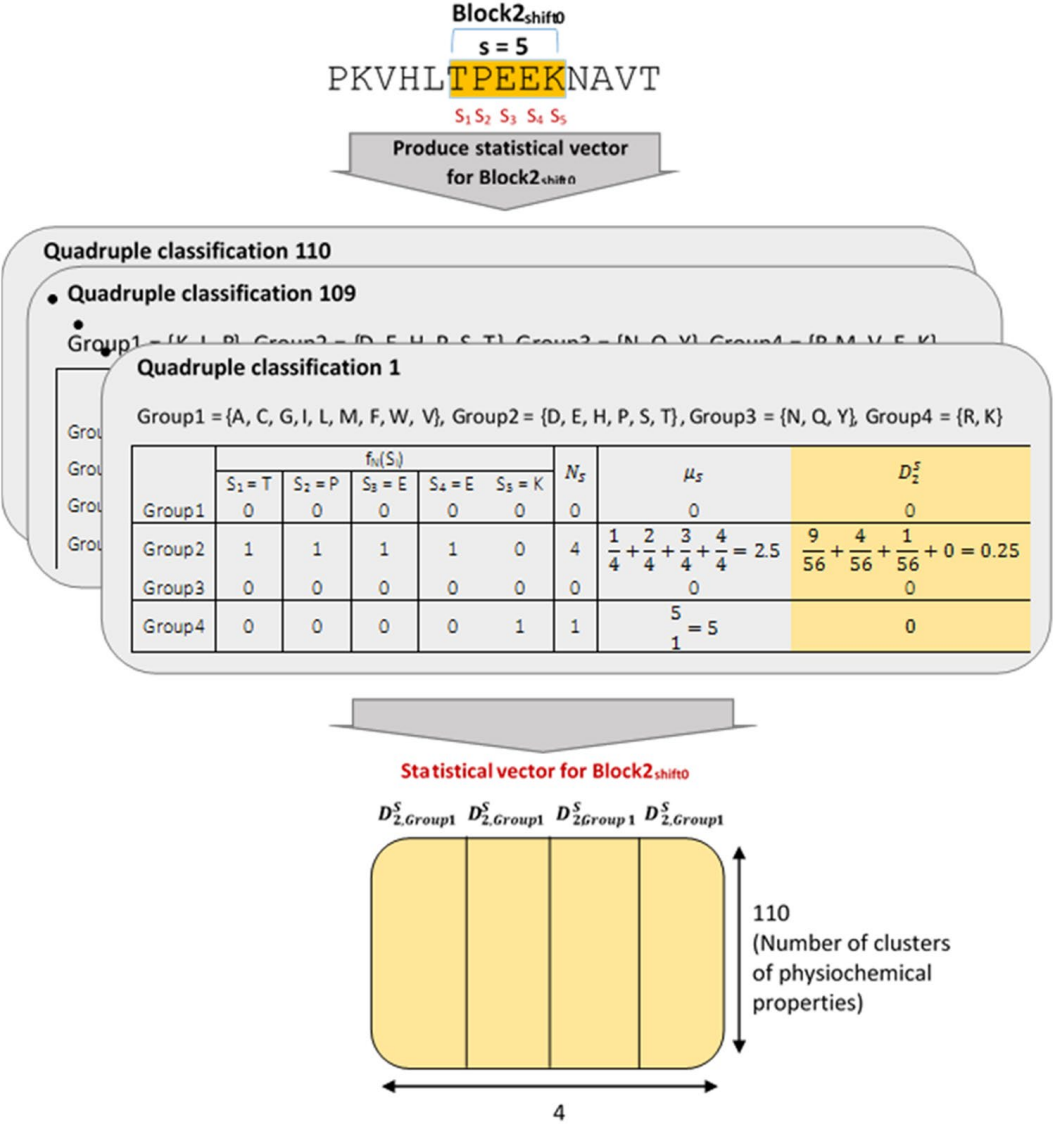
Producing statistical vector for each block.

As described in the introduction, in order to extract the most information from a protein strand, the statistical and spatial information of amino acids must be analyzed, alongside their physicochemical properties. Specifically, quadruple classification as defined in the clustering unit is employed to produce statistical vectors for each block. For this purpose, 2nd moment of position of each group of quadruple classification is used for each block as statistical vector which is derived from the other two concepts as follow: (1) the number of repetitions of each quadruple classification group for each property in the block, and (2) the mean position of each group for each property in the block. This statistical information can be calculated by Eqs. (1) to (3), respectively.

1 $$N_{S} = \mathop \sum \limits_{i = 1}^{\left| S \right|} f_{N} \left( {S_{i} } \right)$$

2 $$\mu_{S} = \mathop \sum \limits_{i = 1}^{\left| S \right|} i \cdot \frac{{f_{N} \left( {S_{i} } \right)}}{{N_{s} }}$$

3 $$D_{2}^{S} = \mathop \sum \limits_{i = 1}^{\left| S \right|} \frac{{\left( {i - \mu_{S} } \right)^{2} f_{N} \left( {S_{i} } \right)}}{{N_{S} N}}$$

where S represents a block of protein sequence, N_s_ is the number of repetitions of each quadruple classification group for each property in block S, f_N_ is a binary function calculated for each group of quadruple classification of each of 110 property classes and assumes that all members (amino acids) of each group are similar. Therefore, for each group of quadruple classification, f_N_ equals one if its input character (each amino acid of block S) belongs to the intended group, and its zero otherwise, μ_S_ is the mean position of each group of quadruple classification for each property in block S, $D_{2}^{S}$ is the 2nd moment of position of each group of quadruple classification for each property in block S. Finally, the calculated statistical vector is normalized using the Studentized residual method. Therefore, the statistical vector is a 4 × 110 vector, in which each of the four rows represents the second moment of the corresponding group of quadruple classification and 110 columns represent 110 property classes of amino acids. For more clarity, Fig. 5 depicts the generation process of a statistical vector for a sample block in a more practical manner.

#### Comparison unit

**Figure 6:**
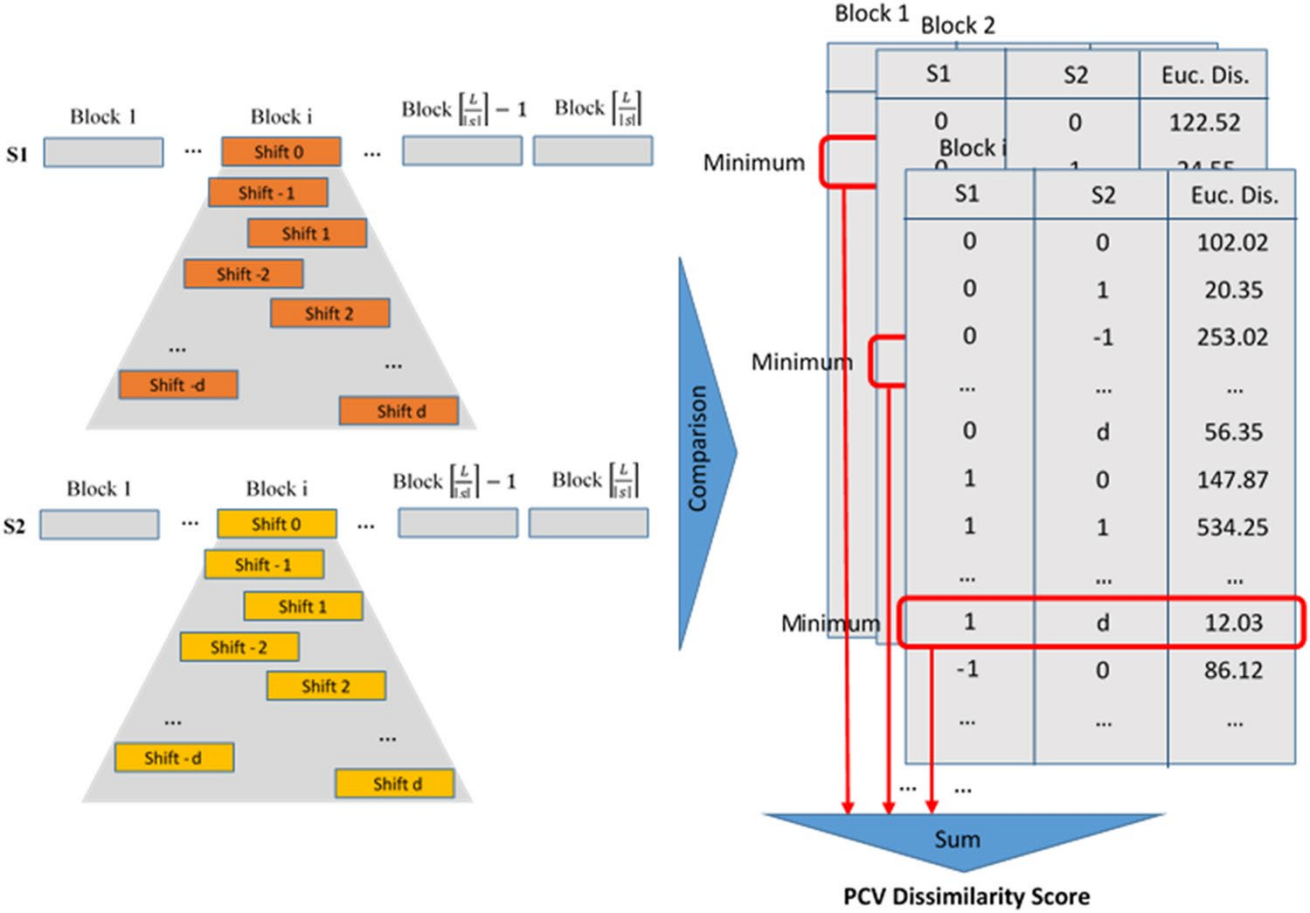
The method of comparing blocks in the PCV algorithm's comparison unit.

The vectors produced by the computation unit for each block, as well as their corresponding shifts, are compared in this unit. To accomplish so, it compares ith blocks of two sequences using the Euclidean distance, as addressed in Eq. (4).

4 $$\begin{aligned} ED_{block\,i, shift\,j\,and\,k} & = \sqrt {\mathop \sum \limits_{m = 1}^{\left| S \right|} \left( {v_{2,i,j,m} - v_{1,i,k,m} } \right)^{2} } , - d \le j, k \le d \\ V_{sequence\,1,block\,i,shift\,j} & = \left( {v_{1,i,j,1} , v_{1,i,j,2} ,v_{1,i,j,3} , \ldots , v_{1,i,j,550} } \right), \\ V_{sequence\,2,block\,i,shift\,k} & = \left( {v_{2,i,k,1} , v_{2,i,k,2} ,v_{2,i,k,3} , \ldots , v_{1,i,k,550} } \right) \\ \end{aligned}$$

where V refers to the encoded vectors of the split blocks, as generated by appending physicochemical and statistical vectors, and d denotes the maximum shift value for each block. Shifts are accomplished as shown in Fig. 6, while all resultant shifts of the two blocks are compared with each other. The minimal distance between two blocks is presented by the minimum distance between any pair of their shifted variants. This value is utilized for computing dissimilarity score of PCV. Finally, similar to Eq. (5), the dissimilarity score between the two sequences is calculated by sum of the Euclidean distance values picked from all pairs of their shifted variants.

5 $$D_{Seq1,Seq2} = \mathop \sum \limits_{i = 1}^{{\frac{{\left| {Seq} \right|}}{\left| S \right|}}} {\text{min}}\left( {ED_{block\,i,\,shift\,j\,and\,k} } \right), - d \le j, k \le d$$

### Datasets

The proposed algorithm is tested on 12 different datasets, available at The National Center for Biotechnology Information (NCBI), F10 and G11 protein datasets, and other public databases^2,5,7,29^. Our datasets include 9 ND5, 8 ND6, 24 TFs, Coronavirus in two versions, i.e. a) 24 sequences and b) 50 sequences, Betaglobin in three versions, i.e. a) 9 sequences, b) 50 sequences, and c) 88 sequences, 27 AFPs, 114 HRV,1163 influenza, and 20 xylanases protein sequences. The detailed information of each dataset is provided in Table 2. According to this table, these datasets are very diverse in terms of number of sequences and sequence lengths, ranging from about 150 to about 2200 in length, and from 8 to more than 1150 sequences. Finally, it should be noted that these protein sequences are used as Fasta files. Access information of this data can be found in the "Data" section of the supplementary materials.

**Table 2:** Benchmark datasets details.

Datasets	Number of sequences	Amino acids (approximate lengths)	Sources
Betaglobin	9	150	NCBI
Betaglobin	50	150	EM Article^2^ and fuzzy integral^5^
Betaglobin	88	150	Natural vector Article^29^
ND5	9	600	NCBI
ND6	8	175	NCBI
Coronavirus	24	1500	NCBI
Coronavirus	50	1500	EM Article^2^ and fuzzy integral^5^
TF	24	700	NCBI
AFP	27	140	EM Article^2^
HRV	114	2200	Natural vector Article^29^
Xylanases	20	500	Fuzzy integral Article^5^
Influenza A	1163	480	Natural vector Article^29^

## Results

As mentioned in the data section, we utilized 12 datasets (as listed in Table 2) with substantial variation, in terms of the number of sequences and sequence lengths used in earlier studies, to evaluate PCV in terms of correctness and speed. PCV's applicability and accuracy in comparing sequences can be assessed in a variety of ways, including qualitative phylogenetic tree analysis, RF (Robinson–Foulds) distance measurement, CC (Correlation Coefficient) similarity measurement, ROC (Receiver Operating Characteristic) diagram, and AUC (Area under the ROC Curve), each of which analyzes various aspects of the algorithm, as follows^2,5^. The qualitative analysis of trees can assess the clustering ability and topology of the resultant trees, demonstrating the method's capacity to appropriately categorize the input data. Analyzing the RF distance and CC similarity metric for the two phylogenetic trees or the distance matrices determines their topological dissimilarity/similarity, considering the corresponding similarity ratios, by investigating the linear relationship between the corresponding branch lengths of the trees (or the distance matrices) produced by the two methods. Finally, the ROC diagram and AUC are primarily used to qualify data classification, and do not assess an exact numerical relationship between similarity scores supplied by the compared methods.

Since each of the aforementioned comparative approaches evaluates PCV from a different perspective, we have reported them all to comprehensively analyze the performance of PCV, against the counterpart alignment-free methods. It is worth noting that we compare PCV with two alignment-free methods, the fuzzy integral-based method and the Euclidean distance method. We chose fuzzy integral-based method, since it is been compared to, and shown to be better than, five other alignment-free method, as reported in^5^. This comparison is achieved in terms of RF distance and CC values. In this manner, indirect comparison of PCV with five other alignment-free approaches, FFP, RTD, CV, NCD, and BBC, can be achieved. For a more comprehensive comparison, ROC values of these methods are depicted in the ROC diagrams, as shown in Fig. 19. Moreover, accuracy of the PCV method is also compared against that of the Euclidean distance approach, which is a word-based method, since it outperforms the reference methods, such as Smith–Waterman, especially in the case of homologous sequences. Furthermore, as a key advantage of providing an alignment-free approach, we can enhance the speed of sequence comparison, especially for large datasets. In this manner, “Runtime analysis” section compares PCV's processing time to that of alternative methods for various datasets.

It should be emphasized that in this study, ClustalW method is chosen as the reference method, with which various alignment-free method have been compared so far. Hence, the outperformance of the PCV approach, over the alternative alignment-free methods, is investigated by comparing its performance metrics against those of the alternative methods, considering ClustalW as the reference method. For a comprehensive study, we also take advantage of two state-of-the-arts alignment-based methods, Clustal Omega and Muscle, as the reference methods in this study. It should be noted that although we report accuracy of the PCV method alongside that of ClustalW and two other references, we do not focus on the accuracy improvement of PCV method against the alignment-based approaches. Rather, the main goal is to provide almost the same accuracy of the alignment-based method by an alignment-free one. Finally, it is worth noting that although ClustalW is not the most accurate alignment-based method proposed so far, it is a popular one and has been used as the reference method for evaluating other alignment-free methods^2,5^. Therefore, for a fair comparison of PCV method with the alternative alignment-free methods, we have also chosen ClustalW as the reference method. However, we incorporated two other reference methods, Clustal Omega and Muscle, to investigate the efficiency of PCV in comparison to the state-of-the-art methods.

### Phylogenetic trees analysis

Studying phylogenetic trees specifies the capability of PCV to properly cluster different samples from various categories. Specifically, this approach, as a qualitative evaluation approach, only studies proper placement of the samples within the subtrees. It should be noted that since some methods produce their phylogenetic trees by UPGMA (Unweighted Pair Group Method with Arithmetic Mean) and some others by NJ (Neighbor-Joining), we have used both methods to produce the trees. All trees produced by these methods are accessible in the supplemental materials, however to avoid overlength paper, depending on the method being compared with PCV, either UPGMA or NJ is reported in the following section. Finally, it should be mentioned that we use MEGAX software version 10.1.7 to produce the trees.

#### 9 ND5 protein sequences

**Figure 7:**
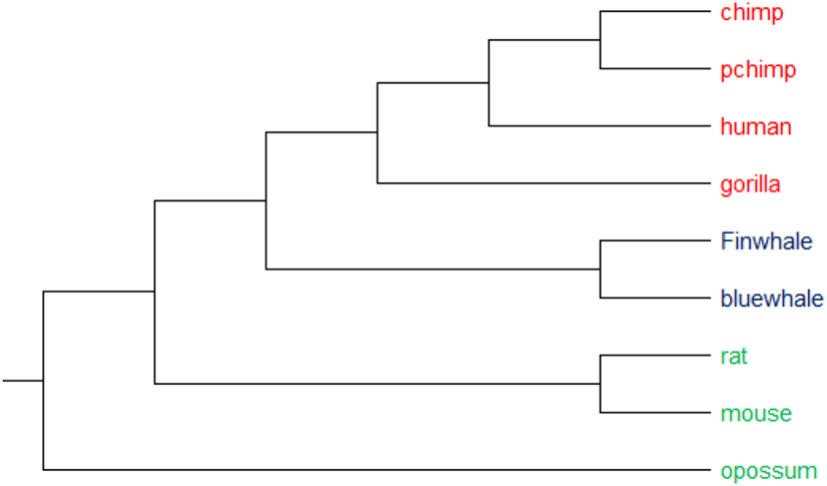
The phylogenetic tree of 9 sequences of NADH Dehydrogenase 5 protein constructed by our method (UPGMA).

MT-ND5 is one of the seven mitochondrial genes encoding subunits of the enzyme NADH dehydrogenase (ubiquinone). This protein is a section of a large enzyme complex, known as complex I, which is active in mitochondria. This enzyme is the largest of the respiratory complexes, and is responsible for the first step of the electron transport process, i.e. the transfer of electrons from a molecule called NADH to another molecule called ubiquinone^5,27,28^. This dataset includes 9 protein sequences of ND5, which approximately has 600 amino acids. The phylogenetic tree (produced by UPGMA method) constructed by our method, PCV, is shown in Fig. 7. These 9 sequences are divided into four categories based on their taxonomic families, (1) including human, pigmy chimpanzee, common chimpanzee, and gorilla, (2) including fin whale and blue whale, (3) including mouse and rat, and (4) including opossum. An opossum is farthest away from other species, hence it is clustered separately. Unlike the phylogenetic tree produced by fuzzy integral algorithm^5^ for this dataset, which is shown in Figure S2, pigmy chimpanzee and common chimpanzee are correctly put close to each other by our method. To elucidate the effectiveness of our approach, we compared the phylogenetic tree generated by our method with the corresponding tree generated by ClustalW^11^ (as shown in Figure S4). This comparison reveals that our method leads to a completely compatible result, although it is an alignment-free method.

#### 8 ND6 protein sequences

**Figure 8:**
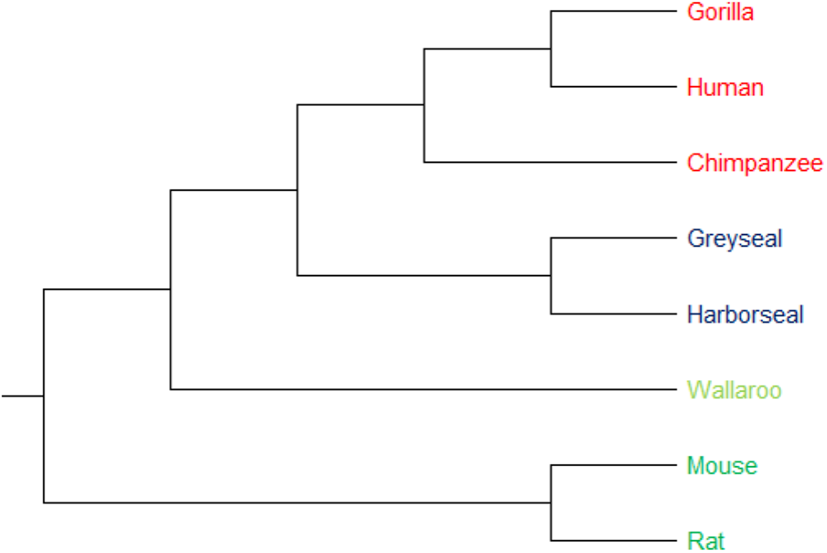
The phylogenetic tree of 8 sequences of NADH Dehydrogenase 6 protein constructed by our method (UPGMA).

MT-ND6, the only protein-coding gene located on the L-strand of the human mitogenome, is one of seven mitochondrial genes encoding subunits of the enzyme NADH dehydrogenase (ubiquinone)^5^. This dataset includes 8 protein sequences of ND6, which approximately has 175 amino acids. The phylogenetic tree constructed by our method is shown in Fig. 8. As shown in this figure, these 8 sequences are divided into four categories based on their taxonomic family, (1) including human, chimpanzee, and gorilla, (2) including gray seal and harbor, (3) including mouse and rat, and (4) including wallaroo. As shown in the phylogenetic tree (produced by UPGMA method) generated by PCV, the protein sequences belonging to the four categories are correctly separated. Indeed, the similarity between wallaroo and (human, chimpanzee, gorilla) and (gray seal, harbor seal) is more than the similarity between (mouse, rat). Therefore, we can conclude that our method successfully clustered wallaroo in same clade with (human, chimpanzee, and gorilla) and (gray seal, harbor seal). The latter observation illustrates the outperformance of PCV over fuzzy integral algorithm^5^, which put wallaroo in the same clade with (mouse, rat), as shown in Figure S6. Finally, it is worth noting that, as shown in Figure S8, the alignment-based approach, ClustalW^11^, generates a phylogenetic tree with the same topology as that of PCV. Hence, we can conclude that PCV can be as accurate as the alignment-based methods.

#### 24 TF protein sequences

**Figure 9:**
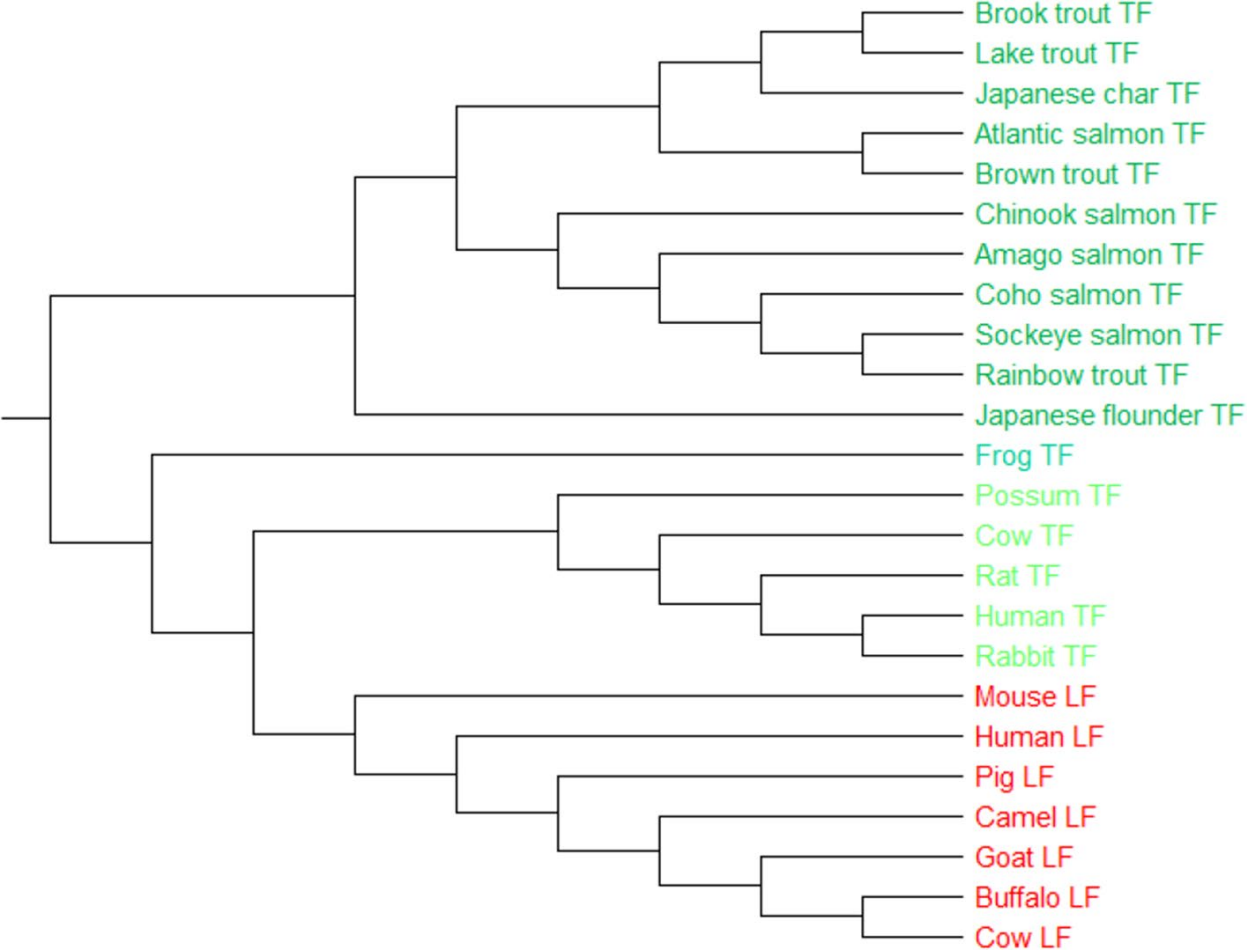
The phylogenetic tree of 24 sequences of transferrins protein constructed by our method (UPGMA).

Protein sequences of transferrins (TFs) from vertebrates, with approximately 700 amino acids, are glycoproteins found in vertebrates that bind to iron (Fe), and consequently, they mediate the transport of iron (Fe) through blood plasma. The liver is the main site of transferrin synthesis, but other tissues and organs, including the brain, also produce transferrin. Transferrin is also associated with the innate immune system. It is found in the mucosa and binds iron, thus creates an environment with low level of free iron that impedes bacterial survival in a process called iron withholding^5,8^. For PCV, both UPGMA and Neighbor-Joining tree construction methods show the precise categorization. Figure 9 shows the PCV-UPGMA tree for the TF dataset. The 24 sequences are split into four clades, as indicated in this diagram, mammalian TF (light green clade), mammalian LF (red clade), actinopterygii (green clade), and amphibians (bluegreen clade). Whereas, the tree produced by fuzzy integral-based method^5^ (Figure S10) clusters the Japanese flounder transferrin sequence (which belongs to the actinopterygii class) with the Frog transferrin sequence (which belonge to amphibians class). Even the tree produced by ClastalW method (Figure S12) does not correctly classify Possum transferrin sequence as a mammalian TF. It should be noted that the NJ tree of the TF dataset produced by PCV also follows the similar trend to the produced UPGMA tree.

#### 24 Coronavirus protein sequences

**Figure 10:**
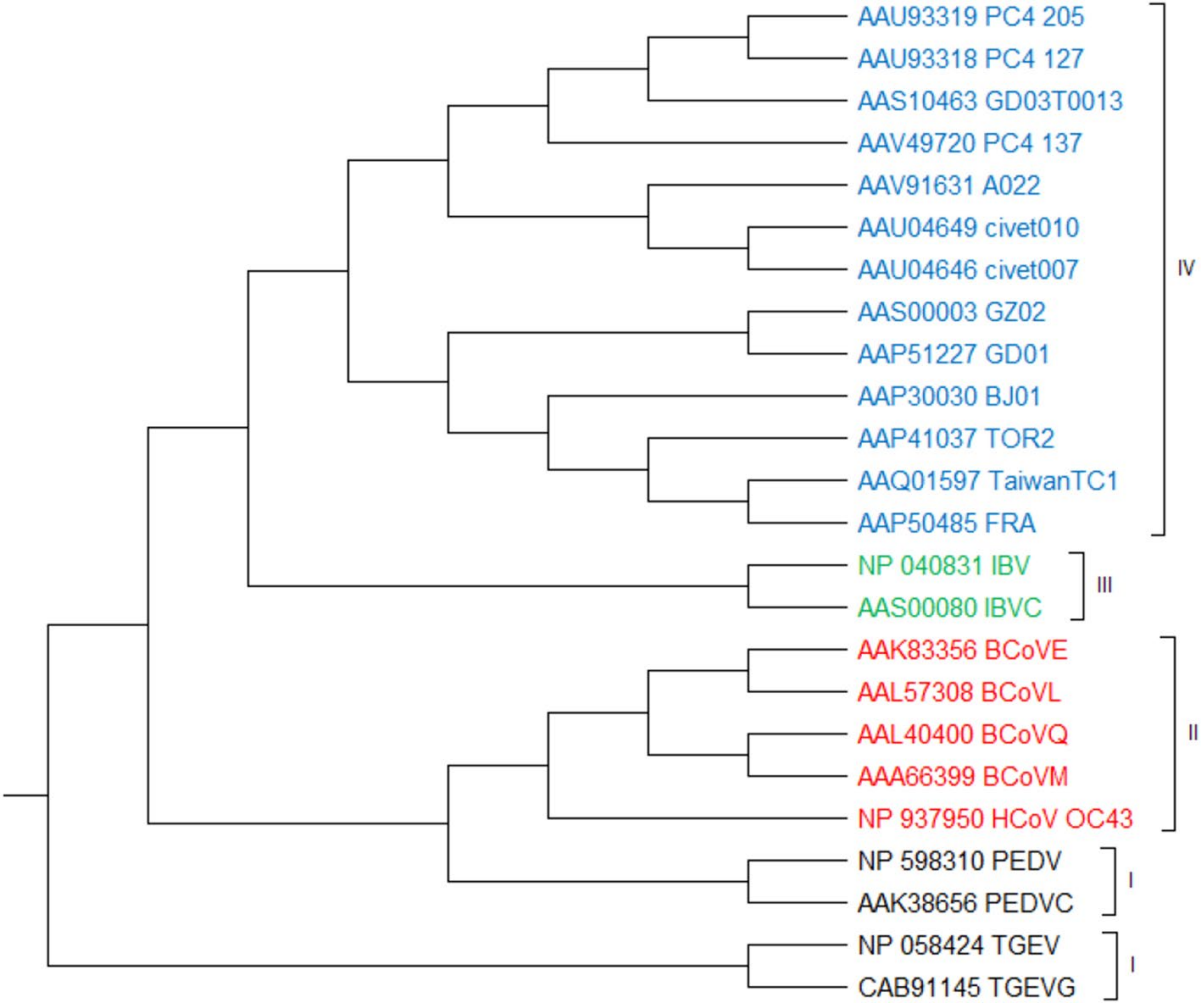
24 Coronavirus protein sequences constructed by our method (UPGMA).

Coronaviruses are large, enclosed, positive-stranded RNA viruses that belong to the Coronaviridae family. Coronaviruses cause respiratory and gastrointestinal disorders in humans and other animals. The spike protein, which is found in all coronaviruses, is essential for viral attachment and entrance into the host cell, while its sequence and structure differ depending on the host^5,7,32^. Coronaviruses can be classified into four types based on their spike proteins and host types. This dataset contains 24 coronavirus protein sequences divided into four classes^5^: classes I (mammalian coronaviruses), II (mammalian coronaviruses), III (avian coronaviruses), and IV (SARS-CoV samples). As shown in Fig. 10, the phylogenetic tree produced by PCV almost correctly categorizes samples of this dataset into the four indicated classes. All samples are accurately classified by PCV, except two samples from class I (i.e. AAK38656 and NP598310), which are added to the top level of class II. However, it should be noted that since they are close to other members of class II and also are separated from class I, it might not be considered as a clustering error. For a comprehensive study, this dataset is also put to the ClustalW^11^ method for comparison. Similar to our method, as shown in Figure S15, ClustalW properly classifies the samples too. It should be mentioned that all phylogenetic trees for this dataset are generated by UPGMA method. This dataset is also used to evaluate other comparison methods, such as the intensity-based method^7^. While this method has not created a phylogenetic tree for this dataset, it uses UPGMA for phylogenetics tree construction for two other datasets (i.e. ND 5 and betaglobin). The intensity-based technique merely presented the average and standard deviation of the produced intensity vectors (Table S11), claiming that the values for distinct groups of coronaviruses are different. However, the boundaries of these values are unclear for each class of this dataset, and hence, a threshold cannot be chosen for classifying this dataset using the intensity-based technique.

#### 50 Coronavirus spike protein sequences

**Figure 11:**
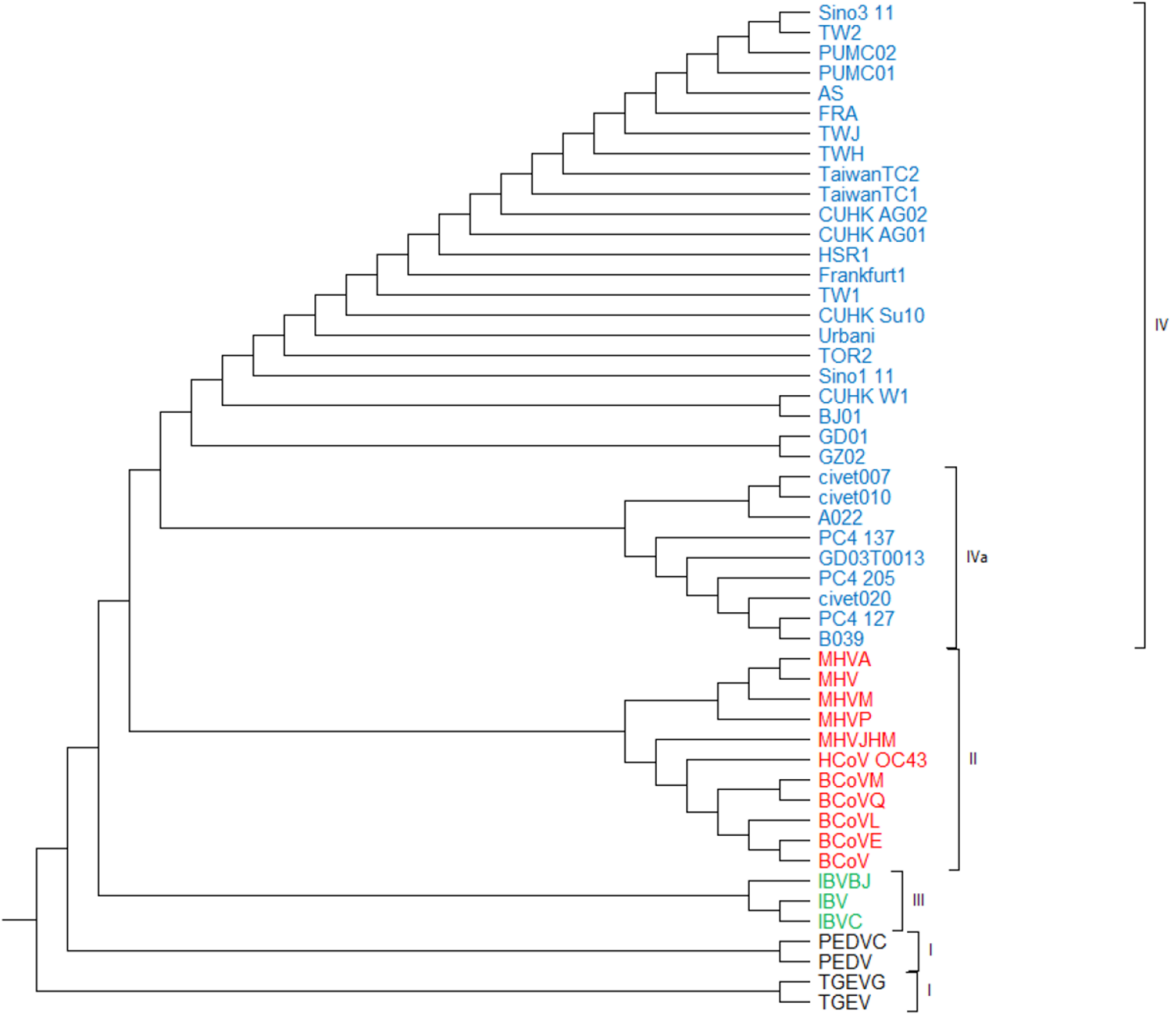
50 Coronavirus protein sequences constructed by our method (UPGMA).

This coronavirus data collection, which includes 50 samples of length 1500 amino acids, is also divided into four groups: Mammalian coronaviruses are classified into different types I and II, avian coronaviruses are found in group III, and SARS-CoVs are found in group IV^5,7,32^. As shown in Fig. 11, the phylogenetic tree produced by PCV properly distinguishes the strings belonging to each group. Furthermore, all strings that are closely connected are classified in the same category within a group. For example, (TGEV, TGEVG) and (PEDVC, PEDV) from class I are clustered as separate clades, or as another example, while the SARS coronavirus group is categorized independently from other coronavirus groups, its subgroups are also categorized as two sub-trees, i.e. group IVa from the 03–04 interspecies epidemic and the one containing all other human-related SARS-CoVs branches^29^. Furthermore, considering coronavirus protein sequences, the classifications achieved by PCV are consistent with those resulted by the ClustalW^11^ method (as shown in Figure S19) and the fuzzy integral based method^5^ (as shown in Figure S17). It is worth noting that these phylogenetic trees are created using the UPGMA approach.

#### 27 Antifreeze protein sequences

**Figure 12:**
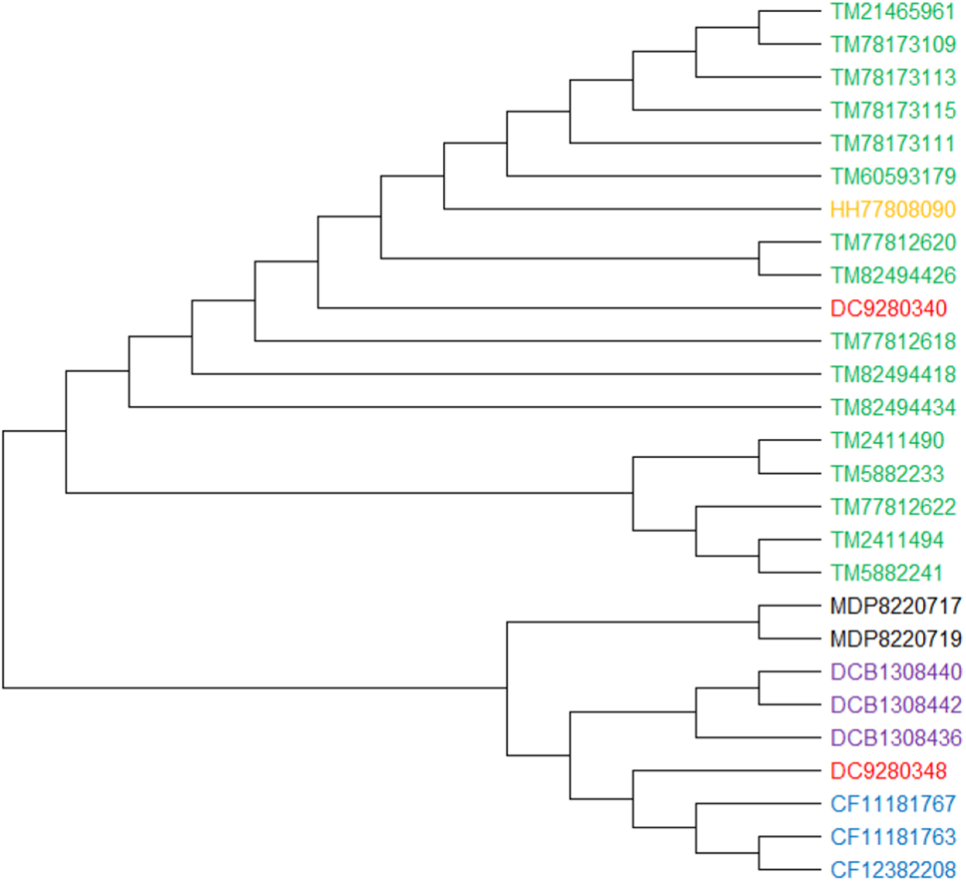
27 Antifreeze proteins (AFPs) sequences constructed by our method (NJ).

Antifreeze proteins (AFPs) are a group of proteins that bind to macromolecular ice and prevent it from accumulating. Spruce budworm (Choristoneura fumiferana, CF), yellow mealworm (Tenebrio molitor, TM), Hypogastrura harveyi (HH), Dorcus curvidens binodulosus (DCB), Microdera dzhungarica punctipennis (MDP), and Dendroides canadensis (DC) are the six species that makeup AFPs^2,27^. The phylogenetic tree^11^ presented in^2^ for ClustalW differs from the corresponding trees we generated using NJ and UPGMA methods. Specifically, as shown in Fig. 12, by comparing PCV’s generated trees with that of ClustalW (Figure S22), reported in^2^, we can observe that only two elements in the "TM" clade of the NJ's PCV phylogenetic tree are classified incorrectly, so our generated tree outperforms the tree presented in^2^. On the other hand, the phylogenetic trees generated for the ClustalW (Figure S23 and Figure S24) provide better results, with some mergers within the three categories "DCB," "MDP," and "TM" visible. Therefore, in comparison with reported results in^2^, PCV achieves more accurate phylogenetic trees. According to the description presented in^2^ for phylogenetic trees analysis (Figure S21), the Energy matrix approach^2^ accurately performs the classification task, while it has not reported any evaluation metric, such as RF distance or AUC, to confirm the accuracy. Finally, it is should be noted that in this manuscript, the aforementioned phylogenetic trees are created using the NJ approach.

#### 9 Betaglobin protein sequences

**Figure 13:**
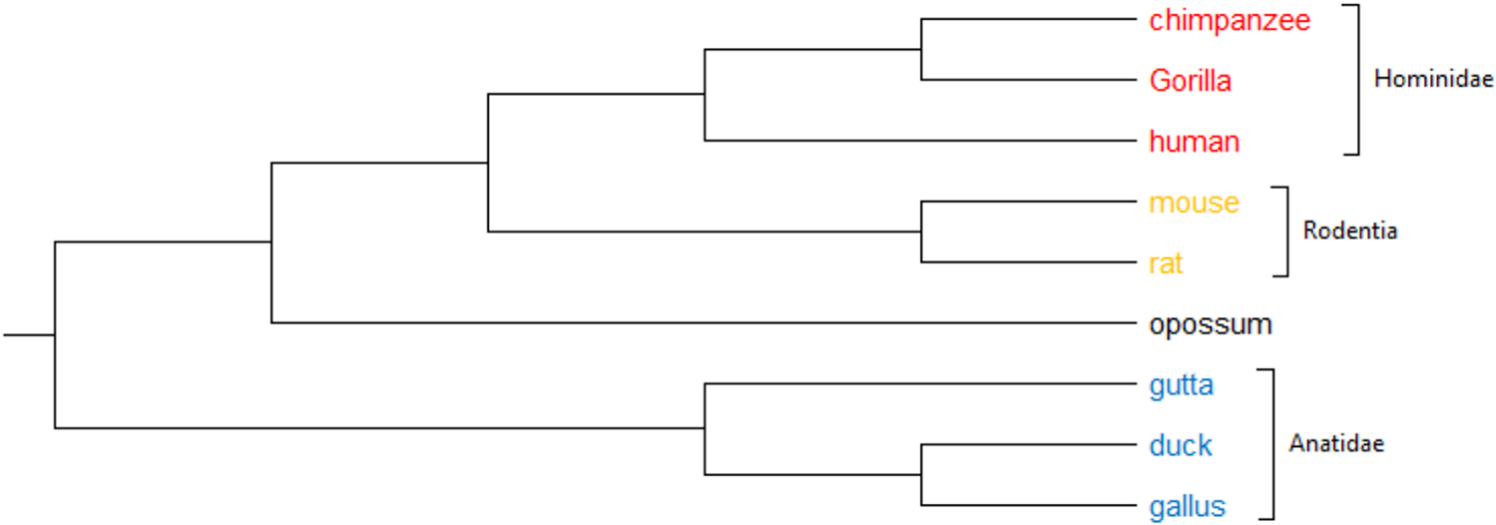
The Phylogenetic tree of 9 betaglobin proteins constructed by our method (UPGMA).

The most prevalent haemoglobin in adult humans is beta-globin, which is frequently used to investigate species’ connections. This dataset is the first version of betaglobin, and includes 9 protein sequences with a maximum length of 147 amino acids from four categories: (1) Hominidae, including Chimpanzee, Gorilla, Human, (2) Rodentia, including Rat, mouse, (3) Didelphidae, including Oppossum, and (4) Anatidae, including Duck, Gutta, Gallus^7,29^. The phylogenetic tree constructed by PCV, based on UPGMA method, is shown in Fig. 13. According to this figure, our method clustered the sequences as accurate as ClustalW^11^ (as shown in Figure S28). For example, the similarity between Gorilla and Chimpanzee is more than that of humans and Chimpanzee, and our method successfully clustered humans after gorilla and chimpanzee. Furthermore, as compared to the alignment-free approaches, such as the Intensity method^7^ (whose generated tree is shown in Figure S26), PCV tends to combine comparable groups together. For example, PCV appropriately groups Rodentia and Hominidae, while the Intensity technique fails to do so, or PCV correctly groups Oppossum with Rodentia and Hominidae, whereas the Intensity method incorrectly groups it with Anatidae.

#### 50 Betaglobin protein sequences

**Figure 14:**
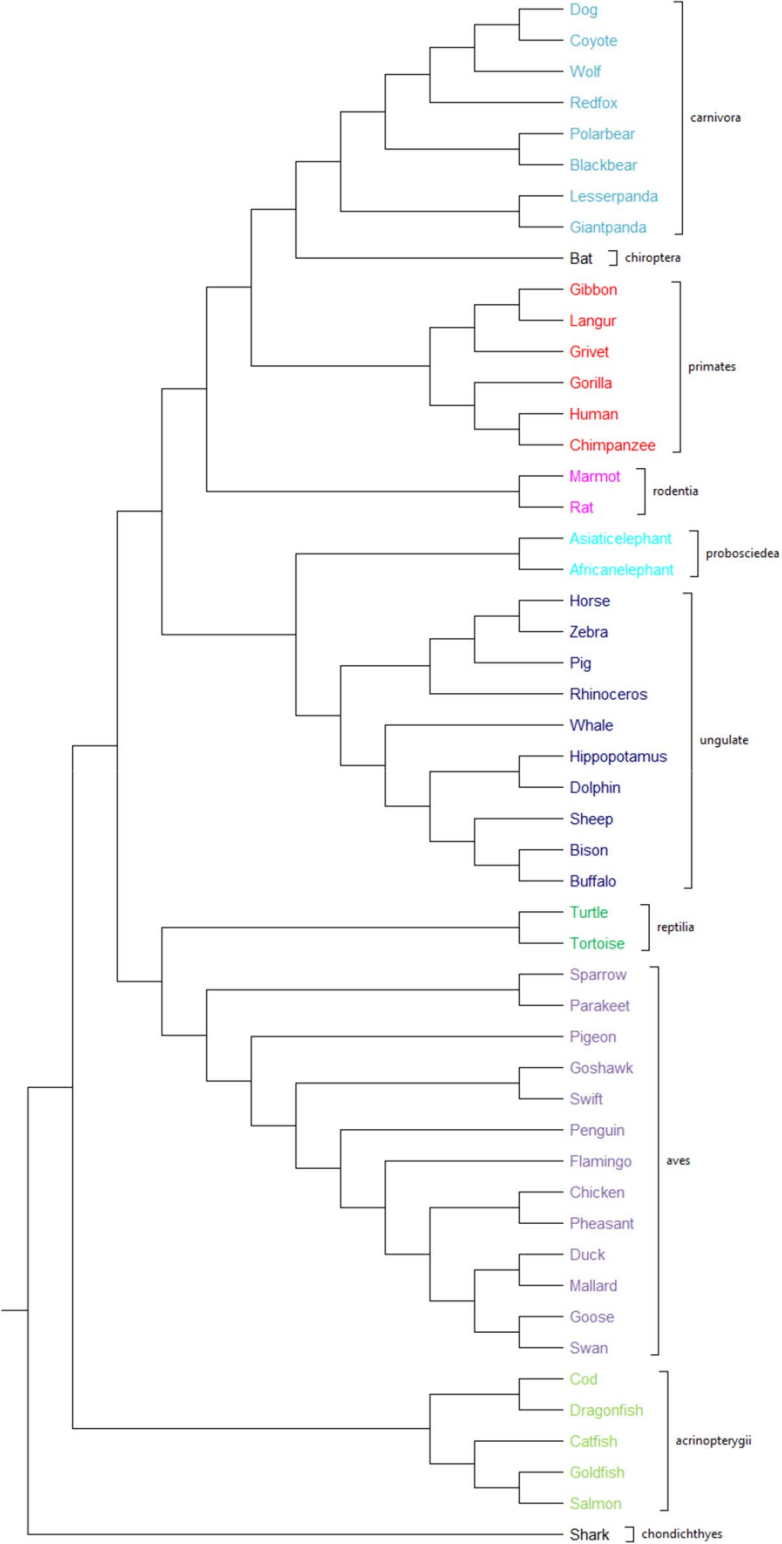
The phylogenetic tree of 50 betaglobin proteins constructed by our method (UPGMA).

According to^5^, these 50 beta-globin protein sequences, from various species taken from GenBank, can be divided into four categories: mammals, birds, reptiles, and aquatic animals. However, the categorization does not end here; there are more details, while each of these classes encompasses multiple subclasses. Specifically, Primates, Proboscidea, Ungulate, Carnivora, Rodentia, Chiroptera, and Cetacea are all mammals; birds or Aves are both categorized as birds; and, Aquatics consist of Actinopterygii and Chondrichthyes^2,5^. As shown in Fig. 14, PCV precisely categorizes these sequences into the same four main categories as previously described. Moreover, PCV has good performance in classifying subclasses while other free-alignments methods, such as fuzzy integral based method^5^ (as shown in Figure S30), lead to some mistaken categorization within classes or subclasses, such as “Antinopterygii” and “Ungulate”. Consequently, PCV outperforms other proposed alignment-free approaches, such as^2,20,33^. Furthermore, PCV's phylogenetic tree is closely related to the ClustalW’s result^11^ (as shown in Figure S32), as an alignment-based approach, and even it outperforms the ClustalW in clustering “Ungulate” class as a separate subtree. It should be noted that for this dataset, phylogenetic trees are generated using the UPGMA approach.

#### 88 Betaglobin protein sequences

**Figure 15:**
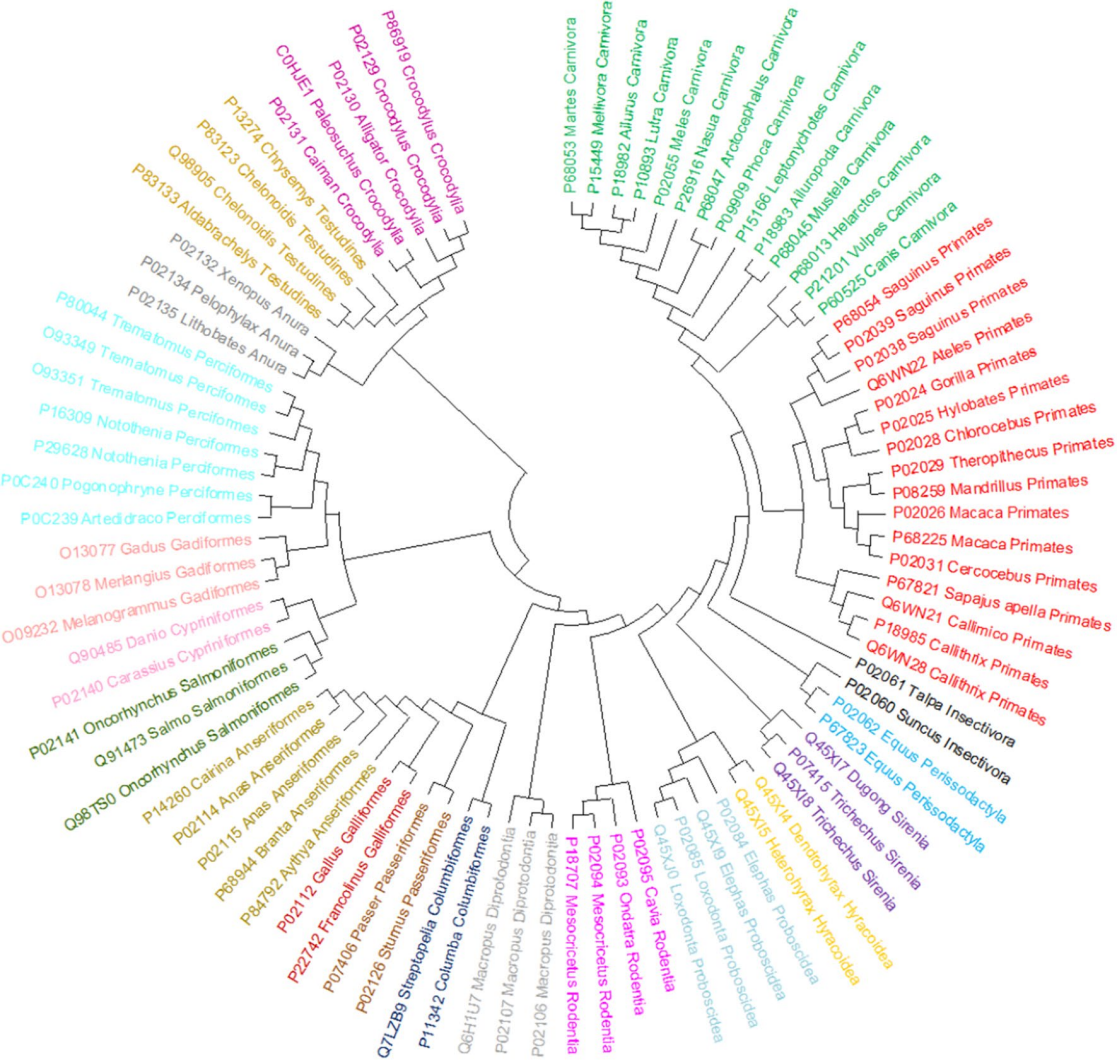
The phylogenetic tree of 88 betaglobin proteins constructed by our method (NJ).

This dataset is related to beta-globin protein data, but it includes 88 samples from 20 distinct categories and a wider range of species, including Carnivora, Primates, Sirenia, Insectivora, Perissodactyla, Hyracoidea, Proboscidea, Rodentia, Diprotodontia, Testudines, Columbiformes, Passeriformes, Galliformes, Anseriformes, Crocodylia, Anura, Perciformes, Gadiformes, Cypriniformes, and Salmoniformes^29^. There is a relationship between some of these 20 categories at the high levels of evolution, just as there was with the previous dataset (i.e. 50 Betaglobin protein sequences). As a result, a classifier strategy with a strong performance is expected to bring these categories closer together. This database is created using the natural vector approach, and the findings are presented in the form of a phylogenetic tree created by NJ. So, for a fair comparison, outputs of PCV for this dataset are used to produce a phylogenetic tree based on the NJ approach. PCV clusters all 20 categories appropriately, as well as groups related categories closer together at the order level, as shown in Fig. 15. Furthermore, PCV’s outcome is very similar to ClustalW^11^ (as shown in Figure S35), as an alignment approach. Moreover, in comparison with ClustalW, it provides better clustering and does not result in any mistake in grouping classes Anseriformes and Rodentia. Finally, the phylogenetic tree produced by the natural vector^29^ (as shown in Figure S34), as a non-alignment method, is as accurate as that of PCV.

#### 20 Xylanase protein sequences

**Figure 16:**
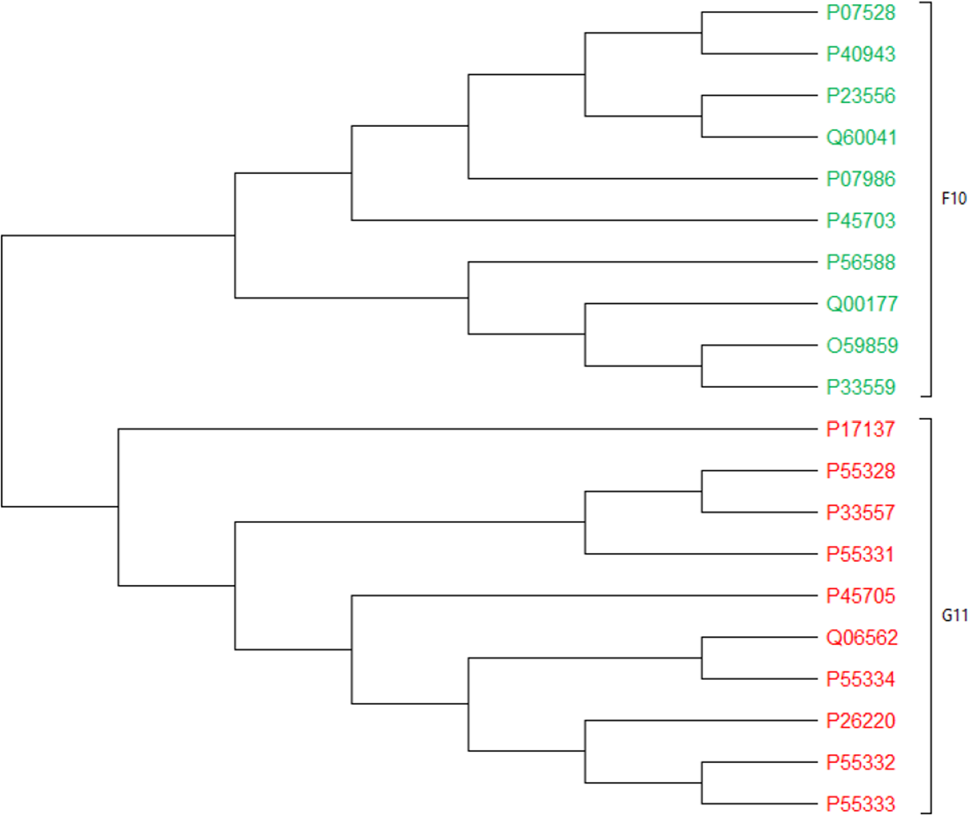
The phylogenetic tree of 20 Xylanase proteins constructed by our method (NJ).

The 20 xylanases protein sequences, which had roughly 500 amino acids, constitute another benchmark dataset utilized to validate the approach. Sequences of this dataset belong to the two classes F10 and G11^5^. As shown in Fig. 16, PCV correctly distinguishes between these two types of samples. It should be noted that although the fuzzy integral based method^5^ (Figure S38), as an alignment-free method, also makes such a distinction between the classes, PCV’s phylogenetic tree appears to be more similar to that of the ClustalW^11^ (Figure S39), as the reference approach. Discussed later, quantitative comparisons, such as the RF distance and the CC measurement, support this claim as well. Finally, it should be noted for Xylanase dataset phylogenetic trees are generated using the NJ approach.

#### 113 Human rhinoviruses (HRV) and 3 HEV-C protein sequences

**Figure 17:**
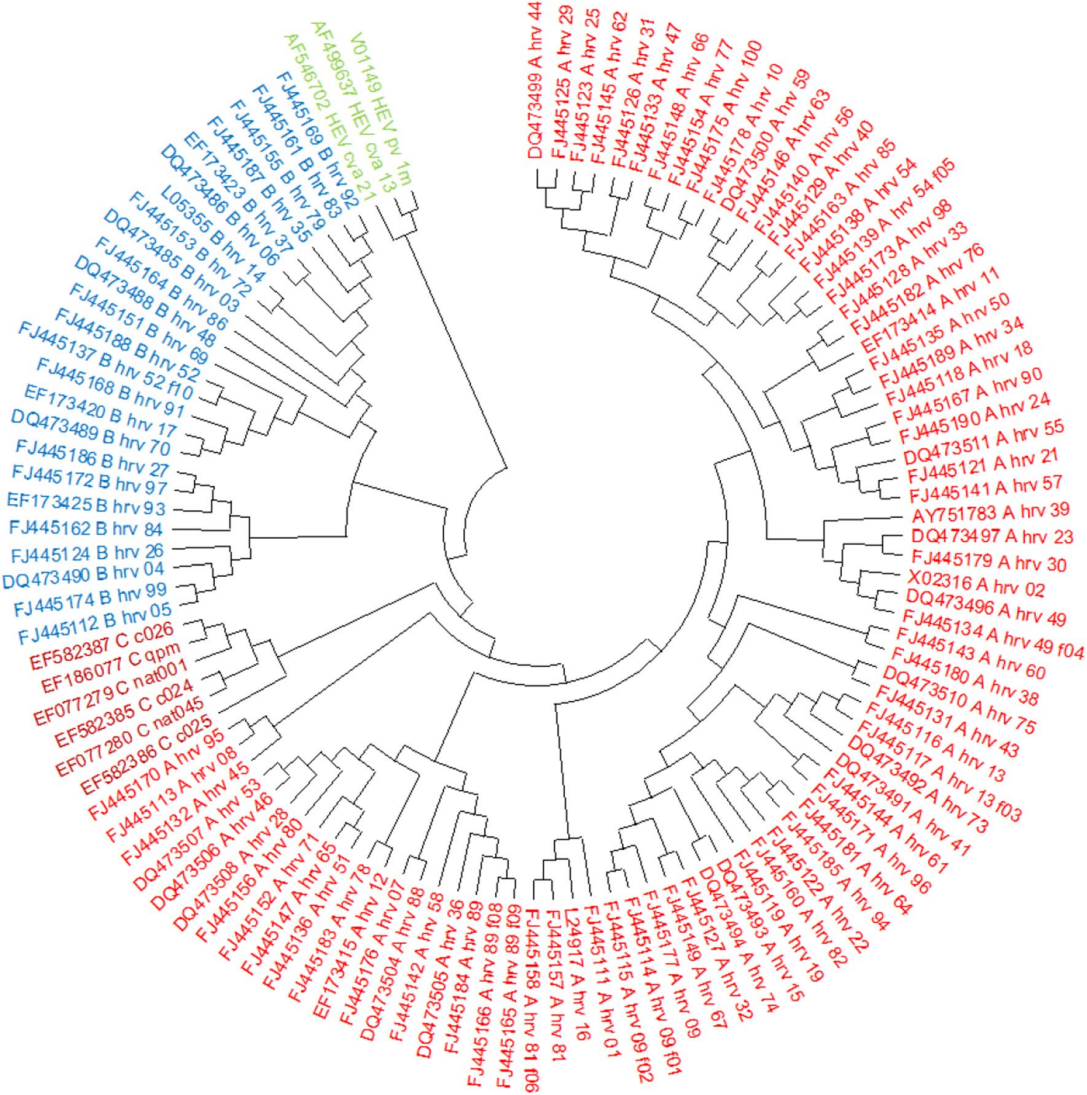
The phylogenetic tree of 113 Human rhinoviruses (HRV) and 3 HEV-C proteins constructed by our method (NJ).

Human rhinovirus (HRV) is one of the most prevalent causes of respiratory infections and is often associated with the common cold. The HRV dataset^29^ contains 113 HRV protein sequences from the RV-A, RV-B, and RV-C Enterovirus genera in the Picornaviridae family, as well as three HEV samples as an outgroup. As a result, phylogenetic trees must categorize this dataset into four clusters. According to Fig. 17, PCV accurately categorizes three HRV species and HEV-C. It resembles the ClustalW’s tree^11^ (as shown in Figure S43), as well as the phylogenetic tree produced by other methods, such as the Natural vector method^29^ (as shown in Figure S42). The NJ technique has been used to create all phylogenetic trees for this dataset.

#### 1163 Influenza A viruses protein sequences

**Figure 18:**
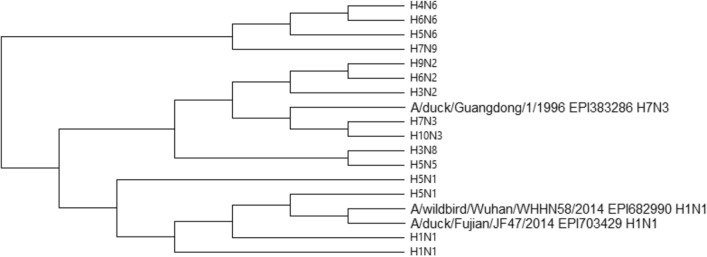
The phylogenetic tree of 1163 Influenza A viruses proteins constructed by our method (NJ).

Influenza A viruses cause influenza in birds and certain mammals, which can result in a range of serious human disorders. Antigenic variation of two surface glycoproteins, i.e. hemagglutinin (HA) and neuraminidase (NA), varies substantially across influenza viruses^29^. As a result, subtypes of influenza A viruses are identified by two numbers H and N, where H represents the hemagglutinin type, which currently has 18 variants, and N represents the type of neuraminidase, for which currently there are 11 variants. Thus, diversity of this dataset emphasizes its key role for evaluating classification methods, including PCV. For this purpose, we used 1163 influenza A viruses NA protein sequences which are divided into 13 subtypes: H5N6, H5N1, H7N9, H1N1, H6N2, H3N8, H3N2, H4N6, H5N5, H10N3, and H7N3. As shown in Fig. 18, PCV precisely categorizes 13 varieties of influenza A viruses, while the sequences with the same N number are grouped together. The resultant phylogenetic tree demonstrates that our proposed method is capable of accurate categorizing even for enormous datasets. It should be noted that PCV’s categorization is in line with ClustalW’s findings^11^ (as shown in Figure S47) and natural vector method^29^ (as shown in Figure S46). Furthermore, our phylogenetic tree appears to be a bit better than theirs, with more sequences clustered inside each clade and fewer separated samples, compared to phylogenetic trees produced by ClustalW and natural vector method. Finally, as shown in Fig. 18, PCV brings related clades together. The NJ technique has been used to create all phylogenetic trees for this dataset.

### Robinson Foulds (RF) distance results

The Robinson–Foulds (RF) distance is a commonly used comparison metric for analyzing the linear relation between two phylogenetic trees. The RF distance of zero indicates that the trees are identical; as the distance increases, the trees become less similar^5^. As presented in this section, we built phylogenetic trees using both the UPGMA and NJ methods, and for each method, we reported RF distance between the phylogenetic trees produced by our method (i.e. PCV) and three alignment-based method, the ClustalW^11^, Clustal Omega^18^, and Muscle^12^, as the reference method. Furthermore, while some other studies, such as the fuzzy integral-based technique^5^, provide RF distances for their proposed methods, we have also included those information in Table 3 as well, so we can compare our proposed method to some other alignment-free methods. According to this table, for all provided datasets, PCV outperforms fuzzy integral-based approach and also outperforms the squared Euclidean distance approach except for one of them (Xylanase) with small differentiate, and its resultant trees are more compatible with those of ClustalW approach. More detailed discussions of this table are provided in the "Discussion" section.

**Table 3:** Comparison of PCV (both UPGMA and NJ trees), ED and Fuzzy integral based method based on Robinson Foulds (RF) distance for 12 benchmark datasets.

Dataset/method	PCV_UPGMA_ and ClustalW	PCV_NJ_ and ClustalW	FI^5^ and ClustalW	ED_NJ_^34^ and ClustalW	PCV_NJ_ and Clustal Omega	FI_NJ_ and Clustal Omega	ED_NJ_^34^ and Clustal Omega	PCV_NJ_ and Muscle	FI_NJ_ and Muscle	ED_NJ_^34^ and Muscle
ND5	**0**	2	2 (Fitch-Margoliash)	4	0	2	4	**0**	2	4
ND6	**0**	4	2 (Fitch-Margoliash)	2	4	4	2	4	4	2
TF	**8**	12	20 (Fitch-Margoliash)	16	10	20	14	**10**	20	14
Coronavirus (24)	**4**	**6**	–	–	**10**	–	–	**8**	–	–
Coronavirus (50)	**28**	**40**	46 (Fitch-Margoliash)	–	**36**	46	–	**40**	46	–
AFP	**34**	**34**	–	–	**34**	–	–	**32**	–	–
Beta globin (9)	**2**	**2**	–	2	**2**	–	2	**2**	–	2
Beta globin (50)	**30**	**32**	64 (UPGMA)	–	**32**	64	–	**32**	64	–
Beta globin (88)	**68**	**60**	–	–	**72**	–	–	**72**	–	–
Xylanase	18	16	18 (Fitch-Margoliash)	12	18	22	14	16	20	14
HRV	**24**	**30**	–	–	**38**	–	–	**38**	–	–
Influenza A	**1550**	**1578**	–	–	**1666**	–	–	–	–	–

Significant values are in bold.

### Correlation Coefficient (CC) results

Another well-known metric for measurement of linear relationships between two vectors is the Correlation Coefficient (CC)^5,28^. The value of this metric is in the range of [− 1,1], where the greater the linear relationship between two vectors, the closer CC value is to 1 or − 1, and the weaker the relationship, the closer it is to zero. The direct and indirect connection of the two vectors, respectively, is presented by positive and negative values. In this manner, for each of the 12 datasets, we utilized CC to examine the linear relationship between the distance matrix of PCV and three alignment-based methods ClustalW^11^, Clustal Omega^18^, and Muscle^12^; Table 4 shows these results. According to this table, the average CC metric for all datasets is above 94%, indicating that our suggested approach has been able to largely conform to alignment-based methods and outperforms the fuzzy integral-based method^5^ for the given datasets and also outperforms ED for most of datasets. More detailed discussions of this table are provided in the "Discussion" section.

**Table 4:** Comparison of PCV, Fuzzy integral based method, and ED method based on Correlation Coefficient (CC) distance with ClustalW, Clustal Omega, and Muscle for 12 benchmark datasets.

Dataset/method	PCV and ClustalW	FI^5^ and ClustalW	ED^34^ and ClustalW	PCV and Clustal Omega	FI^5^ and Clustal Omega	ED^34^ and Clustal Omega	PCV and Muscle	FI^5^ and Muscle	ED^34^ and Muscle
ND5	**0.984**	0.738	0.9250	0.984	0.801	0.986	0.984	0.918	0.986
ND6	**0.979**	0.598	0.9583	**0.978**	0.896	0.969	**0.979**	0.899	0.970
TF	0.981	0.745	0.9878	0.983	0.938	0.989	0.982	0.938	0.988
Coronavirus (24)	**0.986**	–	–	**0.955**	–	–	**0.989**	–	–
Coronavirus (50)	**0.983**	0.956	–	**0.986**	0.875	–	**0.987**	0.875	–
AFP	**0.857**	–	–	0.873	–	–	0.867	–	–
Beta globin (9)	0.955	–	0.9888	0.955	–	0.988	0.955	–	0.988
Beta globin (50)	**0.914**	0.729	–	**0.907**	0.875	–	**0.907**	0.875	–
Beta globin (88)	**0.943**	–	–	**0.943**	–	–	**0.943**	–	–
Xylanase	**0.909**	0.700	0.8863	0.932	0.941	0.911	0.919	0.937	0.902
HRV	**0.973**	–	–	**0.780**	–	–	**0.780**	–	–
Influenza A	**0.912**	–	–	**0.977**	–	–	–	–	–

Significant values are in bold.

### Receiver Operating Characteristic (ROC) and AUC results

The ROC (Receiver Operating Characteristic) curve is a graphical representation of the diagnostic capabilities of a binary classifier system when its discrimination threshold varies. It can, however, be used for a multi-class classifier as well. The ROC curve is produced by the true positive rate (TPR), also known as sensitivity, recall, or probability of detection, against the false positive rate (FPR), also known as the probability of false alarm and computed as (1—specificity). In addition, the AUC (Area under the ROC Curve) measure is one of the most prevalent interpretations based on the ROC curve. AUC is in the range of [0, 1], while the closer value to 1 indicates the classification approach with the greater accuracy. Generally, an approach with an AUC above 0.9 is considered as a high accurate classifier, while AUC between 0.7 and 0.9, and between 0.5 and 0.7 represent classifiers with average and low accuracy, respectively^5,35^. More information about this curve can be found in the "ROC" section of the supplementary materials.

**Figure 19:**
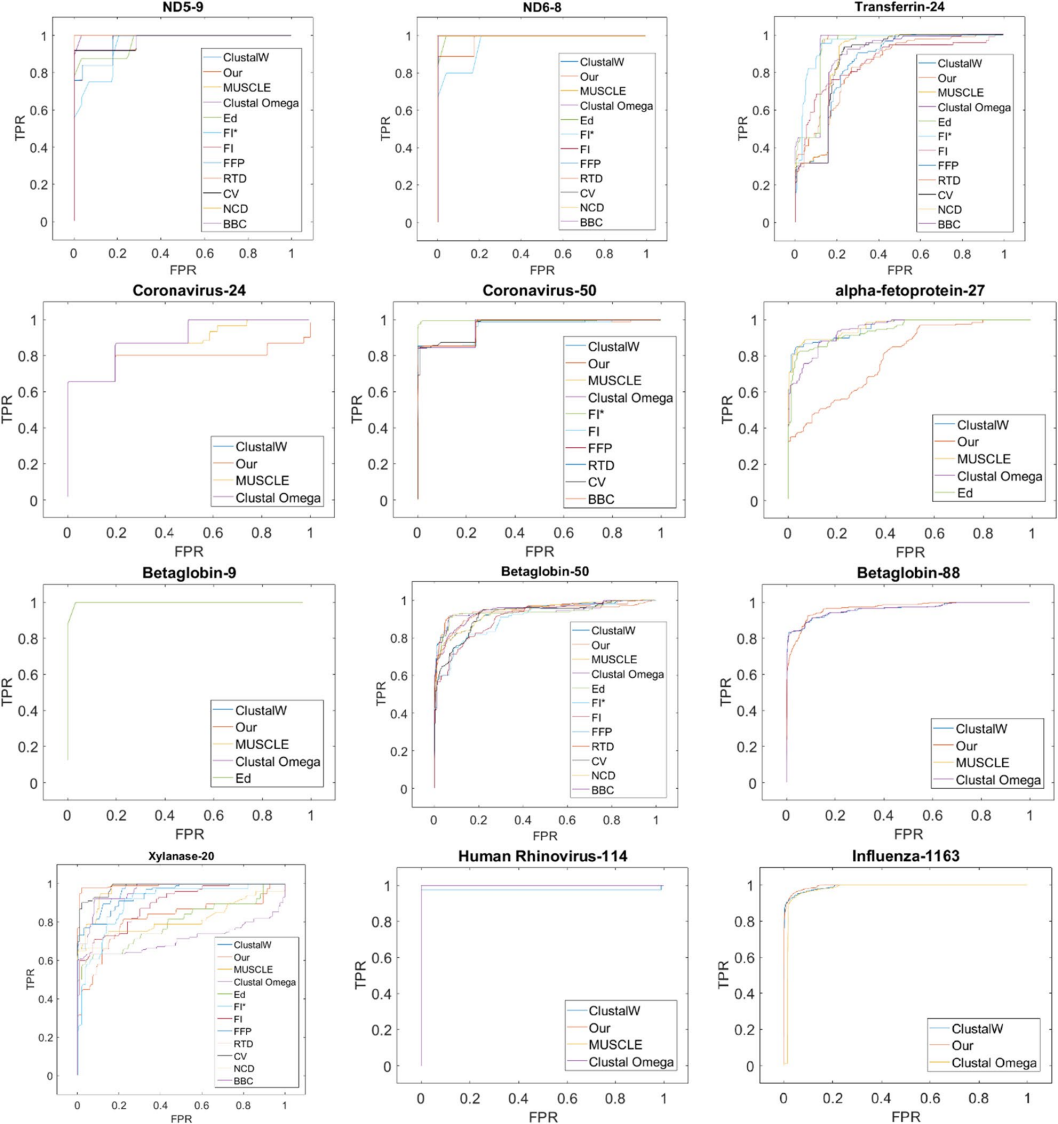
ROC of 12 benchmark datasets.

To evaluate the proposed algorithm, for all 12 datasets, we generated ROC curves and computed their AUC values for PCV, ED^34^, and three alignment-based methods ClustalW^11^, Clustal Omega^18^ and Muscle^12^. Moreover, all ROC curves and AUC values reported for fuzzy integral based method^5^ are also considered in Table 5. It is worth noting that the ROC curves of five other methods, including FFP, RTD, CV, NCD, and BBC, are also provided in^5^. Based on all information reported in Fig. 19 and Table 5, considering AUC as an interpretable metric, PCV is one of the most accurate clustering algorithms, except for two datasets AFP and Xylanase, even more accurate in some datasets like Coronavirus (24) than alignment-based methods, Muscle and Clustal Omega. Moreover, PCV has an AUC value of more than 0.9 representing its clustering capability. AUC value of the fuzzy integral based approach^5^, which in most datasets is greater than the AUC values of ClustalW and PCV methods, should be discussed. As a key point to be noticed in this section is that the classification matrix used to calculate AUC has a high impact on its value, while these matrices are identical for both ClustalW and PCV methods. As a result, their AUC values confirm the trend of other metrics, such as the CC between PCV and ClustalW, as expected. However, the classification matrix of fuzzy integral based approach is ambiguous, and it most likely differs from the matrix we utilize, resulting in a higher AUC score, compared to the ClustalW method, while other metrics indicate that it is inferior to ClustalW.

**Table 5:** Comparison of PCV, ED, Fuzzy integral based method and three alignment-based methods ClustalW, Clustal Omega, and Muscle based on AUC values for 12 benchmark datasets.

Dataset/method	PCV	FI^5^	FI^5^ (based on our classification matrices)	ED^34^	ClustalW	Muscle	Clustal Omega
ND5	**0.9636**	0.97	0.9124	0.9312	0.9636	0.9636	0.9636
ND6	**0.9549**	0.98	0.9180	**0.9549**	0.9549	0.9549	0.9549
TF	0.9242	0.85	0.9481	0.9262	0.9294	0.9294	0.9294
Coronavirus (24)	**0.9631**	–	–	–	0.9945	0.8711	0.8884
Coronavirus (50)	0.9549	0.96	0.9929	–	0.9620	0.9620	0.9620
AFP	**0.7892**	–	–	–	0.9536	0.9581	0.9431
Beta globin (9)	**0.9646**	–	–	**0.9646**	0.9646	0.9646	0.9646
Beta globin (50)	**0.9468**	0.91	0.9009	–	0.9398	0.9385	0.9385
Beta globin (88)	**0.9672**	–	–	–	0.9605	0.9608	0.9608
Xylanase	0.8086	0.89	0.8941	0.7894	0.9554	0.9643	0.9566
HRV	**0.9997**	–	–	–	0.9772	0.9997	0.9997
Influenza A	**0.9939**	–	–	–	0.9912	–	0.7034

Significant values are in bold.

To resolve the aforementioned issue, considering the distance matrix provided by the fuzzy integral based method at^5^, we constructed ROC curves for this method using our classification matrix. In this manner, we achieved two ROC curves for the fuzzy integral approach (the one published at^5^ and the other one we generated from the provided matrix). As reported in Table 5, AUC values for these two curves are computed. It should be noted that the classification matrix reported at^5^ is still used for the other five alignment-free methods. Given the ROC curves and the repetition of the ROCs production for the fuzzy integral based method and their AUC values, we can conclude that our classification matrices are more rigid and reliable, and hence, it is possible that the computed AUC values based on our classification matrix for five alternative alignment-free methods be lower than the corresponding reported values at^5^. Finally, as a key advantage of our proposed method, it should be noted that PCV provides better classification results for large datasets, such as influenza A and betaglobin (88), and HRV, compared to the alternative alignment-free methods as well as alignment-based methods, ClustalW, Clustal Omega, and Muscle. As analysis of quantitative metrics confirm this superiority, the latter achievement is also obvious from qualitative study of the corresponding phylogenetic trees. More detailed discussions of this table are provided in the "Discussion" section.

### Runtime analysis

**Figure 20:**
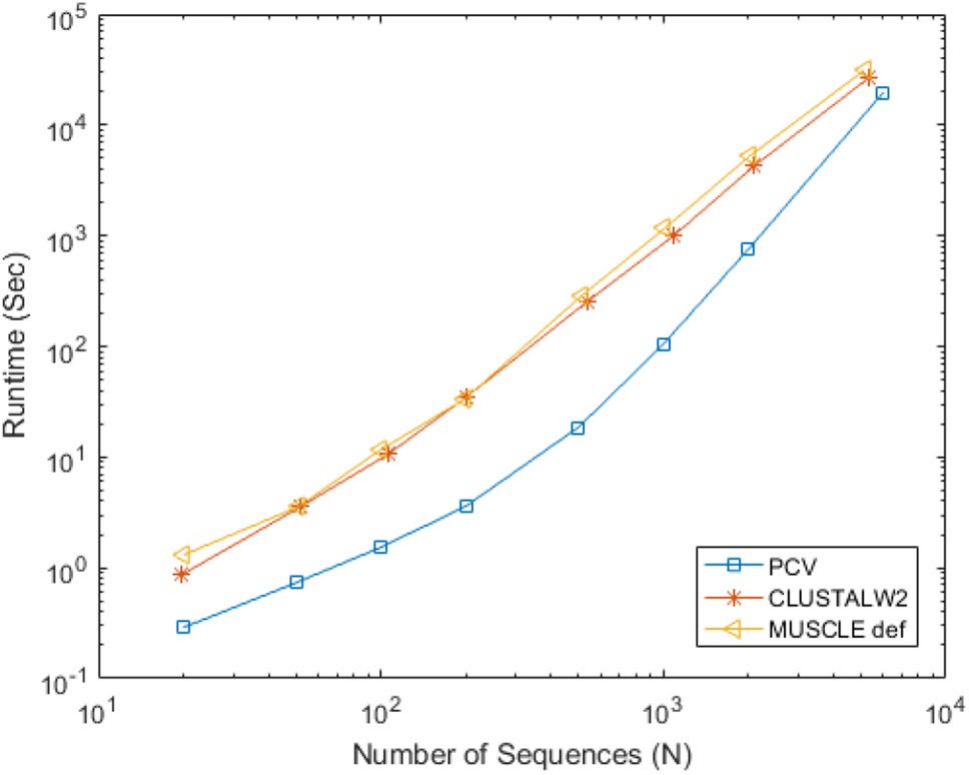
Execution time comparison of three sequence comparison methods, PCV, ClustalW2, and Muscle, for various numbers of input sequences. The sequences in this database are around 330 characters long, and execution times are measured on a system with a 4-core CPU running at 2.4 GHz and 12 GB of RAM.

Considering 12 aforementioned datasets with different number of sequences and varying sequence lengths, in this section, we evaluate the execution time of PCV. For a comparative study, the implementations of ClustalW^11^, as an alignment-based algorithm, and the fuzzy integral based approach^5^, as a free-alignment method, have been presented as well. The corresponding execution times are listed in Table 6. It should be noted that due to the lack of access to FI tool, we only report the execution times reported in^5^, and so, the execution times for the datasets not examined by FI tool are not applied. It should be mentioned that, in addition to the method's execution time, the system’s specifications executing the algorithm should be presented as well. Saw et al. ^5^ uses a Linux server with 24 dual-core processors, 384 GB RAM, and two threads to run its fuzzy integral based technique. While execution times of PCV and ClustalW are measured on a Windows system with a 4-core CPU running at 3.6 GHz and 12 GB of RAM. Considering above specifications, assuming execution of fuzzy integral based technique on a similar system implementing PCV and ClustalW methods, its runtime would be several times higher. Moreover, it should be noted that in order to report the ClustalW's execution time, we ran it 20 times on the system and averaged their results. According to Table 6, for large datasets, either in terms of sequence length or number of sequences, the superiority of PCV’s speed over alignment-based and alignment-free methods is obvious, even for a non-optimized implementation in MATLAB. Figure 20 does a better job of addressing this point. The execution time of the PCV method, as well as those of the Muscle and ClustalW methods^36^, is depicted in this diagram for a various numbers of input sequences. As depicted in this figure, the priority of PCV in terms of execution time for a large number of sequences is crystal clear. For example, PCV can compare around 5000 input sequences in about 28 h, while the other two approaches take more than 84 h to accomplish this comparison. It should be noted that the sequences in this database are around 330 characters long, and execution times are measured on a system with a 4-core CPU running at 2.4 GHz and 12 GB of RAM. Finally, in section "time estimates" of the supplementary materials, we provided a runtime estimation formula of PCV, based on the implementing system’s specification and maximum sequence length.

**Table 6:** Running time of PCV, ClustalW, and fuzzy integral based method.

Dataset	Max Seq. Len	No. of seq	PCV (s)	ClustalW (s)	Speed up (PCV/ClustalW)	FI(s)^5^	Speed up (PCV/^5^)
ND5	610	9	0.2928	0.4802	1.6399	1	3.4153
ND6	175	8	0.0221	0.0682	3.0865	1	45.2489
TF	717	24	1.0784	2.6070	2.4175	4	3.7092
Coronavirus (24)	1447	24	4.3662	8.9456	2.0488	–	–
Coronavirus (50)	1447	50	9.1214	28.5361	3.1285	16	1.7541
AFP	138	27	0.0472	0.2308	4.8894	–	–
Betaglobin (9)	147	9	0.0177	0.0615	3.4765	–	–
Betaglobin (50)	148	50	0.1026	0.7146	6.9648	15	146.1988
Betaglobin (88)	148	88	0.193	1.8662	9.6693	–	–
Xylanase	484	20	0.4115	0.5040	1.2249	3	7.2904
HRV	2214	114	49.0252	303.7247	6.1953	–	–
Influenza A	472	1163	158.7319	1409.5072	8.8798	–	–
Average	678.9167	132.1667	–	–	**4.4684**	–	**34.6028**

Significant values are in bold.

## Discussion

As discussed in this paper, it is critical to provide an accurate and high-speed tool, as a means of protein sequence comparison, which provides sequence categorization, particularly for large datasets with a significant number of sequences. Proteins are sequences whose folded structures have a direct effect on their functions, and their structures are influenced by physicochemical qualities on the one hand^26,27^. As a result, effective physicochemical qualities combined with sequence information can be utilized to increase the accuracy of comparison tools and form the basis for their application in other tools, such as predicting the second and third structures and the protein function. PCV is created and developed with these scenarios in mind.

As previously stated, evaluations and comparisons with current acceptable tools are required to assure PCV’s performance, and these are carried out at several levels. We employed numerous comparison methods, since each method offers a distinct comparison approach. In comparison to the fuzzy integral based technique and ED^34^, the phylogenetic trees of PCV offer substantially much similarity with those of ClustalW^11^, Clustal Omega^18^, and Muscle^12^ based on the RF distance. For this comparison, the topology and branch lengths of the trees are addressed for similarity measurement of phylogenetic trees, assuming that the reference tree is completely correct. On the other hand, since alignment-based metrics are still being studied, it is not definitive to choose an alignment-based method as the reference one. As a result, for some datasets, such as Influenza A and HRV, due to the large number of samples and the less likely exact similarity of the phylogenetic tree of PCV to the reference one, PCV results in large values of RF distance for some datasets. Since no other approach has supplied RF distance for these challenging datasets, PCV evaluation for these datasets is impossible.

We performed a comparative study of PCV and three alignment-based methods, ClustalW, Clustal Omega, and Muscle using the CC similarity metric. For this purpose, we eased the similarity conditions of phylogenetic trees by examining the linear relationship between our method's distance matrix and that of the reference method. In comparison to the fuzzy integral-based method^5^, PCV is improved greatly in terms of this parameter, with an average CC value of 95%. Another measure for evaluating classification capability of a method is the ROC curve. Regarding this measurement, PCV showed to be an accurate classification approach for most datasets. In addition to all these achievements in accuracy comparison, our method is also able to determine the local similarity for each pair of blocks, as well as the overall similarity of the sequences indicated by the final dissimilarity score.

It should be noted that most studies addressing the protein sequence alignment-free comparison^2,5^, with no explicit statement, involve customized datasets containing a number of sequences from specific categories, often at the taxonomy levels of family, genus, or species. In other words, to prepare a customized dataset, an extensive search is performed within a large database to select the proper sequences and create the required datasets. It should be mentioned that for the alignment-free methods, the initial assumption is that the input sequences do not include large structural variations; in other words, they are homologous, so that a one-way comparison can be performed from the beginning to the end of the sequences. Moreover, the assumption can be equated with the proposition that along with the alignment-free methods, a simple sequence search method is required to detect the homologous data. Therefore, we can conclude that generally, the alignment-free methods can process the datasets of homologous sequences, unless joined with some preprocessing tools. To validate the aforementioned statement, for each alignment-free comparison tool, we determined its best response assuming the least identity of the input sequences and the minimum degree of sequence alignment. In this manner, we obtained Table 7 by analyzing the identity matrix of datasets used by each comparison tool. As reported in this table, each dataset is evaluated using three reference methods (i.e. ClustalW, Clustal Omega, and Muscle), and the minimum, maximum, mean, and standard deviation values of each dataset are reported. It should be noted that due to the large number of sequences in the Influenza A dataset, the aforementioned values for this dataset could only be calculated by the Clustal Omega method. Finally, based on the values reported in Table 5, which provides the AUC value of the PCV method for each dataset, we can conclude that the PCV method leads to the accurate classification results for all these datasets, and therefore, it is applicable for the wide range of minimum identity values, reported in Table 7 (i.e. greater than 5% based on Muscle as the reference method). However, choosing the 90% threshold value of AUC, according to the Table S9, we can conclude that the PCV method provides the best sequence comparison output for mean identity values greater than 60% (based on Muscle as the reference method) with the standard deviation of less than 23%.

**Table 7:** Degree of sequence alignment of each dataset, as calculated by the means of sequence identity metric.

Dataset		ClustalW (%)	Clustal Omega (%)	Muscle (%)
ND5	Min	60.46	60.54	60.87
	Max	96.53	96.53	96.53
	Mean	70.3613	73.7072	73.7316
	Standard deviation	11.5634	14.3868	14.3661
ND6	Min	54.49	41.1	40.85
	Max	100	97.14	97.14
	Mean	72.8013	63.9891	64.2753
	Standard deviation	15.7091	21.0188	20.8307
TF	Min	41.94	43.73	43.54
	Max	96.23	96.24	96.24
	Mean	57.64	60.8482	60.8940
	Standard deviation	16.7344	17.4347	17.418
Coronavirus (24)	Min	19.22	25.29	21.94
	Max	100	100	100
	Mean	49.2039	54.1814	54.232
	Standard deviation	34.9317	33.4833	33.4804
Coronavirus (50)	Min	18.38	25.07	24.11
	Max	100	100	100
	Mean	58.125	61.1693	61.6433
	Standard deviation	36.1938	34.2589	33.797
AFP	Min	8.73	11.46	5.88
	Max	100	100	100
	Mean	59.6171	64.1205	64.0066
	Standard deviation	27.2835	25.6215	26.8949
Beta globin (9)	Min	63.26	63.27	63.27
	Max	99.17	100	100
	Mean	75.3489	78.5212	78.5212
	Standard deviation	10.5410	12.6448	12.6448
Beta globin (50)	Min	35.46	33.33	33.33
	Max	100	100	100
	Mean	71.1996	71.6962	71.6962
	Standard deviation	15.5849	16.13405	16.13405
Beta globin (88)	Min	34.24	34.25	34.25
	Max	100	100	100
	Mean	65.4936	65.8985	65.8985
	Standard deviation	16.7106	17.0113	17.0113
Xylanase	Min	5.74	8.57	5.33
	Max	100	100	100
	Mean	25.0495	32.9813	32.6394
	Standard deviation	21.359	24.1594	24.1607
HRV	Min	46.68	13.13	47.51
	Max	99.9	99.91	99.91
	Mean	66.2275	65.9223	66.9712
	Standard deviation	15.4414	16.6548	15.4171
Influenza A	Min	–	5.66	–
	Max		100	
	Mean		63.2029	
	Standard deviation		22.628	

Three different methods, ClustalW, Clustal Omega, and Muscle, are used to calculate identity matrices.

In addition to the issue of accuracy, high speed processing is one of our main goals in designing a suitable method for comparing protein sequences. Examining the speed of our method compared to those of ClustalW, ClustalW2, Muscle and fuzzy integral based method^5^, we can conclude that in the case of large datasets, either in terms of sequence lengths or number of sequences, we can offer a higher processing speed.

Finally it should be mentioned that the alignment-based methods, such as ClustalW, are known as the accurate comparison tools, and have been used for many years. Therefore, in this study, ClustalW was chosen as a reference method to assess the accuracy of the PCV and the alternative alignment-free methods. ClustalW, as mentioned in "Runtime analysis" section, performs calculations for small and medium datasets in an acceptable period of time, and produces auxiliary outputs, like alignment output, in addition to the comparison score. However, it should be noted that in many applications, such as clustering input sequences or searching them within the databases, these auxiliary outputs are not required, and only the comparison score is taken into account. Moreover, in cases when the datasets are particularly large, execution of the alignment-based approaches are not feasible or reasonable for everyone, as seen in Fig. 20, and this is where speeding up the comparison task with an alignment-free method, like PCV, becomes very significant. Furthermore, PCV can outperform the ClustalW by offering hierarchical methods that require separation of distinct kinds of input sequences in the early phases.

## Conclusions

Due to the growing need for development of sequence comparison tools, especially protein sequences, in this work, we presented an alignment-free method that uses sequence information and physicochemical properties of amino acids. This method estimates the similarity of the protein sequence in whole by determining local similarity of fixed length blocks. As a result, although it is an alignment-free method, it can resemble the behavior of alignment-based methods for protein comparison. We compared this method, known as PCV, to some well-known alignment-based and alignment-free methods in a variety of ways. Specifically, we evaluated the PCV approach for 12 benchmark datasets considering various conditions, which is a superior of datasets compared to alternative studies. It should be mentioned that like other alignment-free methods, these datasets include classes with homologous sequences which may require a simple preprocessing search tool to select the homologous data. Assuming ClustalW^11^ as the reference method, in addition to improving the comparison speed compared to the other methods, Correlation Coefficient (CC) metric, RF distance, ROC curve, and the corresponding AUC metric indicate greater improvement for PCV method than the alternative alignment-free methods. Specifically, we reported an average CC of 94% between PCV and ClustalW methods, as well as more accurate classifications at the different levels of evolution. In this way, we can conclude that PCV is accurate and fast, while providing local similarity information which is not considered by other alignment-free methods. As the future works, PCV can be developed to display pseudo-dot plots and reduce the required amount of memory. Furthermore, due to its structure, employing repetitive operational units, PCV method can be implemented on widely available hardware platforms, such as FPGA, which can assist speeding up this approach, compared to its current CPU-based version. In addition, we would like to make PCV method available as a public online tool.

## Supplementary Information

Supplementary Information.

## Data Availability

The datasets generated and/or analysed during the current study are available in the “PCV-method” repository, https://github.com/SAkbari93/PCV-method.

## References

[1] Sun Z, Pei S, He RL, Yau SS-T (2020). A novel numerical representation for proteins: Three-dimensional chaos game representation and its extended natural vector. Comput. Struct. Biotechnol. J..

[2] Yu L, Zhang Y, Gutman I, Shi Y, Dehmer M (2017). Protein sequence comparison based on physicochemical properties and the position-feature energy matrix. Sci. Rep..

[3] Löchel HF, Eger D, Sperlea T, Heider D (2020). Deep learning on chaos game representation for proteins. Bioinformatics.

[4] Bateman A (2021). UniProt: The universal protein knowledgebase in 2021. Nucleic Acids Res..

[5] Saw AK, Tripathy BC, Nandi S (2019). Alignment-free similarity analysis for protein sequences based on fuzzy integral. Sci. Rep..

[6] Abnousi, A., Broschat, S. L. & Kalyanaraman, A. An alignment-free approach to cluster proteins using frequency of conserved k-mers. In *Proceedings of the 6th ACM Conference on Bioinformatics, Computational Biology and Health Informatics* 597–606 (2015).

[7] Abo-Elkhier MM, Abd Elwahaab MA, Abo El Maaty MI (2019). Measuring similarity among protein sequences using a new descriptor. Biomed. Res. Int..

[8] Xu C, Sun D, Liu S, Zhang Y (2016). Protein sequence analysis by incorporating modified chaos game and physicochemical properties into Chou’s general pseudo amino acid composition. J. Theor. Biol..

[9] Altschul SF, Gish W, Miller W, Myers EW, Lipman DJ (1990). Basic local alignment search tool. J. Mol. Biol..

[10] Pearson WR, Lipman DJ (1988). Improved tools for biological sequence comparison. Proc. Natl. Acad. Sci..

[11] Thompson JD, Higgins DG, Gibson TJ (1994). CLUSTAL W: Improving the sensitivity of progressive multiple sequence alignment through sequence weighting, position-specific gap penalties and weight matrix choice. Nucleic Acids Res..

[12] Edgar RC (2004). MUSCLE: A multiple sequence alignment method with reduced time and space complexity. BMC Bioinform..

[13] Katoh K, Misawa K, Kuma K, Miyata T (2002). MAFFT: A novel method for rapid multiple sequence alignment based on fast Fourier transform. Nucleic Acids Res..

[14] Altschul S (1997). Gapped BLAST and PSI-BLAST: A new generation of protein database search programs. Nucleic Acids Res..

[15] Eddy SR (1998). Profile hidden Markov models. Bioinformatics.

[16] Schwartz S (2003). Human–Mouse alignments with BLASTZ. Genome Res..

[17] Blanchette M (2004). Aligning multiple genomic sequences with the threaded blockset aligner. Genome Res..

[18] Remmert M (2011). Fast, scalable generation of high-quality protein multiple sequence alignments using Clustal Omega. Mol. Syst. Biol..

[19] Notredame C, Higgins DG, Heringa J (2000). T-coffee: A novel method for fast and accurate multiple sequence alignment. J. Mol. Biol..

[20] Sims GE, Jun S-R, Wu GA, Kim S-H (2009). Alignment-free genome comparison with feature frequency profiles (FFP) and optimal resolutions. Proc. Natl. Acad. Sci..

[21] Qi J, Luo H, Hao B (2004). CVTree: A phylogenetic tree reconstruction tool based on whole genomes. Nucleic Acids Res..

[22] Zuo G, Hao B (2015). CVTree3 web server for whole-genome-based and alignment-free prokaryotic phylogeny and taxonomy. Genomics Proteomics Bioinform..

[23] Vinga S, Almeida J (2003). Alignment-free sequence comparison—A review. Bioinformatics.

[24] Leimeister C-A, Morgenstern B (2014). kmacs: The k-mismatch average common substring approach to alignment-free sequence comparison. Bioinformatics.

[25] Davies M, Secker A, Freitas A, Timmis J, Clark E, Flower D (2008). Alignment-independent techniques for protein classification. Curr. Proteomics.

[26] Kawashima S, Pokarowski P, Pokarowska M, Kolinski A, Katayama T, Kanehisa M (2007). AAindex: Amino acid index database, progress report 2008. Nucleic Acids Res..

[27] Mu Z, Yu T, Qi E, Liu J, Li G (2019). DCGR: Feature extractions from protein sequences based on CGR via remodeling multiple information. BMC Bioinform..

[28] Hu H, Li Z, Dong H, Zhou T (2017). Graphical representation and similarity analysis of protein sequences based on fractal interpolation. IEEE/ACM Trans. Comput. Biol. Bioinform..

[29] Zhang Y, Wen J, Yau SST (2019). Phylogenetic analysis of protein sequences based on a novel k-mer natural vector method. Genomics.

[30] Bar-Joseph Z, Gifford DK, Jaakkola TS (2001). Fast optimal leaf ordering for hierarchical clustering. Bioinformatics.

[31] scipy.cluster.hierarchy.linkage tutorial. [Online]. https://docs.scipy.org/doc/scipy/reference/generated/scipy.cluster.hierarchy.linkage.html.

[32] Abd-Elwahaab MA, Abo Elkhier MM, Abo el Maaty MI (2019). A statistical similarity/dissimilarity analysis of protein sequences based on a novel group representative vector. Biomed. Res. Int..

[33] Liu Z, Meng J, Sun X (2008). A novel feature-based method for whole genome phylogenetic analysis without alignment: Application to HEV genotyping and subtyping. Biochem. Biophys. Res. Commun..

[34] Blaisdell BE (1989). Effectiveness of measures requiring and not requiring prior sequence alignment for estimating the dissimilarity of natural sequences. J. Mol. Evol..

[35] Sonego P, Kocsor A, Pongor S (2008). ROC analysis: Applications to the classification of biological sequences and 3D structures. Brief Bioinform..

[36] Sievers F, Higgins DG (2018). Clustal Omega for making accurate alignments of many protein sequences. Protein Sci..

