# Supplementary material for: An accurate alignment-free protein sequence comparator based on physicochemical properties of amino acids
Source: Sci Rep. 2022 Jul 1;12:11158. doi: 10.1038/s41598-022-15266-8 (PMC9247937; doi:10.1038/s41598-022-15266-8)
Supplement: Supplementary file 1 — Supplementary Information. [file 41598_2022_15266_MOESM1_ESM.docx]

An Accurate Alignment-free Protein Sequence Comparator Based on Physicochemical Properties of Amino Acids

**Saeedeh Akbari Ron Abadi^1^, Azam Sadat Abdosalehi^1^, Faezeh Pouyamehr^1^, Somayyeh Koohi^1,*^**

**^1^ Department of Computer Engineering, Sharif University of Technology, Tehran, Iran**

**^*^ Correspondence: E-mail: koohi@sharif.edu**

Contents

[AAindex1 clustering 2](#_Toc91447364)

[Block size and shift analysis 5](#_Toc91447365)

[Data 8](#_Toc91447366)

[ROC 13](#_Toc91447367)

[Runtime analysis 14](#_Toc91447368)

[Phylogenetic trees 18](#_Toc91447369)

[9 ND5 protein sequences 18](#_Toc91447370)

[8 ND6 protein sequences 20](#_Toc91447371)

[24 TF protein sequences 22](#_Toc91447372)

[24 Coronavirus protein sequences 24](#_Toc91447373)

[50 Coronavirus spike protein sequences 27](#_Toc91447374)

[27 Antifreeze protein sequences 31](#_Toc91447375)

[9 Betaglobin protein sequences 34](#_Toc91447376)

[50 Betaglobin protein sequences 36](#_Toc91447377)

[88 Betaglobin protein sequences 40](#_Toc91447378)

[20 Xylanase protein sequences 44](#_Toc91447379)

[113 Human rhinoviruses (HRV) and 3 HEV-C protein sequences 46](#_Toc91447380)

[1163 Influenza A viruses protein sequences 50](#_Toc91447381)

[References 54](#_Toc91447382)

# AAindex1 clustering

Amino acid physicochemical properties have been widely employed in bioinformatics research to demonstrate the characteristics of biological reactions. AAindex is a library of numerical indices that represent a wide range of amino acid physicochemical and biological properties. It was founded by Nakai et al. [1], who compiled a list of 222 amino acid indices and used hierarchical clustering techniques to elicit correlations between them. Following that, the AAindex database was updated. Specifically, the amino acid indices, the amino acid substitution matrices, and the amino acid contact potentials are listed in AAindex1, AAindex2, and AAindex3, respectively. It should be noted that in this work, we adopted AAindex1 with 566 features.

For categorizing these 566 features and reducing their volume, we first calculate the correlation between each pair of 566 properties and achieve a 566 × 566 matrix. Then, in a 566-dimensional space, we utilize this matrix to calculate a pairwise Euclidean distance and obtain a 1-d array. Afterwards, the linkage method is applied to this array to calculate the distance d(S, T) between two clusters S and T. Fig. S 1 depicts a summarized schematic of the aforementioned linkage procedure. The process starts with a forest of clusters that have yet to be utilized in the hierarchy being built, as shown in Algorithm S 1. When the forest's two clusters S and T are united to form a single cluster U, S and T are removed from the forest, and U is added. The procedure halts in a state that there is only one cluster left in the forest, and so, this cluster becomes the root. After each iteration, the program updates the distance matrix to reflect the distance between the newly generated cluster U and the existing ones in the forest. It should be noted that we adopt the entire linkage method which updates the distance matrix, as described in Eq. S 1. This is also known as the Farthest Point Algorithm or Voor Hees Algorithm [2].

| d(S,T)=max(dist(S[i],T[j]))  for all points i ∈S and j ∈T | Eq. S 1 |
| --- | --- |

Algorithm S 1 Utilized algorithm to cluster features of AAindex1[2]

| 1. Begin with the disjoint clustering having level $L\left( 0 \right)=0$ and sequence number $m=0$. 2. Find the most similar pair of clusters in the current clustering, say pair $\left( r \right),(s)$ according to $d[\left( r \right),\left( j \right)]$ where the minimum is over all pairs of clusters in the current clustering. 3. Increment the sequence number: $m=m+1$. Merge clusters $(r)$ and $(s)$ into a single cluster to form the next clustering $m$. Set the level of this clustering to $L\left( m \right)=d[\left( r \right),\left( s \right)]$. 4. Update the proximity matrix, $D$, by deleting the rows and columns corresponding to clusters $(r)$ and $(s)$ and adding a row and column corresponding to the newly formed cluster. The proximity between the new cluster, denoted $(r,s)$ and old custer $(k)$ is defined as $d\left[ \left( r \right),\left( s \right) \right]=max\{d\left[ \left( k \right),\left( r \right) \right],d\left[ \left( k \right),\left( s \right) \right]\}$. 5. If all objects are in one cluster, stop. Else, go to step 2. |
| --- |

A B C D E F

**Cluster U**

**Cluster S**

**Cluster T**

Fig. S 1 Simple dendrogram of complete linkage clustering algorithm

Finally, we create a flat set of clusters based on the hierarchical clustering approach using the given linkage matrix, assuming each flat cluster has a cophenetic distance of less than 0.2 × max distance (maximum Euclidean distance from the 1-d array). Following that, we determine the mean value of each amino acid considering all members within each of the 110 groups of properties obtained at the previous step. More details of this clustering approach are shown in Table S 1. As a key idea, to unify the impact of various physicochemical properties, these values are normalized using Studentized residual. As a result, a vector of 110 values is assigned to each amino acid. And finally, the extracted 110 × 20 physicochemical characteristics matrix is loaded as the algorithm's input file.

Table S 1 Details of assigning each property of AAindex1 to 110 clusters

| group 54 is: 12 13 15 59  group 55 is: 54 55 180 181 212 240 315 352 493 510 524  group 56 is: 2 78 108 132 358 399 444  group 57 is: 178 179 192 194 196 380 402 488 489  group 58 is: 310 473 480  group 59 is: 46 102 169 232 233  group 60 is: 552 553 555 563 565  group 61 is: 28 29 33 63 72 83 117 154 157 545 546 547 548 549 550 551 557 558 559 560 561  group 62 is: 31 483  group 63 is: 81 118 214 391  group 64 is: 91 261 262 263 410 416  group 65 is: 5 140 264 265 266 267 268 269 330 341 411 415  group 66 is: 256 328 332 363  group 67 is: 21 79 474  group 68 is: 359  group 69 is: 9 22 32 80 109 112 150 319 484 485 514 515  group 70 is: 96 159 361 481  group 71 is: 158 353 511  group 72 is: 116 177 304 445  group 73 is: 397 434 498  group 74 is: 284 364 378  group 75 is: 286  group 76 is: 23 452  group 77 is: 490 562  group 78 is: 84 475 476 477 478 479  group 79 is: 26 82 491  group 80 is: 318 360  group 81 is: 191 193 195 197 458 465 466 467  group 82 is: 133 201  group 83 is: 386 395 396  group 84 is: 75  group 85 is: 259 301 376  group 86 is: 564  group 87 is: 258 412 413 417  group 88 is: 442 443  group 89 is: 230 260  group 90 is: 98 229  group 91 is: 99  group 92 is: 42 329  group 93 is: 16 90 342 423 437  group 94 is: 270 331  group 95 is: 420  group 96 is: 19 38 60 97 100 119 138 160 163 171 186 223 224 231 253 307 339 366 414  group 97 is: 27 155 333 508  group 98 is: 303 362 392  group 99 is: 25 280  group 100 is: 52 306 349  group 101 is: 145 146 401  group 102 is: 274  group 103 is: 18 74 95  group 104 is: 311  group 105 is: 1 17  group 106 is: 373 379 486  group 107 is: 325 338 357 367 422  group 108 is: 501 503 504 506  group 109 is: 124 502  group 110 is: 403 408 | group 1 is: 87 127 129 213 252 355 356 381 428 492 522 543  group 2 is: 34 86 371 523  group 3 is: 89 326 327 409  group 4 is: 114 125 400  group 5 is: 70 88 94  group 6 is: 334 335 418  group 7 is: 106 288 291 292 431  group 8 is: 221 300 337  group 9 is: 436 453 499 500  group 10 is: 143 147 149 299 427 520  group 11 is: 439 461 462 464  group 12 is: 144 222 302  group 13 is: 111 115 153 388 455 519  group 14 is: 8 142 463  group 15 is: 14 113 148 182 183 215 239 297 298 390 425 426 438 521 537 538 539 540 541 542 544  group 16 is: 41 340  group 17 is: 176 294 507  group 18 is: 404 421  group 19 is: 251 405  group 20 is: 7 62 346 347 348 351  group 21 is: 24 37 40 47 53 104 107 121 162 165 227 228 236 255 289 290 375  group 22 is: 44 48 166 188 237 238 271 272 273 283 350  group 23 is: 281 287 312 454  group 24 is: 51 123 282 324  group 25 is: 43 126 174 235 308 336 372 419  group 26 is: 173 305 322 323 370 385  group 27 is: 30 92 406 407  group 28 is: 49 85 110  group 29 is: 313 429 430  group 30 is: 50 105 122 293 309 369 432 433  group 31 is: 377 556  group 32 is: 345 566  group 33 is: 93 295 424 509  group 34 is: 216 219 383  group 35 is: 156 217 250 316  group 36 is: 175 374 554  group 37 is: 249 435  group 38 is: 65 135  group 39 is: 134 189 440 441 457 459 469 470 472  group 40 is: 64 136 137 204 205 208 456 471  group 41 is: 190 202 203 207 460 516  group 42 is: 20 285 505  group 43 is: 296  group 44 is: 36 220 244 317 320 387 389 394 517 526  group 45 is: 199 200 206 209 468  group 46 is: 73 76  group 47 is: 68 69 71 77 198 314 525 527 528  group 48 is: 3 4 35 66 67 128 130 131 151 245 382 393 446 447 494 518  group 49 is: 6 10 11 56 57 58 170 184 185 210 211 241 242 243 246 247 248 321 354 365 384 448 449 450 451 487 495 496 497 512 513 529 530 531 532 533 534 535 536  group 50 is: 39 45 61 103 139 167 187 218 226 257 276 277 278 343 344  group 51 is: 101 141 161 164 168 225 234 254 275 279  group 52 is: 120 172 368  group 53 is: 152 398 482 |
| --- | --- |

# Block size and shift analysis

We examined five datasets, ND5, ND6, Coronavirus (24), Betaglobin (9), and Transferrins. For each dataset, we performed PCV with varied parameters to specify the best values of window size and maximum shift. Table S 2summarizes the RF distance for each dataset. According to this table, although the optimal parameter choices for various datasets are different, in general, the best PCV configuration can be specified by window size of 50 and maximum shift value of 2.

Table S 2 RF distance of PCV with several block sizes and maximum shift values

| **‌Block size** | **Maximum shift** | **ND5** | **Betaglobin** | **Coronaviruses** | **ND6** | **TF** |
| --- | --- | --- | --- | --- | --- | --- |
| 2 | 0 | 0 | 4 | 6 | 2 | 30 |
| 2 | 1 | 2 | 4 | 22 | 4 | 28 |
| 5 | 0 | 0 | 6 | 2 | 2 | 28 |
| 5 | 1 | 0 | 4 | 4 | 0 | 22 |
| 5 | 2 | 0 | 2 | 8 | 2 | 18 |
| 5 | 3 | 2 | 4 | 22 | 2 | 20 |
| 10 | 0 | 0 | 6 | 4 | 0 | 28 |
| 10 | 1 | 0 | 6 | 4 | 0 | 22 |
| 10 | 2 | 0 | 2 | 4 | 2 | 18 |
| 10 | 3 | 0 | 2 | 10 | 2 | 14 |
| 10 | 4 | 0 | 2 | 22 | 2 | 14 |
| 10 | 5 | 0 | 2 | 22 | 0 | 18 |
| 10 | 6 | 0 | 2 | 22 | 0 | 12 |
| 20 | 0 | 0 | 6 | 6 | 0 | 22 |
| 20 | 1 | 0 | 4 | 4 | 0 | 18 |
| 20 | 2 | 0 | 4 | 4 | 0 | 14 |
| 20 | 3 | 0 | 4 | 6 | 0 | 14 |
| 20 | 4 | 0 | 2 | 10 | 0 | 16 |
| 20 | 5 | 0 | 4 | 10 | 0 | 14 |
| 20 | 6 | 0 | 2 | 12 | 2 | 10 |
| 20 | 7 | 0 | 4 | 10 | 2 | 10 |
| 20 | 8 | 0 | 2 | 10 | 2 | 10 |
| 20 | 9 | 0 | 2 | 10 | 2 | 10 |
| 20 | 10 | 0 | 2 | 10 | 2 | 10 |
| 30 | 0 | 0 | 4 | 8 | 2 | 18 |
| 30 | 1 | 0 | 4 | 6 | 0 | 14 |
| 30 | 2 | 0 | 2 | 4 | 0 | 12 |
| 30 | 3 | 0 | 4 | 4 | 0 | 10 |
| 30 | 4 | 0 | 4 | 4 | 0 | 12 |
| 30 | 5 | 0 | 4 | 4 | 0 | 12 |
| 30 | 6 | 0 | 2 | 4 | 0 | 12 |
| 30 | 7 | 0 | 4 | 6 | 2 | 10 |
| 30 | 8 | 2 | 2 | 6 | 0 | 12 |
| 30 | 9 | 2 | 2 | 6 | 0 | 8 |
| 30 | 10 | 0 | 2 | 6 | 2 | 10 |
| 40 | 0 | 2 | 2 | 6 | 0 | 12 |
| 40 | 1 | 0 | 2 | 4 | 0 | 14 |
| 40 | 2 | 0 | 2 | 4 | 0 | 12 |
| 40 | 3 | 0 | 2 | 6 | 0 | 10 |
| 40 | 4 | 0 | 2 | 10 | 0 | 12 |
| 40 | 5 | 0 | 2 | 10 | 0 | 12 |
| 40 | 6 | 0 | 2 | 10 | 0 | 10 |
| 40 | 7 | 0 | 2 | 10 | 0 | 8 |
| 40 | 8 | 0 | 2 | 10 | 0 | 8 |
| 40 | 9 | 0 | 2 | 10 | 0 | 8 |
| 40 | 10 | 0 | 2 | 10 | 0 | 10 |
| 50 | 0 | 2 | 4 | 0 | 0 | 8 |
| 50 | 1 | 2 | 4 | 4 | 0 | 14 |
| 50 | 2 | 2 | 2 | 4 | 0 | 10 |
| 50 | 3 | 0 | 2 | 4 | 0 | 10 |
| 50 | 4 | 0 | 2 | 4 | 0 | 10 |
| 50 | 5 | 0 | 2 | 4 | 0 | 8 |
| 50 | 6 | 0 | 2 | 4 | 0 | 8 |
| 50 | 7 | 2 | 2 | 6 | 0 | 8 |
| 50 | 8 | 0 | 2 | 8 | 0 | 6 |
| 50 | 9 | 0 | 2 | 6 | 0 | 10 |
| 50 | 10 | 0 | 2 | 6 | 0 | 10 |
| 50 | 11 | 0 | 2 | 6 | 0 | 10 |
| 50 | 12 | 0 | 2 | 8 | 0 | 10 |
| 60 | 1 | 0 | 4 | 4 | 2 | 14 |
| 60 | 2 | 0 | 4 | 4 | 2 | 10 |
| 60 | 3 | 0 | 4 | 4 | 2 | 12 |
| 60 | 4 | 0 | 4 | 8 | 2 | 12 |
| 60 | 5 | 0 | 4 | 4 | 2 | 12 |
| 60 | 6 | 0 | 4 | 4 | 2 | 10 |
| 60 | 7 | 0 | 4 | 6 | 2 | 10 |
| 60 | 8 | 0 | 4 | 6 | 2 | 8 |
| 60 | 9 | 0 | 4 | 4 | 2 | 6 |
| 60 | 10 | 0 | 4 | 4 | 2 | 6 |
| 60 | 1 | 0 | 4 | 4 | 2 | 6 |
| 70 | 0 | 0 | 4 | 4 | 4 | 12 |
| 70 | 1 | 2 | 4 | 4 | 2 | 12 |
| 70 | 2 | 0 | 2 | 4 | 2 | 14 |
| 70 | 3 | 2 | 2 | 4 | 2 | 14 |
| 70 | 4 | 2 | 2 | 4 | 2 | 12 |
| 70 | 5 | 0 | 2 | 4 | 2 | 12 |
| 70 | 6 | 2 | 2 | 4 | 2 | 10 |
| 70 | 7 | 0 | 2 | 6 | 2 | 10 |
| 70 | 8 | 2 | 2 | 8 | 2 | 10 |
| 70 | 9 | 0 | 2 | 8 | 2 | 8 |
| 70 | 10 | 0 | 2 | 8 | 2 | 10 |
| 80 | 0 | 0 | 2 | 4 | 0 | 14 |
| 80 | 1 | 2 | 2 | 4 | 0 | 12 |
| 80 | 2 | 0 | 2 | 6 | 0 | 12 |
| 80 | 3 | 0 | 2 | 8 | 0 | 12 |
| 80 | 4 | 0 | 2 | 10 | 0 | 12 |
| 80 | 5 | 0 | 2 | 10 | 0 | 12 |
| 80 | 6 | 0 | 2 | 10 | 2 | 8 |
| 80 | 7 | 0 | 2 | 10 | 2 | 8 |
| 80 | 8 | 0 | 2 | 10 | 2 | 8 |
| 80 | 9 | 0 | 2 | 10 | 2 | 8 |
| 80 | 10 | 0 | 2 | 10 | 2 | 6 |
| 90 | 0 | 2 | 2 | 4 | 2 | 16 |
| 90 | 1 | 2 | 2 | 4 | 2 | 16 |
| 90 | 2 | 2 | 2 | 4 | 2 | 14 |
| 90 | 3 | 0 | 2 | 4 | 2 | 20 |
| 90 | 4 | 2 | 2 | 4 | 2 | 16 |
| 90 | 5 | 2 | 2 | 4 | 2 | 12 |
| 90 | 6 | 2 | 4 | 4 | 0 | 14 |
| 90 | 7 | 2 | 4 | 4 | 2 | 14 |
| 90 | 8 | 4 | 4 | 4 | 2 | 14 |
| 90 | 9 | 2 | 2 | 4 | 2 | 12 |
| 90 | 10 | 2 | 2 | 4 | 2 | 12 |
| 100 | 0 | 2 | 4 | 4 | 2 | 14 |
| 100 | 1 | 2 | 4 | 4 | 2 | 14 |
| 100 | 2 | 2 | 4 | 4 | 2 | 12 |
| 100 | 3 | 0 | 4 | 4 | 2 | 14 |
| 100 | 4 | 0 | 2 | 4 | 2 | 12 |
| 100 | 5 | 0 | 4 | 4 | 2 | 10 |
| 100 | 6 | 0 | 2 | 4 | 2 | 10 |
| 100 | 7 | 0 | 2 | 4 | 2 | 10 |
| 100 | 8 | 0 | 4 | 4 | 2 | 8 |
| 100 | 9 | 0 | 4 | 6 | 2 | 10 |
| 100 | 10 | 0 | 4 | 4 | 2 | 10 |

# Data

We used 12 benchmark datasets, as available at [github](https://github.com/SAkbari93/PCV-method.git), while access IDs of ND5, ND6, TF, Coronavirus (50), and Betaglobin (50) are listed in Table S 3 to Table S 7, respectively.

Table S 3 The concise information for 9 ND5 protein sequences [3]


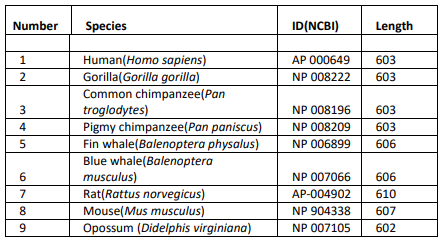


Table S 4 : The concise information for 8 ND6 protein sequences [3]


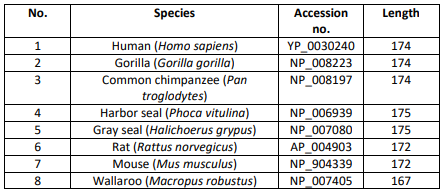


Table S 5 The concise information for 24 TF protein sequences [3]


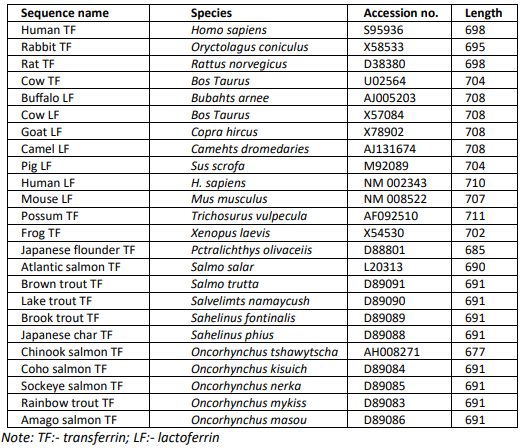


Table S 6 The 24 coronaviruses protein sequences [4]

| Abbreviation | Access no. | Length | Class |
| --- | --- | --- | --- |
| TGEVG | CAB91145 | 1447 | I |
| TGEV | NP058424 | 1447 | I |
| PEDVC | AAK38656 | 1383 | I |
| PEDV | NP598310 | 1383 | I |
| HCoVOC43 | NP937950 | 1361 | II |
| BCoVE | AAK83356 | 1363 | II |
| BCoVL | AAL57308 | 1363 | II |
| BCoVM | AAA66399 | 1363 | II |
| BCoVQ | AAL40400 | 1363 | II |
| IBVC | AAS00080 | 1169 | III |
| IBV | NP 040831 | 1162 | III |
| GD03T0013 | AAS10463 | 1255 | SARS-CoV |
| PC4127 | AAU93318 | 1255 | SARS-CoV |
| PC4137 | AAV49720 | 1255 | SARS-CoV |
| PC4205 | AAU93319 | 1255 | SARS-CoV |
| civet007 | AAU04646 | 1255 | SARS-CoV |
| civet010 | AAU04649 | 1255 | SARS-CoV |
| A022 | AAV91631 | 1255 | SARS-CoV |
| GD01 | AAP51227 | 1255 | SARS-CoV |
| GZ02 | AAS00003 | 1255 | SARS-CoV |
| BJ01 | AAP30030 | 1255 | SARS-CoV |
| FRA | AAP50485 | 1255 | SARS-CoV |
| TOR2 | AAP41037 | 1255 | SARS-CoV |
| TaiwanTC1 | AAQ01597 | 1255 | SARS-CoV |

Table S 7 The concise information of 50 coronavirus spike protein [3]


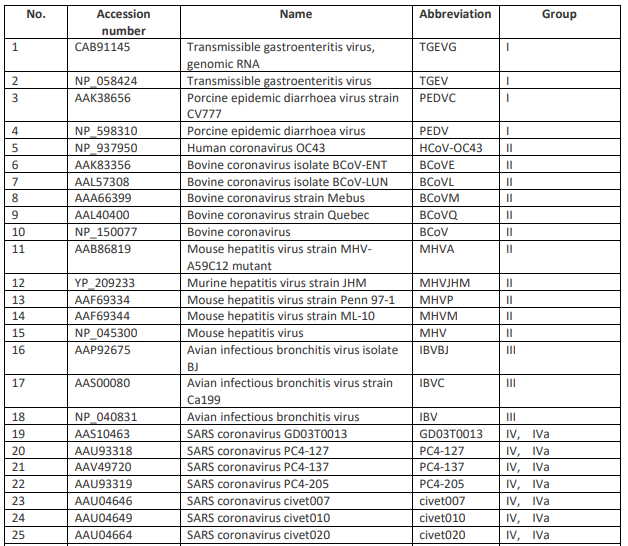


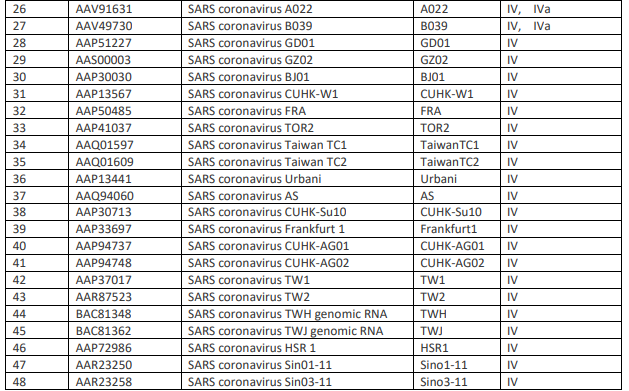


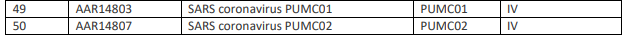


Table S 8 The concise information of 50 betaglobin protein sequences [3]


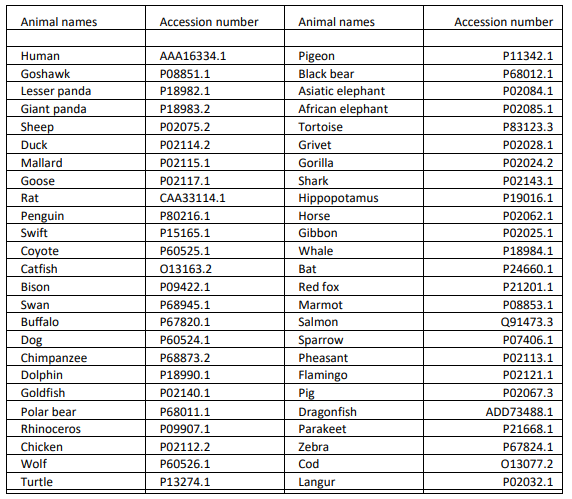


# ROC

Statistical measurements, like AUC (area under the ROC) values obtained from ROC (Receiver operating characteristic) curves, can be used to assess the effectiveness of comparison and clustering methods. Rather than comparing models, AUC values are frequently employed to describe methods’ accuracy. Table S 8 shows the accuracy categorization of AUC and the associated interpretation. A small AUC value does not necessarily mean that a technique provides a poor model; rather, it merely indicates that additional factors, in addition to the acknowledged predictors, affect the response variable [3].

For ROC analysis, a similarity matrix is employed. Moreover, a clustering matrix, with the same dimension as the similarity matrix, is required, which contains entities of 0 or 1; where, an entry equals 1 if two sequences belong to the same class (i.e., family level classification, phylum level classification, genus level classification, and etc.), and 0 otherwise 0. The ROC curve is created by adjusting the decision threshold between the minimum and maximum values of the similarity matrix and plotting the FPR (false positive rate) on the X-axis and the TPR (true positive rate) on the Y-axis. A discrete classifier may be obtained by using a specific decision threshold at each point on the ROC curve [3][5]. The area under the ROC curve (AUC) is calculated as an assessment statistic, once the ROC curve is plotted.

Table S 9 AUC classification accuracy [3]

| AUC Range | Classification |
| --- | --- |
| AUC ≥ 0.9 | high accuracy |
| 0.7 ≤ AUC< 0.9 | moderate accuracy |
| 0.5 ≤ AUC< 0.7 | Low accuracy |

To further evaluate our method, in addition to generating ROC curves for PCV and ClustalW as the reference method, we used the curves published in [3] for the fuzzy integral based method and five alternative alignment-free methods, i.e. FFP, RTD, CV, NCD, and BBC. On the other hand, as stated before, two similarity matrices and a classification matrix are utilized to construct the ROC curve. However, for each dataset, depending on the evolutionary level, different classification matrices are used for different assessments. It should be noted that for some datasets (i.e. ND5, ND6, and TF), there is no clear categorization, and alternative classification matrices have been reported in different studies. As a result, considering the distance matrix provided at [3] by the fuzzy integral based method, we constructed its ROC curves using our classification matrix. In this manner, we achieved two ROC curves for the fuzzy integral approach (the one published at [3] and the other one we generated from the provided matrix). It should be noted that the classification matrix reported at [3] is still used for the other five alignment-free methods. Given the ROC curves and the repetition of the ROCs production for the fuzzy integral based method and their AUC values, we can conclude that our classification matrices are more rigid and reliable, and hence, it is possible that the computed AUC values based on our classification matrix for five alternative alignment-free methods be lower than the corresponding reported values at [3].

# Runtime analysis

Since PCV is implemented with a non-optimal code in MATLAB, its execution time is far more than its minimum runtime. Therefore, in this section, we provide the runtime estimation for its optimal implementation. Memory access times, for both memory read and write operations, are also considered for an accurate runtime estimation. MATLAB code implementing PCV (Algorithm S 2), its runtime formulation and the corresponding parameters (Eq. S 2), as well as the required clock time for each instruction (Table S 9) are shown as follows. Red part of Eq. S 2 represents runtime estimation for load and write of data and green part represents runtime estimation for encoding and comparison blocks of PCV which can be executed in parallel. It should be noted in these equations, N is the number of Sequences, L is the maximum length of sequences, nb is the number of blocks for each sequence which is equal to L/(block size) with block size = 50, nsh equals 2 ×d+1 with d as the maximum shift value, corenum is the number of CPU cores, and finally, Sum, iadd,isub, and, or, shift, rotate, load, store, imul, fadd, fmul, fdiv, fsqrt, read, write, and compare present the clock time of instructions divided by CPU’s clock frequency.

| $T_{\mathrm{PCV}}= \left( N\times L\times read \right)+\left( N\times write\times\left( \frac{L}{\mathrm{nb}} \right) \right)+\left( 20\times110\times load \right)+$  $\left( \left( nsh\times shift \right)+\left( N\times nsh\times nb\times compare \right)+\left( N\times nsh\times110\times nb\times sum \right)+\left( N\times nsh\times110\times nb\times\left( \left( 20\times nb\times compare \right)+\left( L\times sum \right)+\left( L\times sum \right)+\left( L\times div \right)+\left( L\times mul \right)+\left( L\times\left( div+sum+sub+power+mul \right) \right) \right) \right)+\left( N\times nsh\times nb\times4\times110\times\left( mean+std+sub+div \right) \right)+\left( N\times nsh\times write \right)+\left( N\times N*\left( \frac{N-1}{2} \right)\times nb\times nsh\times\left( power+sub+sum+root \right) \right)+\left( N\times\left( \frac{N-1}{2} \right)\times nb\times min \right)+\left( N\times\left( \frac{N-1}{2} \right)\times sum \right) \right)/corenum$ | Eq. S 2 |
| --- | --- |

Algorithm S 2 PCV MATLAB code

| clc  clear  %% Read files  name='Coronavirus';    filename=strcat(name,'.fasta');  [IDs, U] = fastaread(filename);  %% Read PCH  load('Avgdata.mat')  DD = Avgdata; %add abs for positive  DD(isnan(Avgdata))=0;  %% mean and standard devision  M=((sum(DD,2))/20)';  S=(std(DD,0,2))';  for i=1:size(DD,1)  norm_data(i,:) = (DD(i,:) - M(i))/ S(i);  end  %%  Pattern= {'A' 'R' 'N' 'D' 'C' 'Q' 'E' 'G' 'H' 'I' 'L' 'K' 'M' 'F' 'P' 'S' 'T' 'W' 'Y' 'V'};  P = struct();  for k=1:size(norm_data,2)  p = Pattern{1,k};  y = norm_data(1:end,k)';  P.(p) = y;  end  P.('X') = [0];  %% Block generation1  L = zeros(1,length(U));  for k=1:length(U)  L(k) = length(U{k});  end  Lmax = max(L);  Lm =60;  Lshift =15;  Le = Lm*fix(Lmax/Lm + 1);  for k=1:length(U)  U{k}(L(k)+1:Le) = 'X';  end  numBlk = fix(Le/Lm);  %% Block generation2  Y = struct();    for n=1:length(U)  BB= [];  for k=-Lshift:Lshift  str = U{n}';str(end+1:end+abs(k))='X';  strs = circshift(str,k)';strs(end-abs(k)+1:end)=[];  Y(n).Data(k+Lshift+1).Mat = reshape(strs,Lm,[])';  for i = 1:size(Y(n).Data(k+Lshift+1).Mat,1)  for j=1:size(Y(n).Data(k+Lshift+1).Mat,2)  V = [];  strkey = Y(n).Data(k+Lshift+1).Mat(i,j);  if isfield(P,strkey)  v(j,:,i) = P.(strkey);  for m=1:i  W(m,:) =sum(v(:,:,m),1);  end  Y(n).Count(k+Lshift+1).Data(m,:)= W(m,:);  end  end  end  end  end    %% Moment  load('PatternG.mat');  C = struct();  for n=1:length(U)  CC= [];  for k=-Lshift:Lshift  str = U{n}';str(end+1:end+abs(k))='X';  strs = circshift(str,k)';strs(end-abs(k)+1:end)=[];  C(n).Data(k+Lshift+1).Mat = reshape(strs,Lm,[])';  for c = 1:110  for i = 1:size(C(n).Data(k+Lshift+1).Mat,1)  C(n).Count(k+Lshift+1).Data(c,:,i) = moment(C(n).Data(k+Lshift+1).Mat(i,:),PatternG{c});  end  end      for i=1:size(C(n).Count,2)  for j=1:numBlk  C(n).mom(i).Data(:,:,:)=permute(C(n).Count(i).Data,[2,1,3]);  C(n).non(i).Data(j,:)=reshape(C(n).mom(i).Data(:,:,j),1,440);  end  end  end    %Normalization    for i=1:size(C(n).Count,2)  M=((sum(C(n).non(i).Data,2))/440)';  S=(std(C(n).non(i).Data,0,2))';  for j=1:numBlk  norm_data_mom(j,:) = (C(n).non(i).Data(j,:) - M(j))/ S(j);  end  C(n).son(i).Data = norm_data_mom;  C(n).son(i).Data(isnan( C(n).son(i).Data))=0;  end    end      %% Merge  for n=1:length(U)  for i=1:size(C(n).Count,2)  F(n).Count(i).Data=[Y(n).Count(i).Data,C(n).son(i).Data];  end  end  %% Euclidean distance  [a0,b0]=size(F);  temp0=F(1).Count;  [a1,b1]=size(temp0);  temp1=temp0(1).Data;  [a2,b2]=size(temp1);    out3=zeros(b0);  out2=zeros(a2,1);  out1=zeros(b1);    for u=1:b0  for v=1:b0  for p=1:a2  for i=1:b1  for j=1:b1  G1=F(u).Count(i).Data(p,:);  G2=F(v).Count(j).Data(p,:);  D= max(abs(bsxfun(@minus,G1,G2)),[],2);  out1(i,j)=D;  end  end  out2(p)=min(min(out1));  end  out3(u,v)=sum(out2);  end  end  %% Mega format  outputfilename=strcat('PCHvector_Block-',name,'.meg'); %Change Name of File  num_of_seq=length(U);  outfile=fopen(outputfilename, 'w');  fprintf(outfile, '#mega\n');  fprintf(outfile, '!Title: TEST;\n');  fprintf(outfile, '!Format DataType=Distance DataFormat=LowerLeft NTaxa=%d;\n', num_of_seq);  fprintf(outfile, '\n');  for k = 1 : num_of_seq  fprintf(outfile, '[%d] #%s\n', k,IDs{k});  end  fprintf(outfile, '\n');  for j = 2 : num_of_seq  fprintf(outfile, '[%d] ', j);  for k = 1 : (j-1)  fprintf(outfile, ' %8f', out3(j, k));  end  fprintf(outfile, '\n');  end  fprintf(outfile, '\n');  fclose(outfile); |
| --- |

Table S 10 clock time of instructions

| Function | Number of clocks |
| --- | --- |
| Sum | 3 |
| iadd/isub | 1 |
| and, or, etc. | 1 |
| shift, rotate | 1 |
| load/store | 1-2 |
| imul | 3-15 |
| fadd | 3 |
| fmul | 3 |
| fdiv | 15-25 |
| fsqrt | 15-25 |
| read/write | 1-2 |
| compare | 1 |

# Phylogenetic trees

## 9 ND5 protein sequences


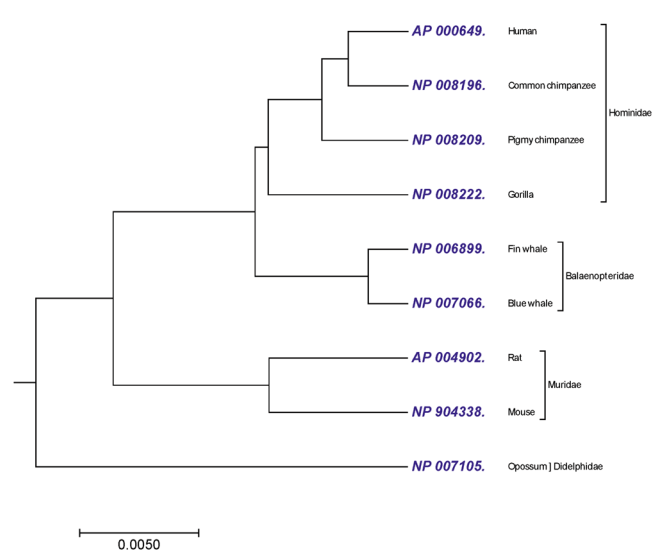


Fig. S 2 The phylogenetic tree of 9 sequences of NADH Dehydrogenase 5 protein constructed by fuzzy integral based method [3]


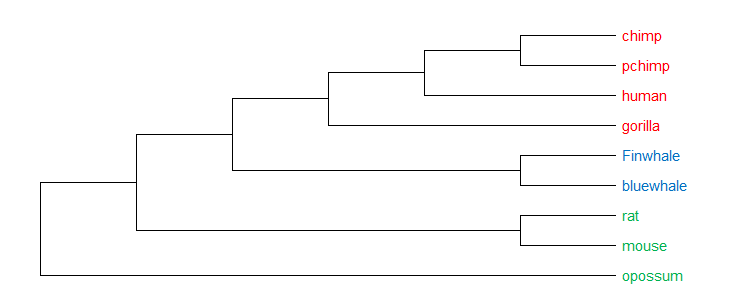


Fig. S 3 The phylogenetic tree of 9 sequences of NADH Dehydrogenase 5 protein constructed by ClustalW (NJ)


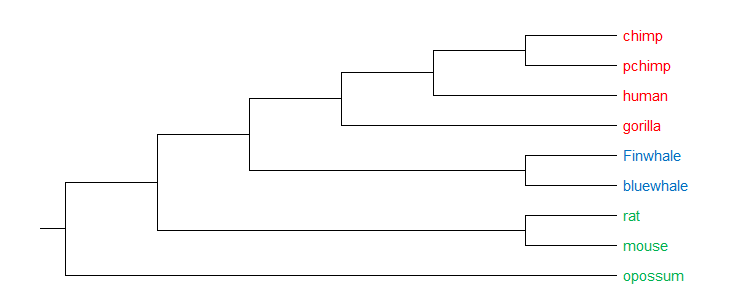


Fig. S 4 The phylogenetic tree of 9 sequences of NADH Dehydrogenase 5 protein constructed by ClustalW (UPGMA)


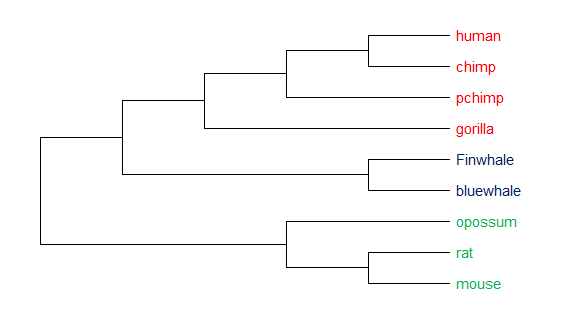


Fig. S 5 The phylogenetic tree of 9 sequences of NADH Dehydrogenase 5 protein constructed by PCV (NJ)

## 8 ND6 protein sequences


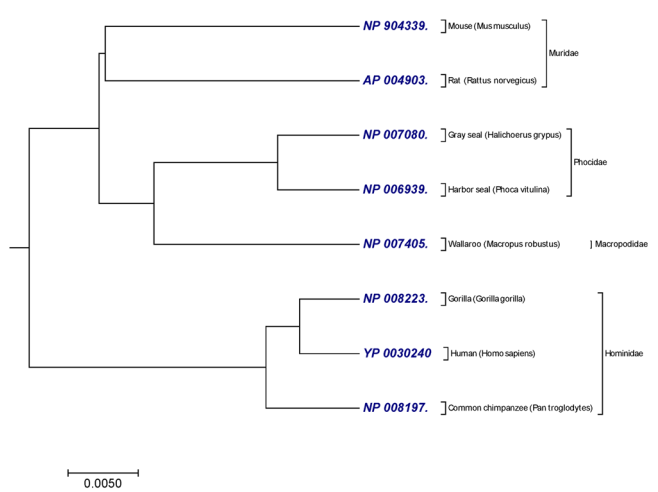


Fig. S 6 The phylogenetic tree of 8 sequences of NADH Dehydrogenase 6 protein constructed by fuzzy integral based method [3]


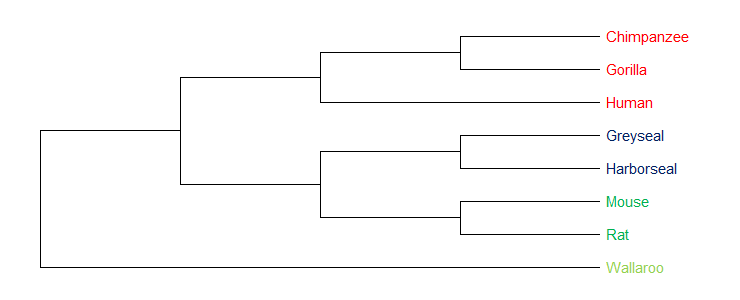


Fig. S 7 The phylogenetic tree of 8 sequences of NADH Dehydrogenase 6 protein constructed by ClustalW (NJ)


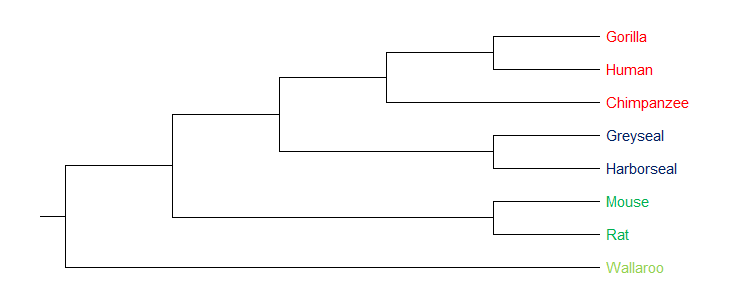


Fig. S 8 The phylogenetic tree of 8 sequences of NADH Dehydrogenase 6 protein constructed by ClsutalW (UPGMA)


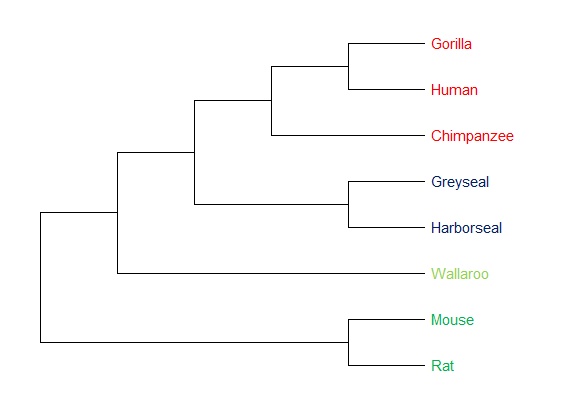


Fig. S 9 The phylogenetic tree of 8 sequences of NADH Dehydrogenase 6 protein constructed by PCV (NJ)

## 24 TF protein sequences


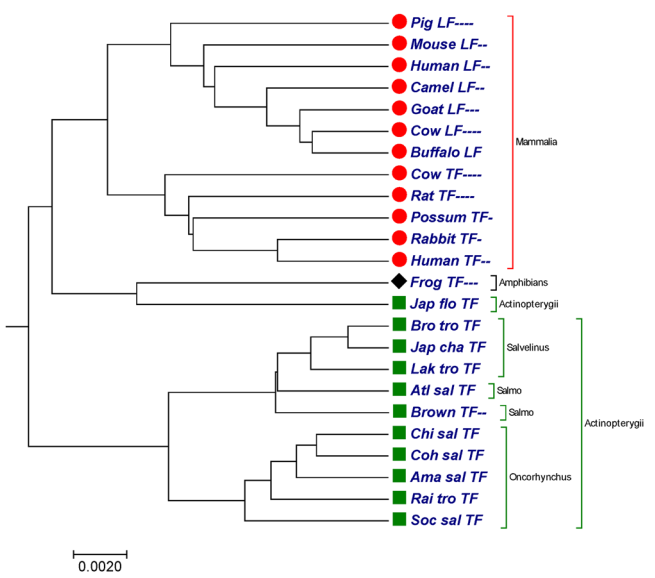


Fig. S 10 The phylogenetic tree of 24 sequences of transferrins protein constructed by fuzzy integral based method [3]


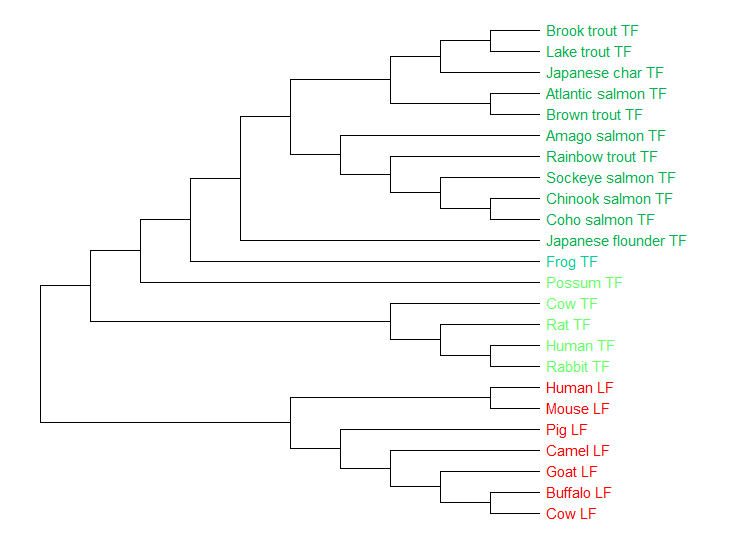


Fig. S 11 The phylogenetic tree of 24 sequences of transferrins protein constructed by ClsutalW (NJ)


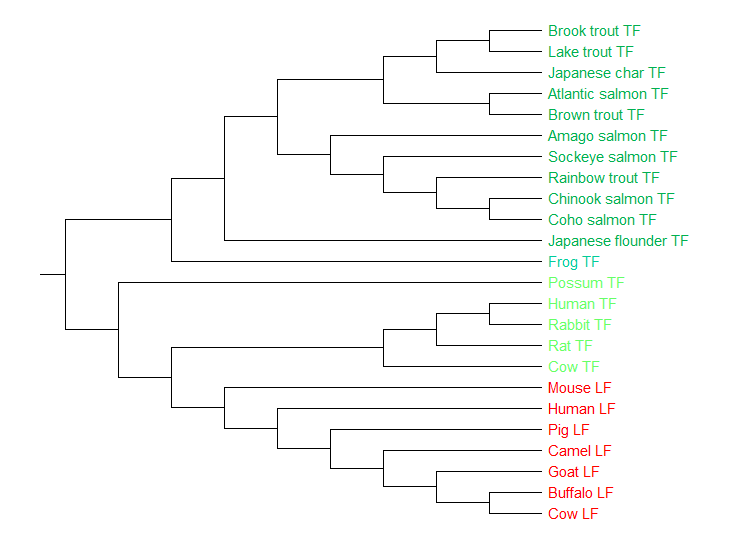


Fig. S 12 The phylogenetic tree of 24 sequences of transferrins protein constructed by ClustalW (UPGMA)


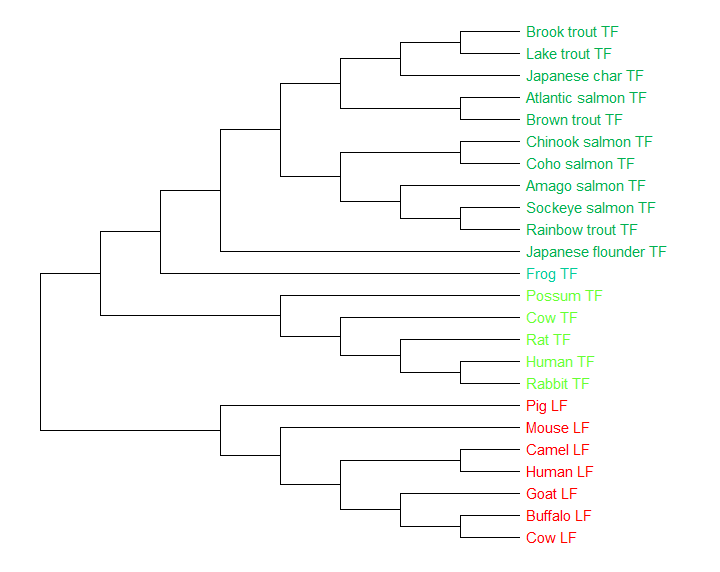


Fig. S 13 The phylogenetic tree of 24 sequences of transferrins protein constructed by PCV (NJ)

## 24 Coronavirus protein sequences

Table S 11 Mean and standard deviation descriptor of the coronaviruses protein sequences reported by intensity method [4]

| Abb. | Class no. | Mean | Standard deviation |
| --- | --- | --- | --- |
| TGEVG | I | 38.643 | 10.9412 |
| TGEV | I | 38.643 | 10.9412 |
| PEDVC | I | 38.452 | 11.1723 |
| PEDV | I | 38.452 | 11.1723 |
| HCoVOC43 | II | 38.703 | 10.7564 |
| BCoVE | II | 38.668 | 10.6803 |
| BCoVL | II | 38.678 | 10.6846 |
| BCoVM | II | 38.698 | 10.7755 |
| BCoVQ | II | 38.714 | 10.7656 |
| IBVC | III | 38.601 | 10.6271 |
| IBV | III | 38.654 | 10.6458 |
| GD03T0013 | SARS-CoV | 38.833 | 10.5783 |
| PC4127 | SARS-CoV | 38.838 | 10.5744 |
| PC4137 | SARS-CoV | 38.832 | 10.5785 |
| PC4205 | SARS-CoV | 38.838 | 10.5733 |
| Civet007 | SARS-CoV | 38.831 | 10.587 |
| Civet010 | SARS-CoV | 38.833 | 10.5829 |
| A022 | SARS-CoV | 38.829 | 10.5892 |
| GD01 | SARS-CoV | 38.821 | 10.5946 |
| GZ02 | SARS-CoV | 38.824 | 10.5867 |
| BJ01 | SARS-CoV | 38.816 | 10.5912 |
| FRA | SARS-CoV | 38.8189 | 10.5875 |
| TOR2 | SARS-CoV | 38.8186 | 10.5932 |
| TaiwanTC1 | SARS-CoV | 38.8176 | 10.5928 |


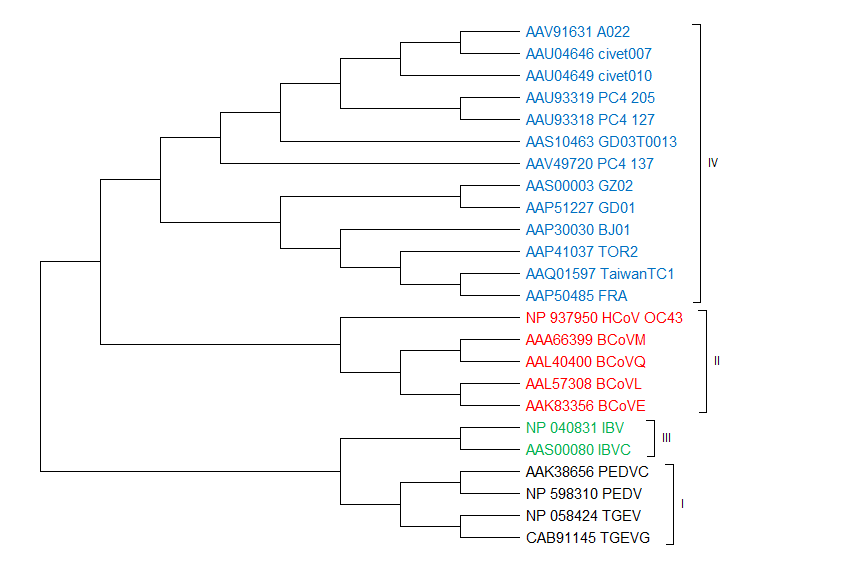


Fig. S 14 24 Coronavirus protein sequences constructed by Clustalw (NJ)


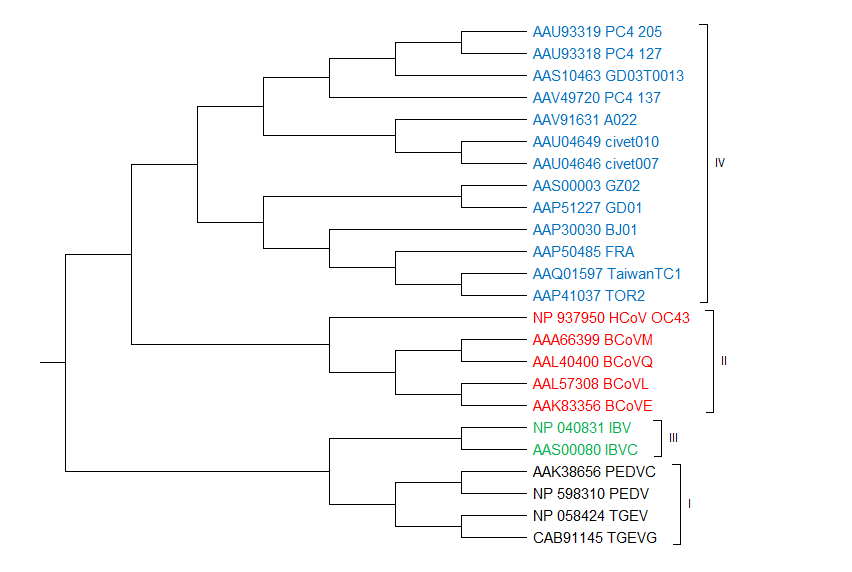


Fig. S 15 24 Coronavirus protein sequences constructed by ClustalW (UPGMA)


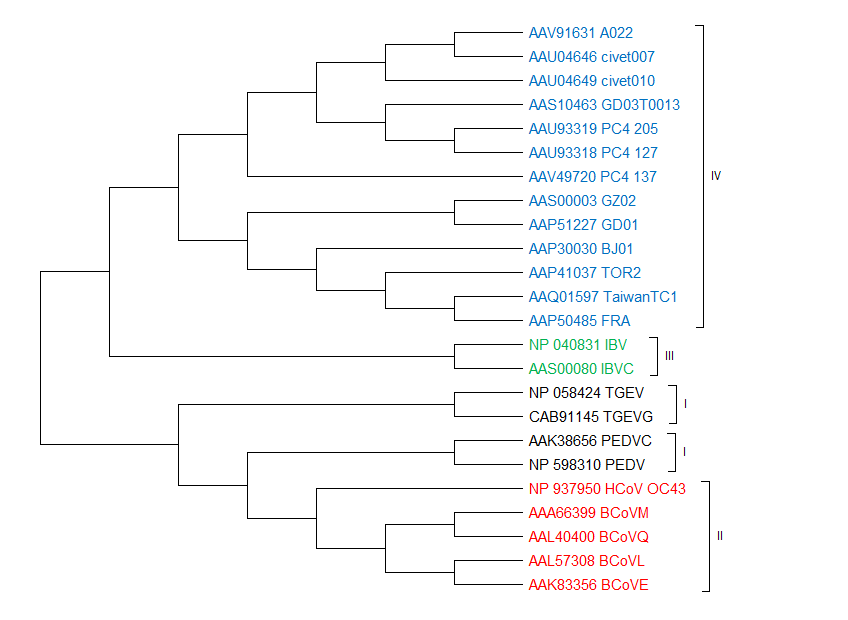


Fig. S 16 24 Coronavirus protein sequences constructed by PCV (NJ)

## 50 Coronavirus spike protein sequences


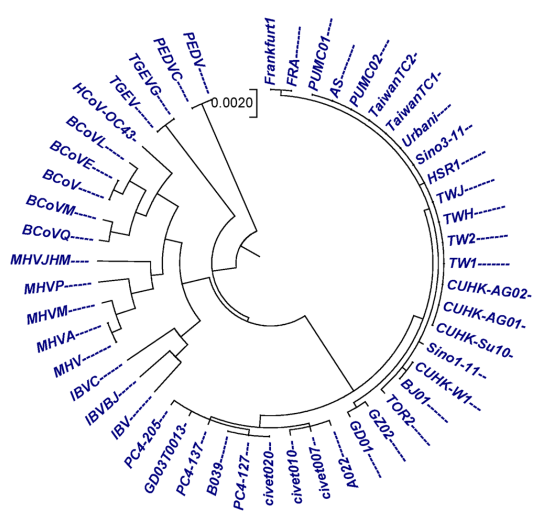


Fig. S 17 50 Coronavirus protein sequences constructed by fuzzy integral based method [3]


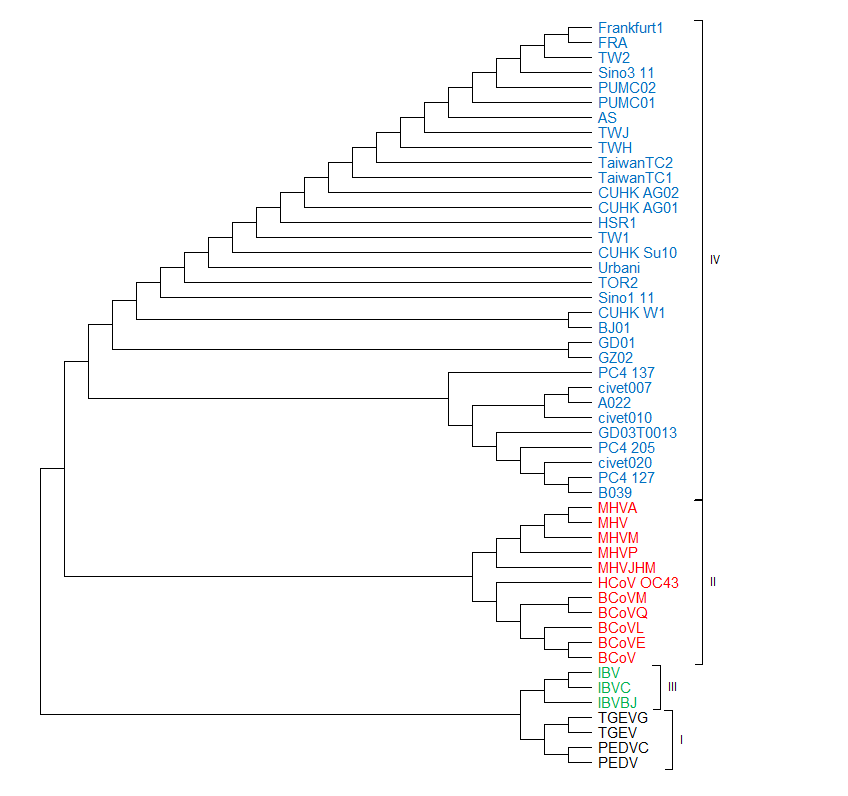


Fig. S 18 50 Coronavirus protein sequences constructed by ClustalW (NJ)


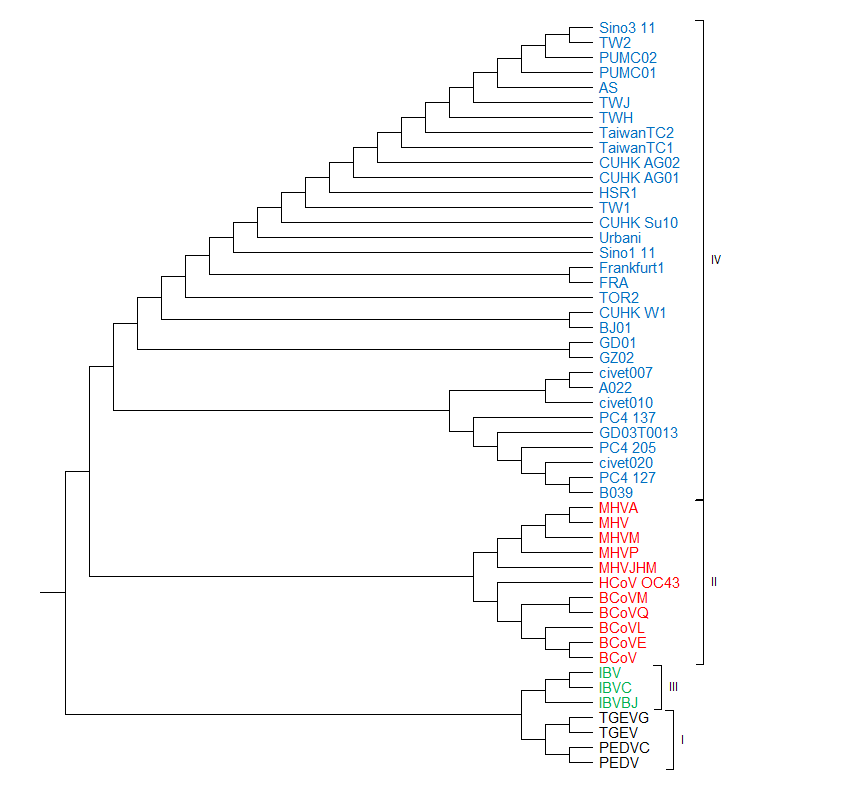


Fig. S 19 50 Coronavirus protein sequences constructed by ClsutalW (UPGMA)


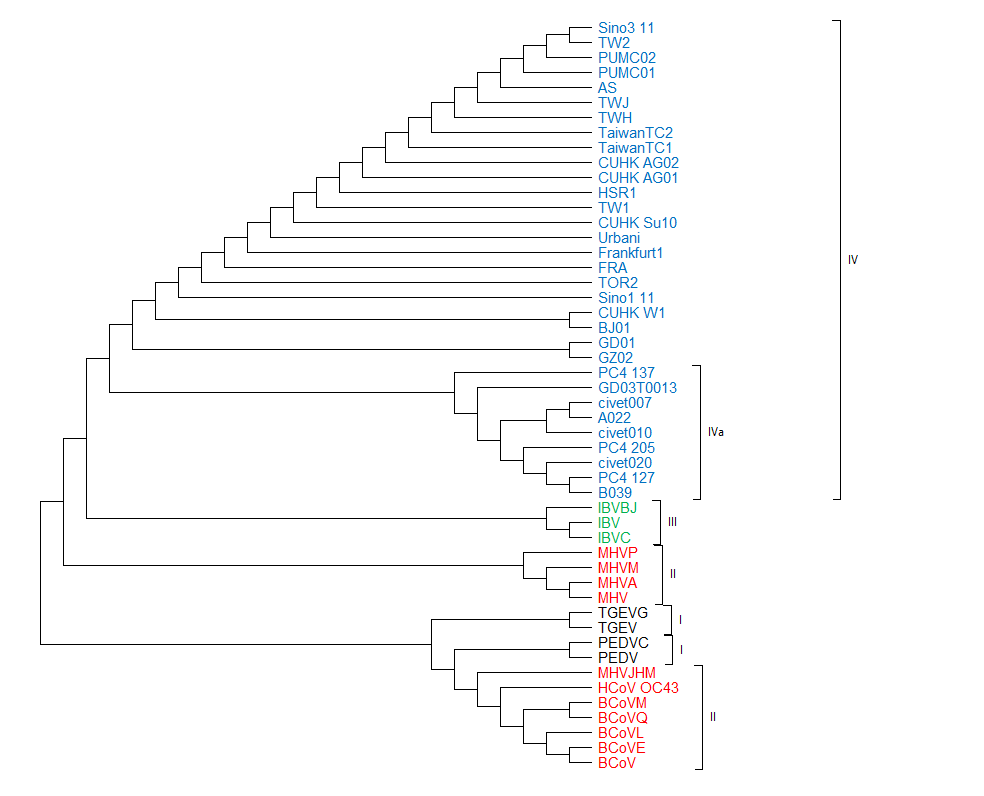


Fig. S 20 50 Coronavirus protein sequences constructed by PCV (NJ)

## 27 Antifreeze protein sequences


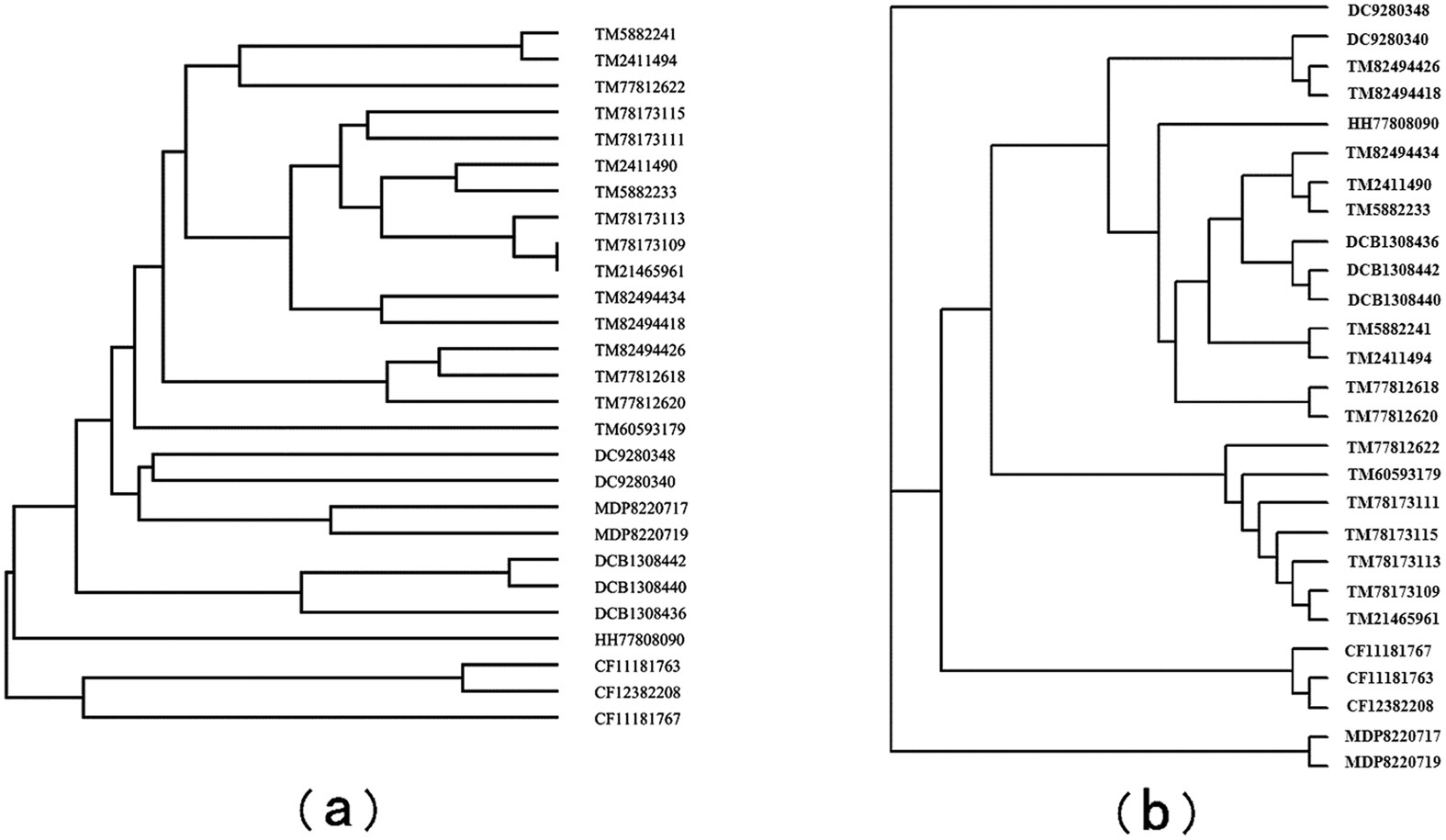


Fig. S 21 27 Antifreeze proteins (AFPs) sequences constructed by Energy matrix method [6]


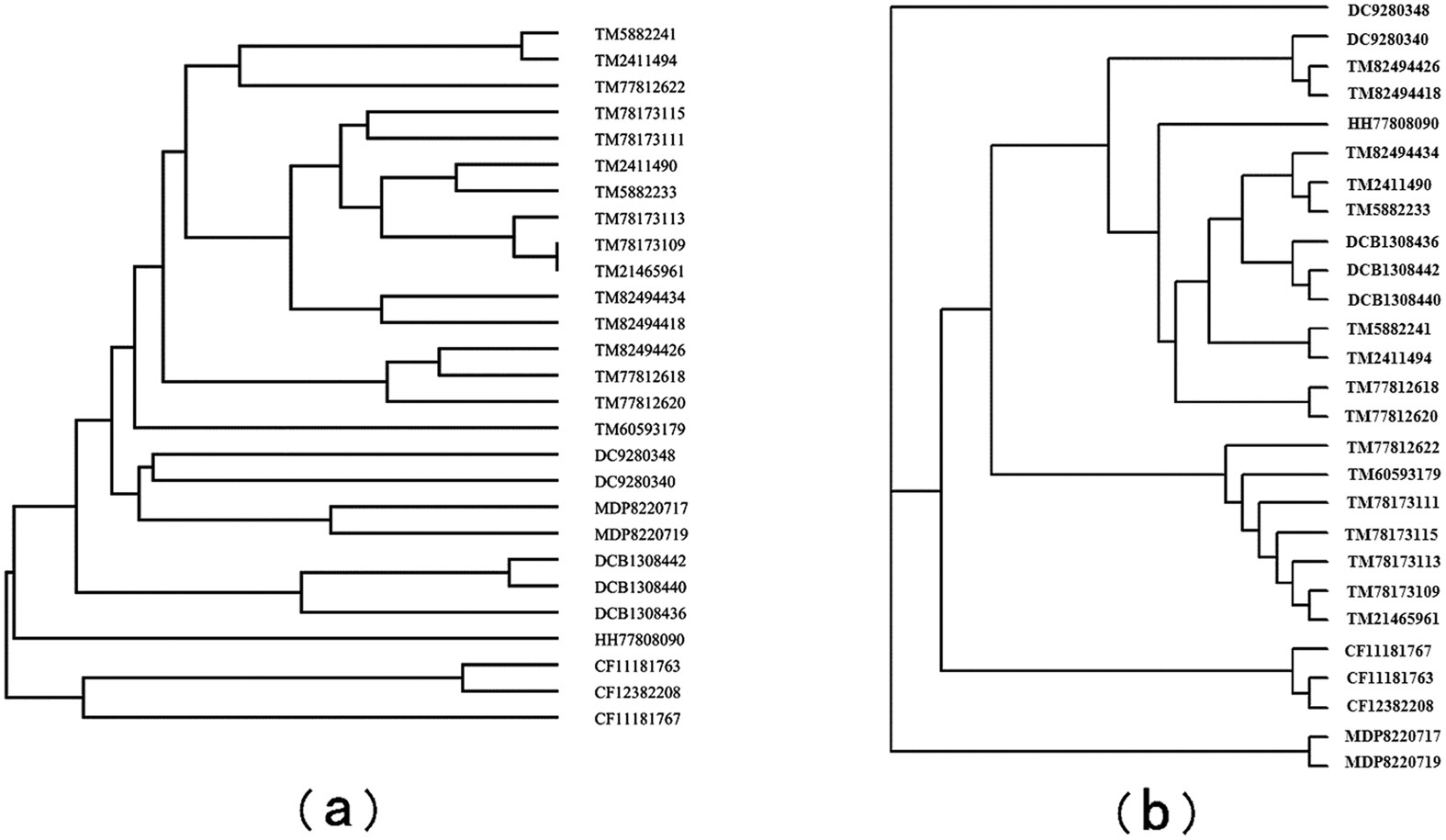


Fig. S 22 27 Antifreeze proteins (AFPs) sequences constructed by ClsutalW [6]


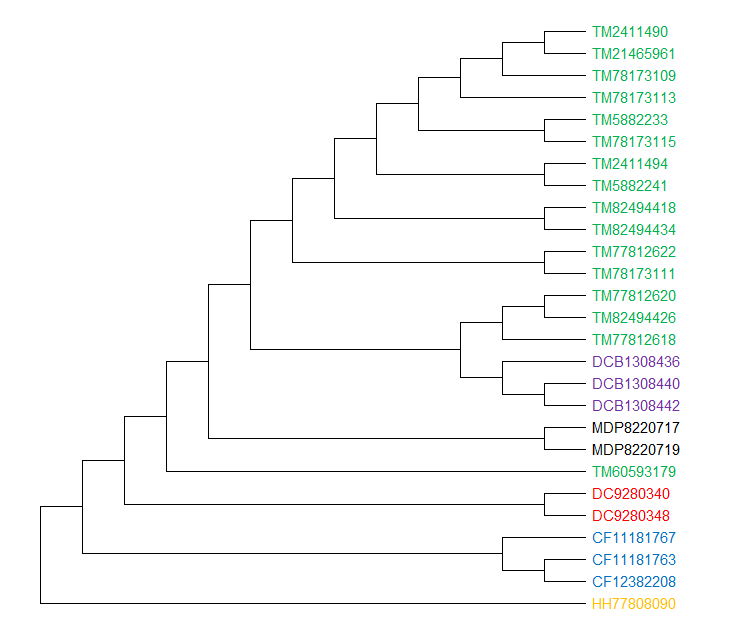


Fig. S 23 27 Antifreeze proteins (AFPs) sequences constructed by ClsutalW (NJ)


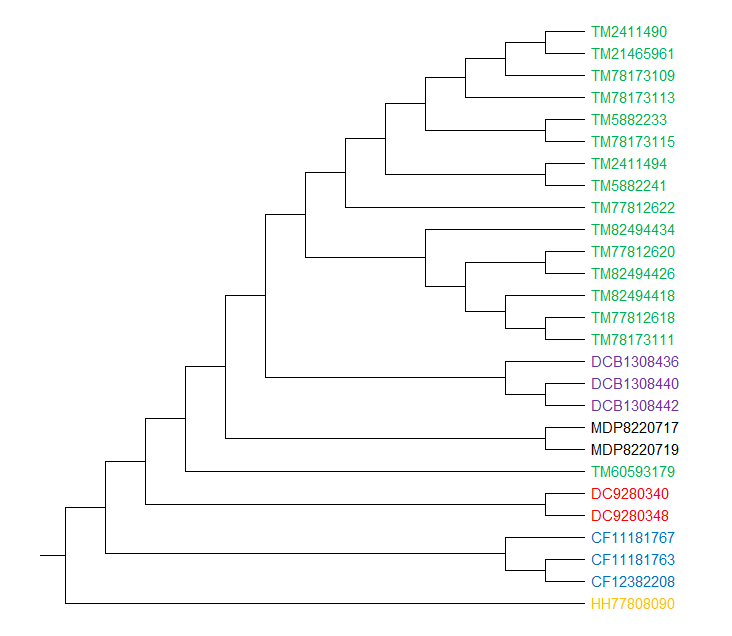


Fig. S 24 27 Antifreeze proteins (AFPs) sequences constructed by ClsutalW (UPGMA)


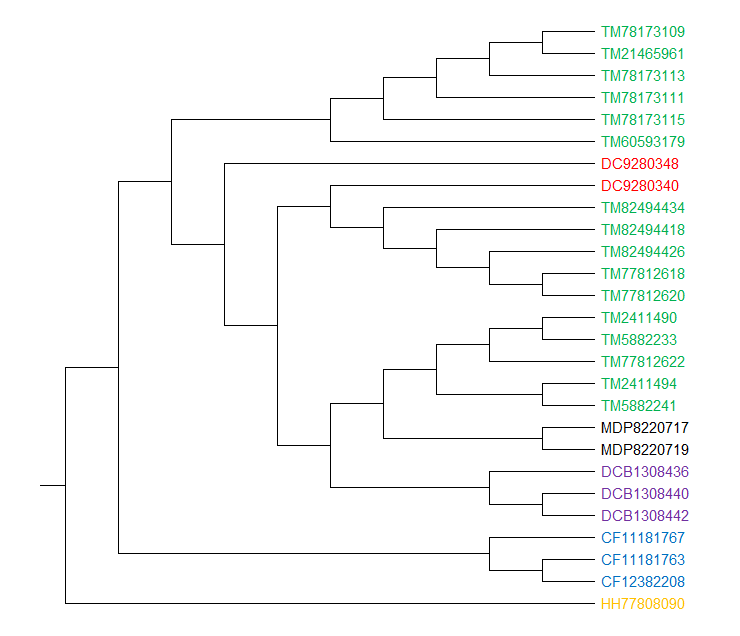


Fig. S 25 27 Antifreeze proteins (AFPs) sequences constructed by PCV

## 9 Betaglobin protein sequences


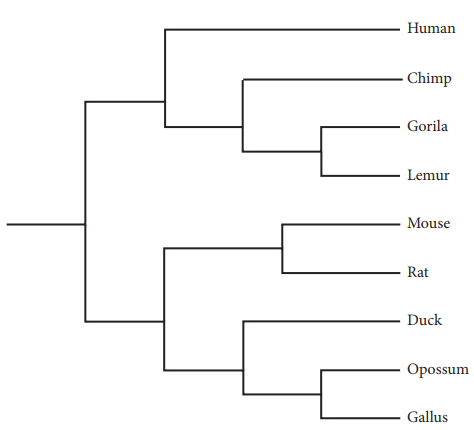


Fig. S 26 The phylogenetic tree of 9 betaglobin proteins constructed by intensity method [4]


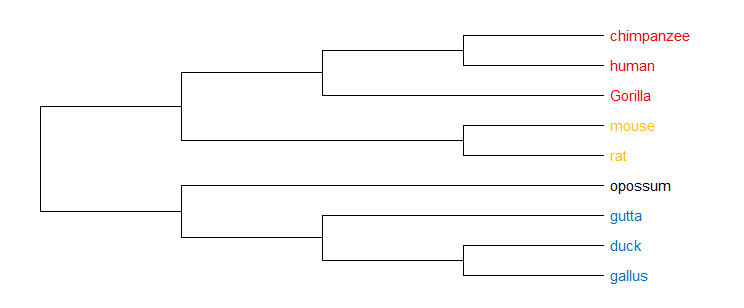


Fig. S 27 The phylogenetic tree of 9 betaglobin proteins constructed by ClustalW (NJ)


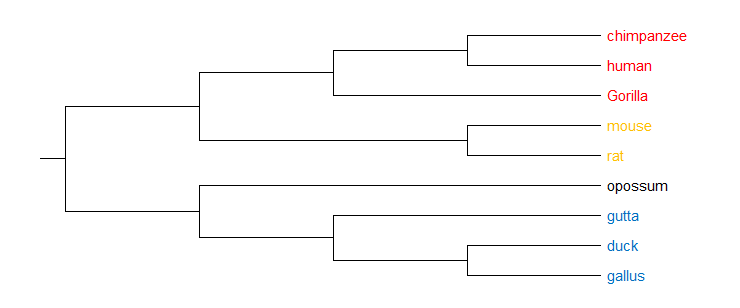


Fig. S 28 The phylogenetic tree of 9 betaglobin proteins constructed by ClustalW (UPGMA)


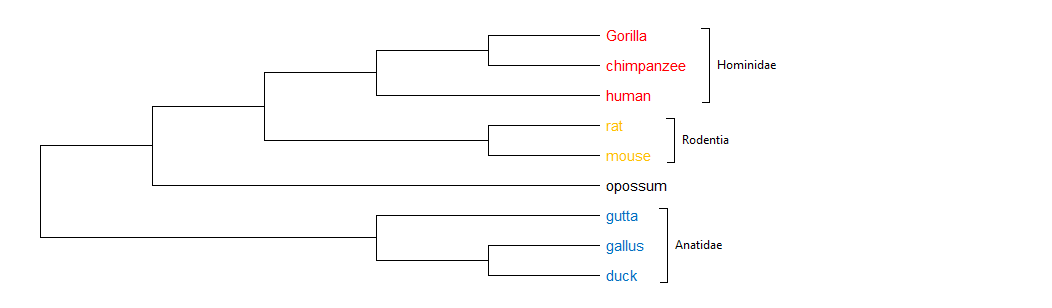


Fig. S 29 The phylogenetic tree of 9 betaglobin proteins constructed by PCV (NJ)

## 50 Betaglobin protein sequences


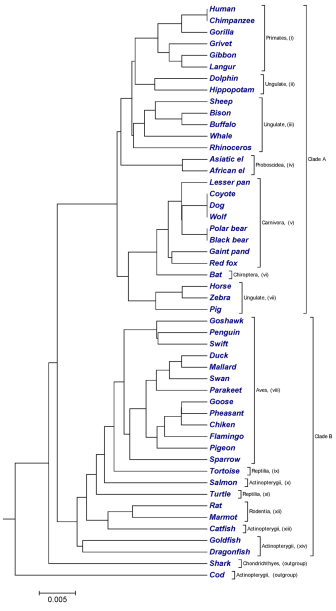


Fig. S 30 The phylogenetic tree of 50 betaglobin proteins constructed by fuzzy integral based method [3]


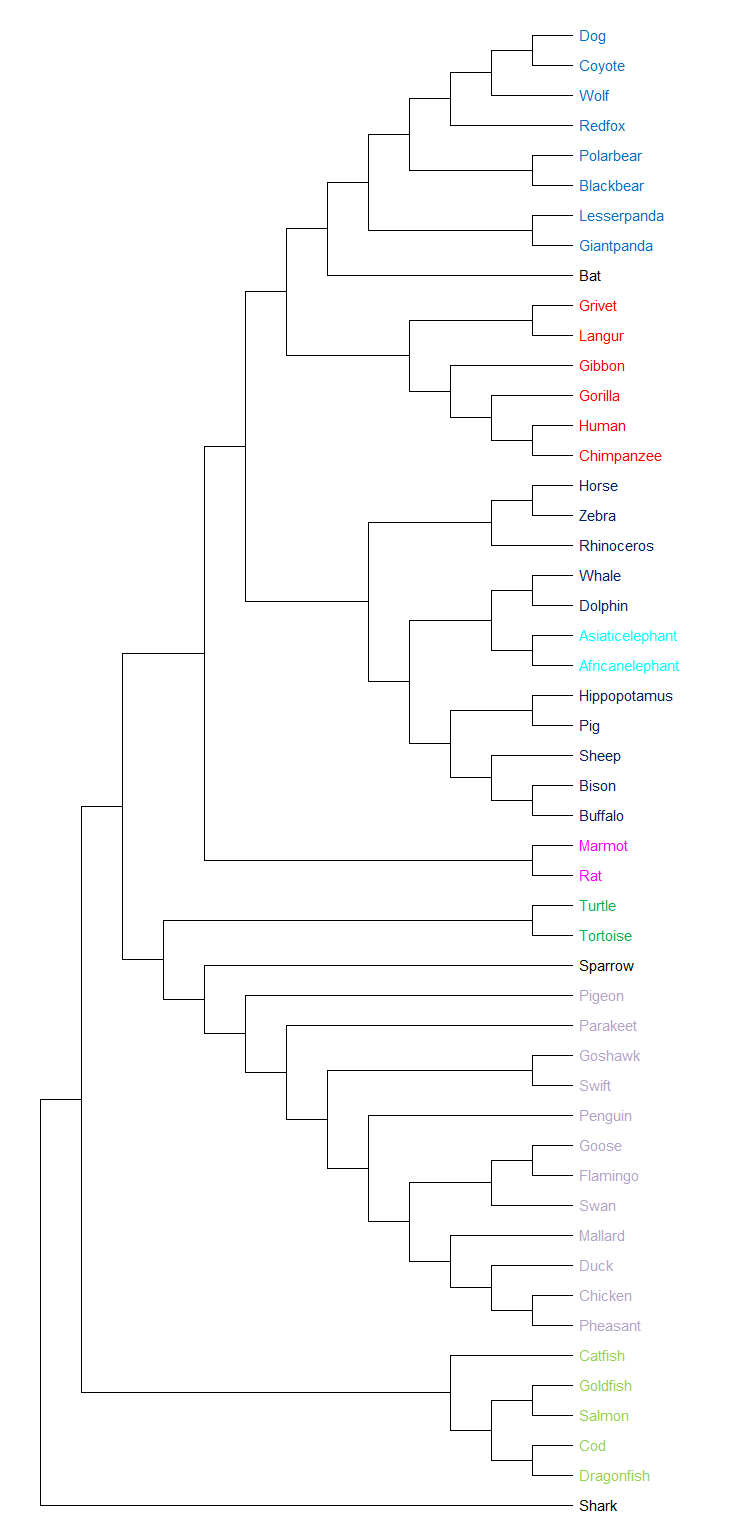


Fig. S 31 The phylogenetic tree of 50 betaglobin proteins constructed by ClustalW (NJ)


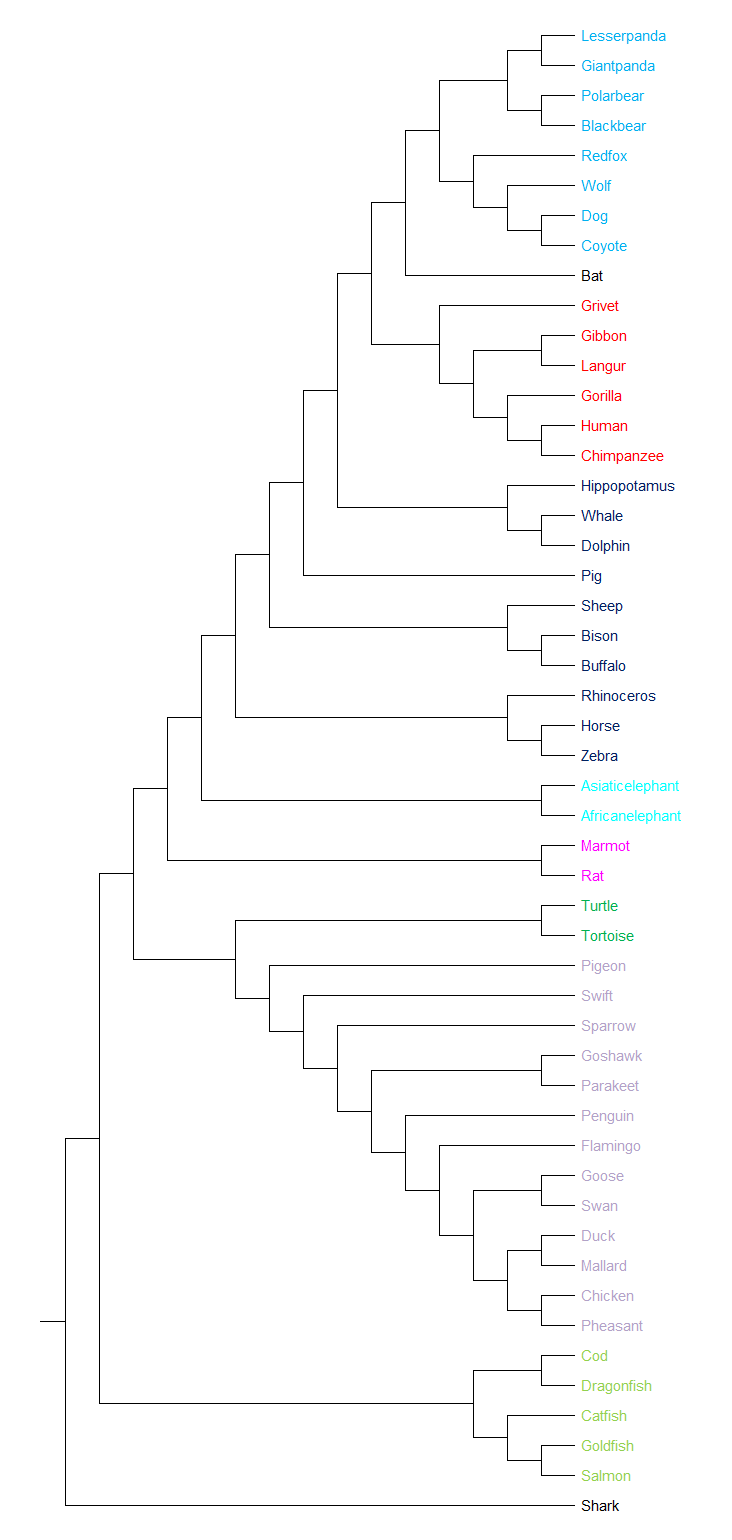


Fig. S 32 The phylogenetic tree of 50 betaglobin proteins constructed by ClustalW (UPGMA)


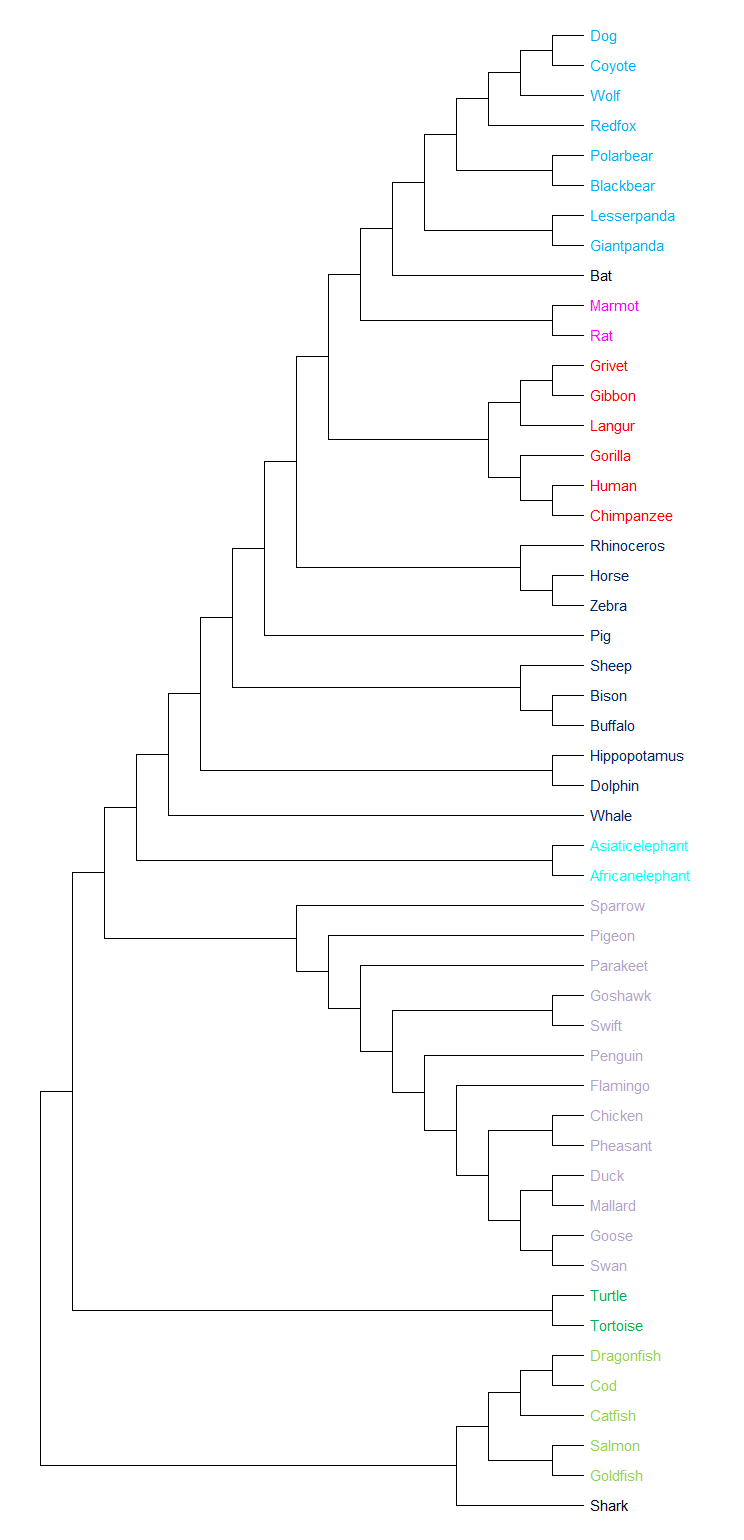


Fig. S 33 The phylogenetic tree of 50 betaglobin proteins constructed by PCV (NJ)

## 88 Betaglobin protein sequences


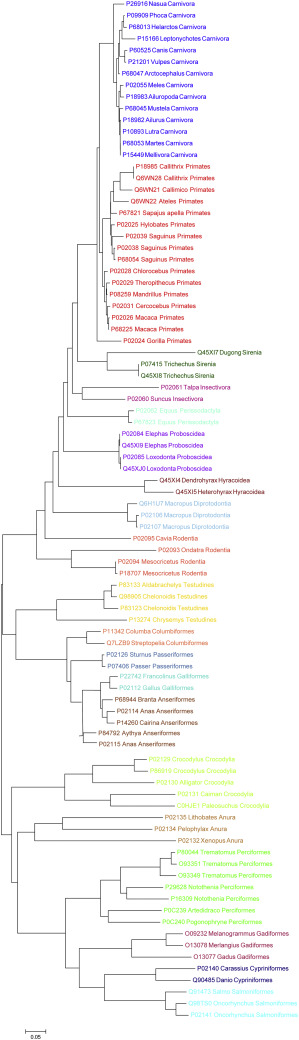


Fig. S 34 The phylogenetic tree of 88 betaglobin proteins constructed by Natural vector based method [7]


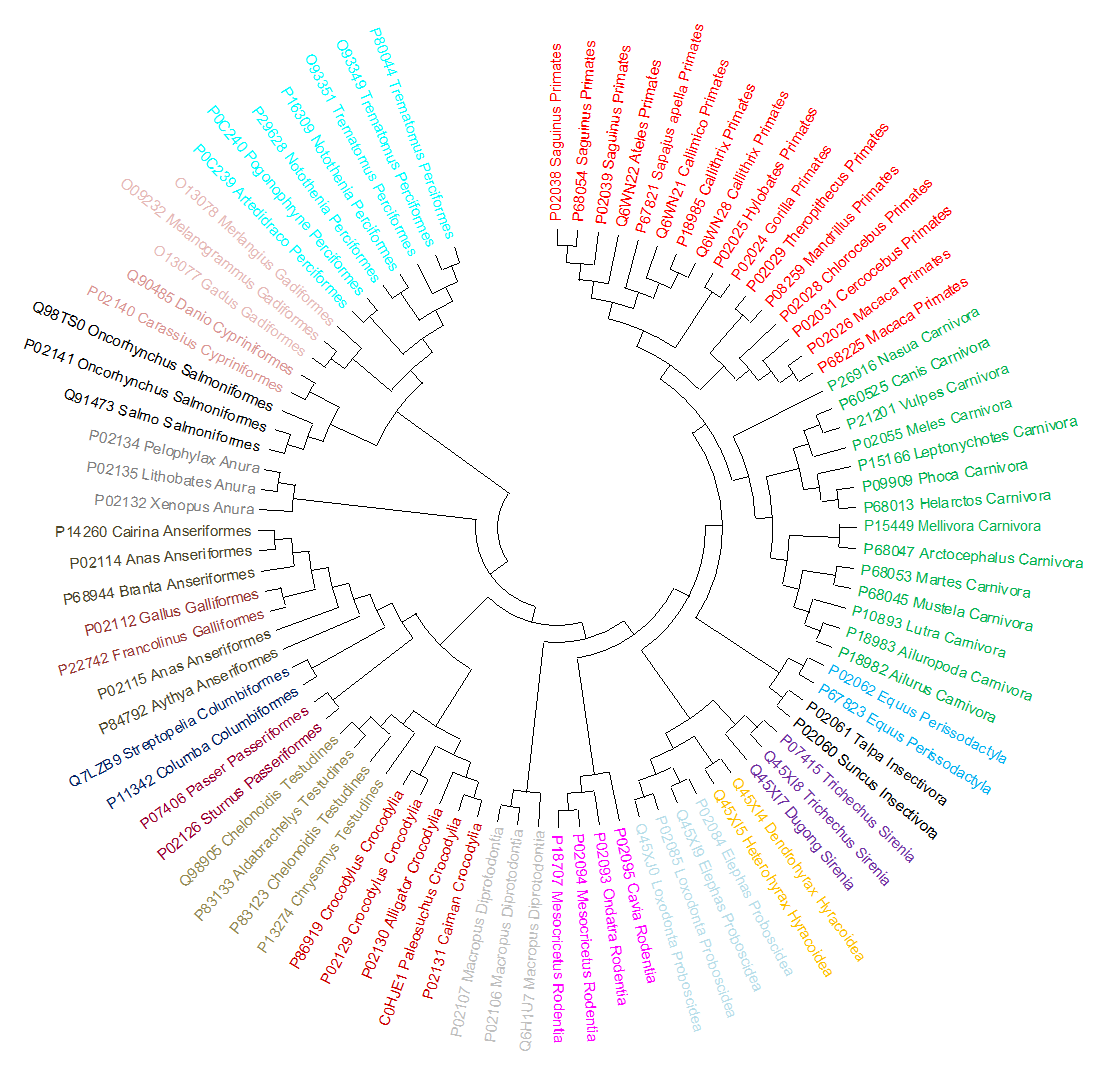


Fig. S 35 The phylogenetic tree of 88 betaglobin proteins constructed by ClustalW (NJ)


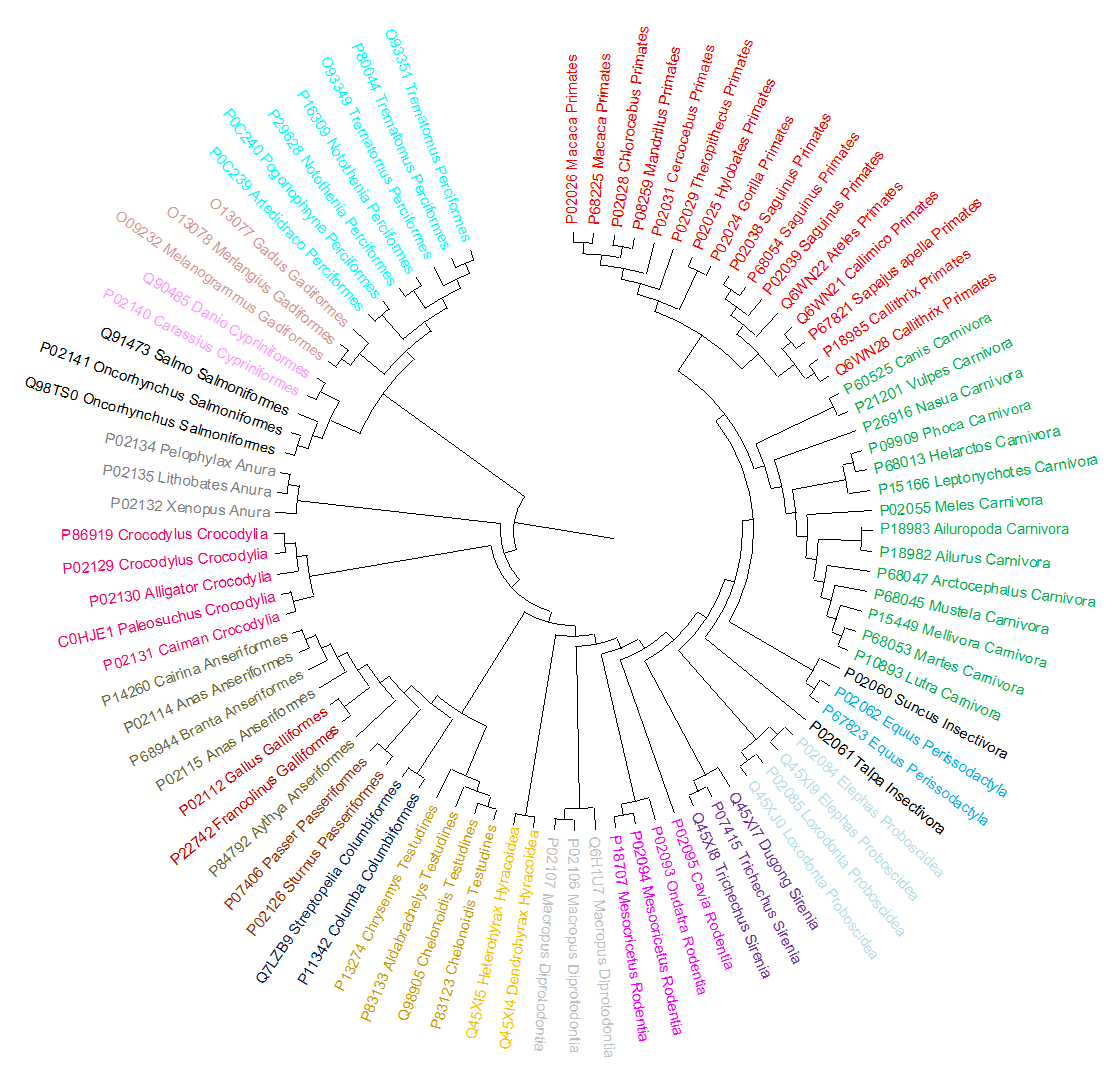


Fig. S 36 The phylogenetic tree of 88 betaglobin proteins constructed by ClustalW (UPGMA)


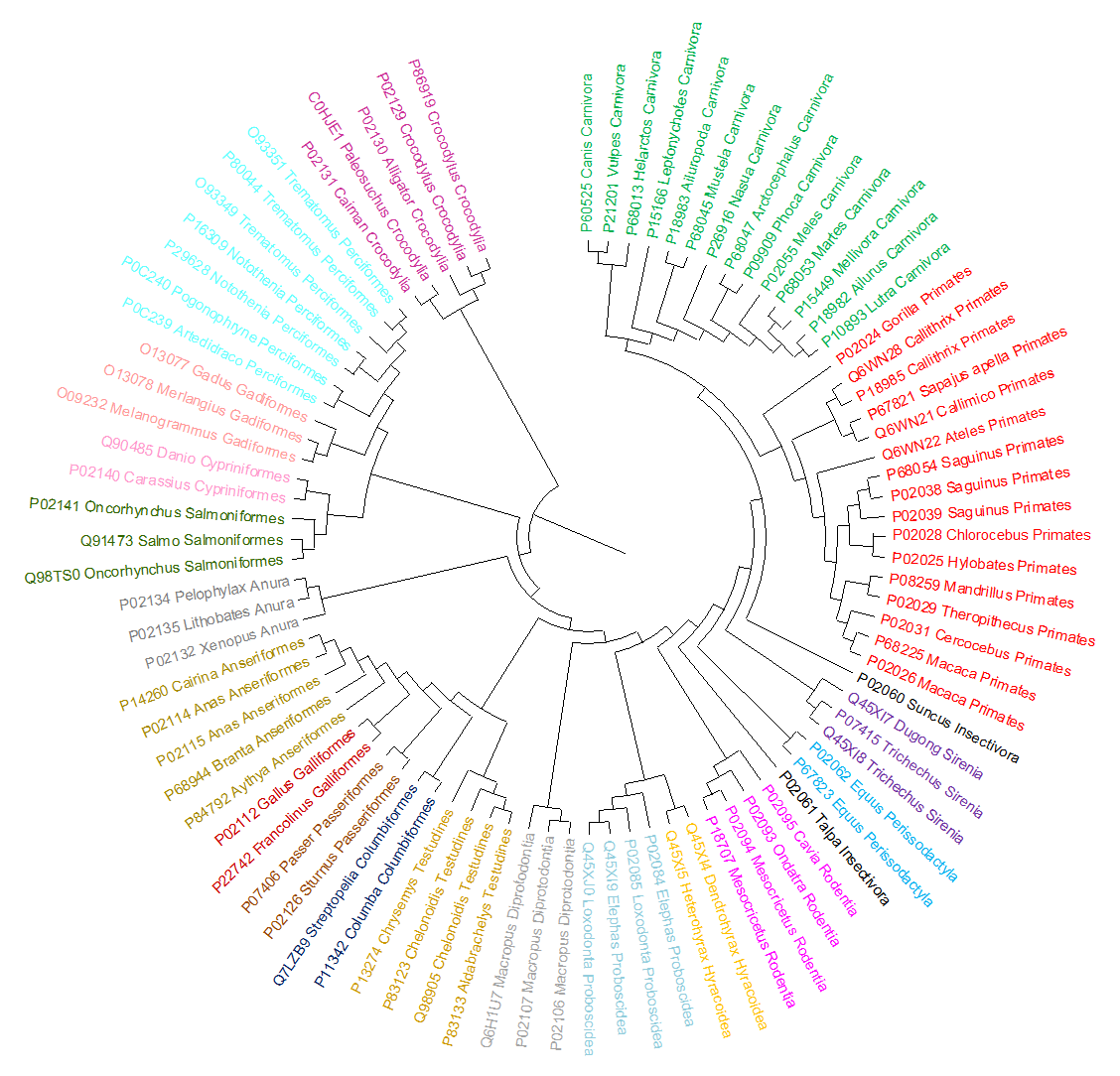


Fig. S 37 The phylogenetic tree of 88 betaglobin proteins constructed by PCV (UPGMA)

## 20 Xylanase protein sequences


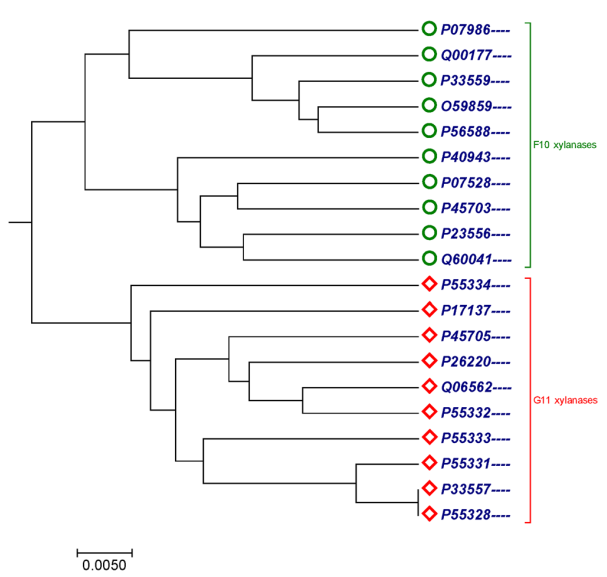


Fig. S 38 The phylogenetic tree of 20 Xylanase proteins constructed by fuzzy integral based method [3]


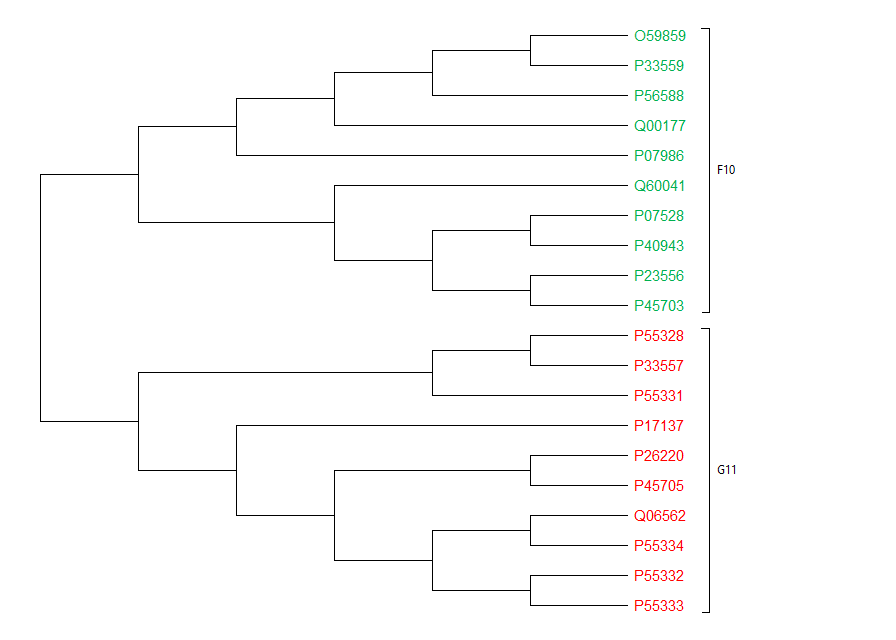


Fig. S 39 The phylogenetic tree of 20 Xylanase proteins constructed by ClustalW (NJ)


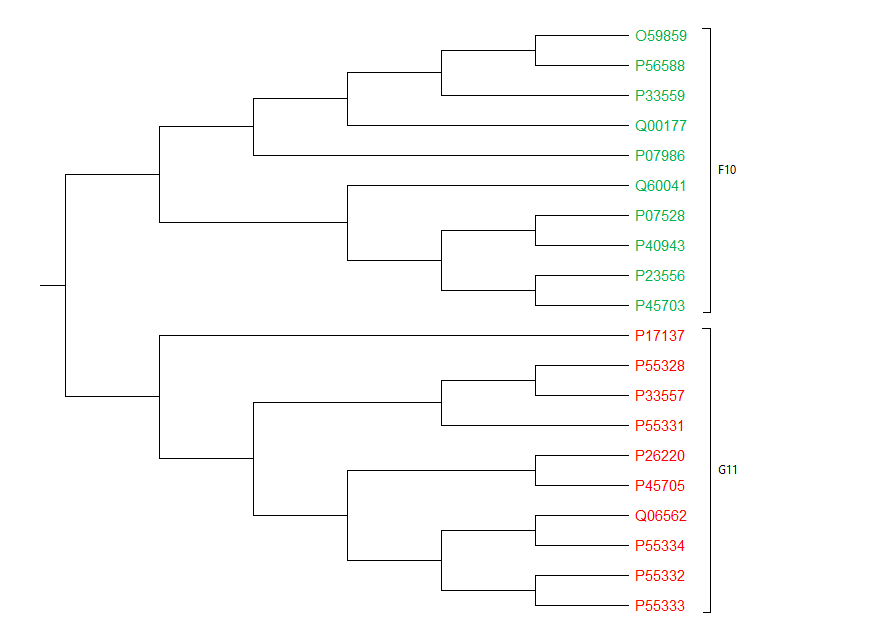


Fig. S 40 The phylogenetic tree of 20 Xylanase proteins constructed by ClsutalW (UPGMA)


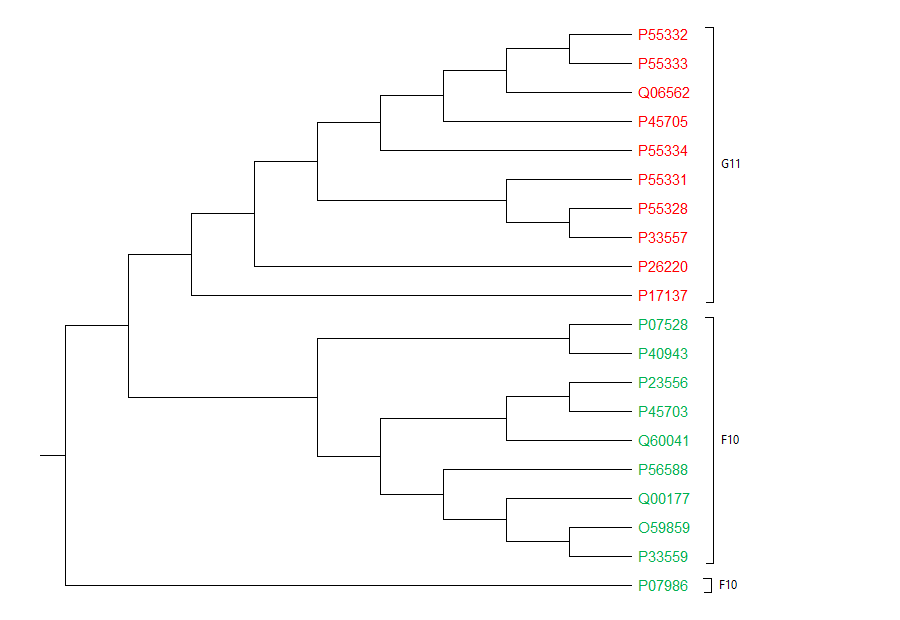


Fig. S 41 The phylogenetic tree of 20 Xylanase proteins constructed by PCV (UPGMA)

## 113 Human rhinoviruses (HRV) and 3 HEV-C protein sequences


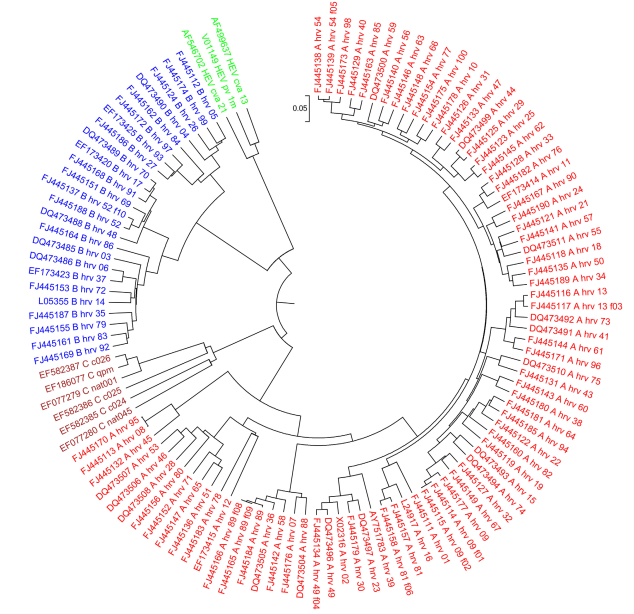


Fig. S 42 The phylogenetic tree of 113 Human rhinoviruses (HRV) and 3 HEV-C proteins constructed by Natural vector based method [7]


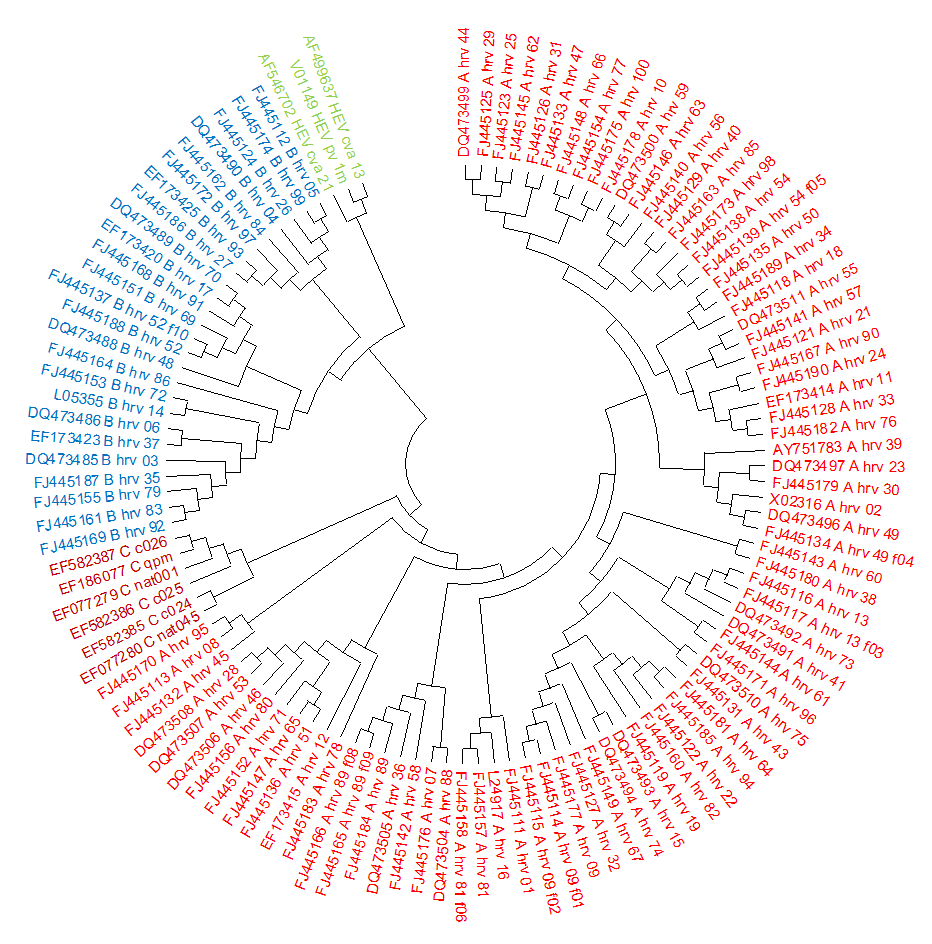


Fig. S 43 The phylogenetic tree of 113 Human rhinoviruses (HRV) and 3 HEV-C proteins constructed by ClustalW (NJ)


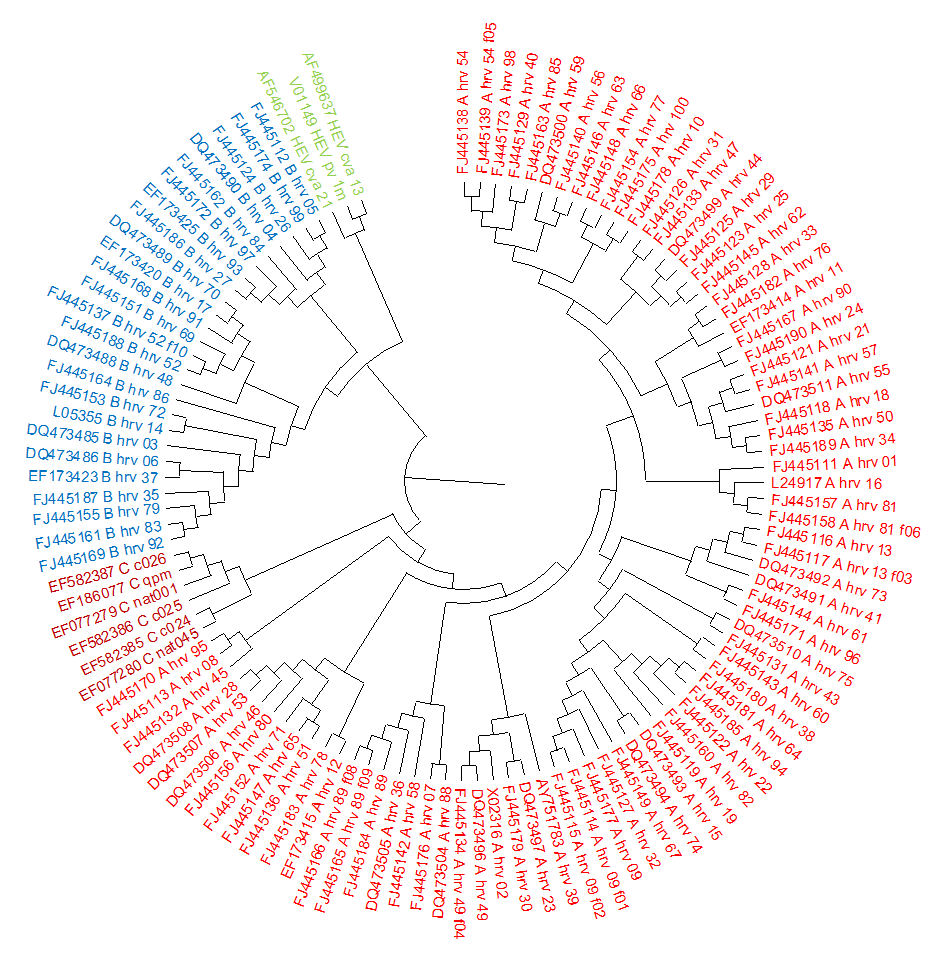


Fig. S 44 The phylogenetic tree of 113 Human rhinoviruses (HRV) and 3 HEV-C proteins constructed by ClustalW (UPGMA)


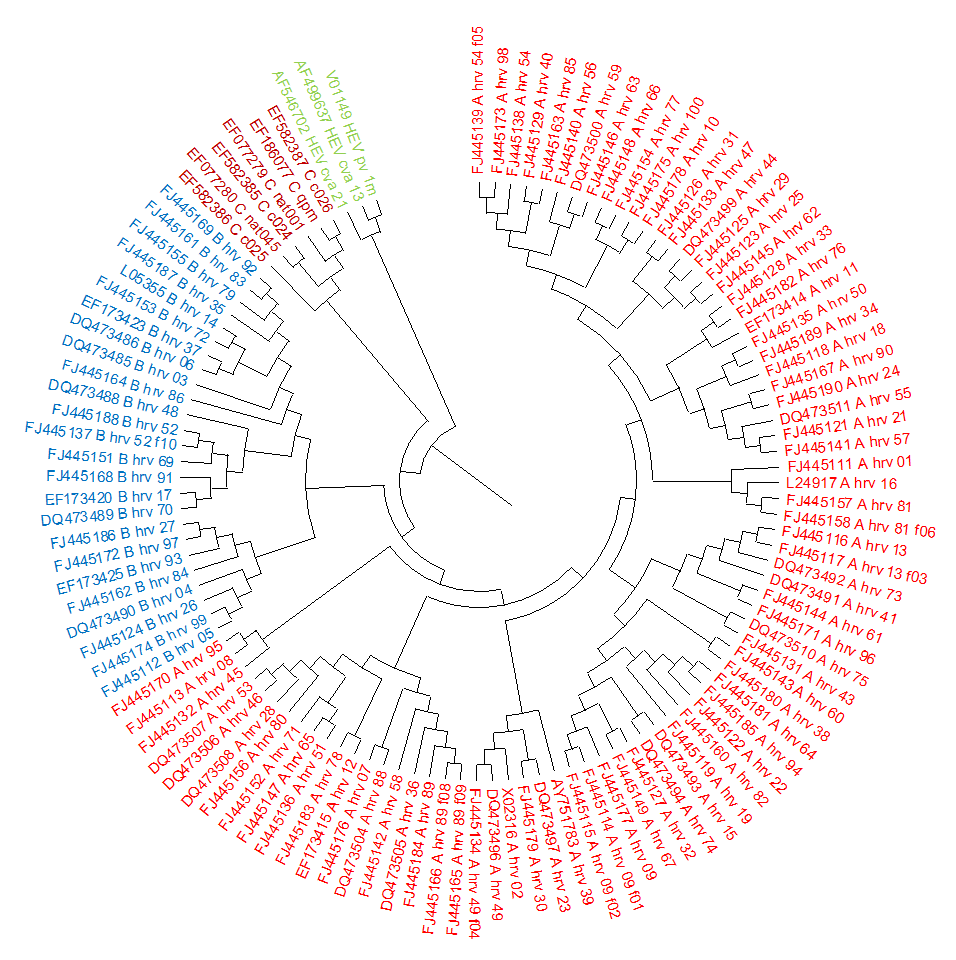


Fig. S 45 The phylogenetic tree of 113 Human rhinoviruses (HRV) and 3 HEV-C proteins constructed by PCV (UPGMA)

## 1163 Influenza A viruses protein sequences


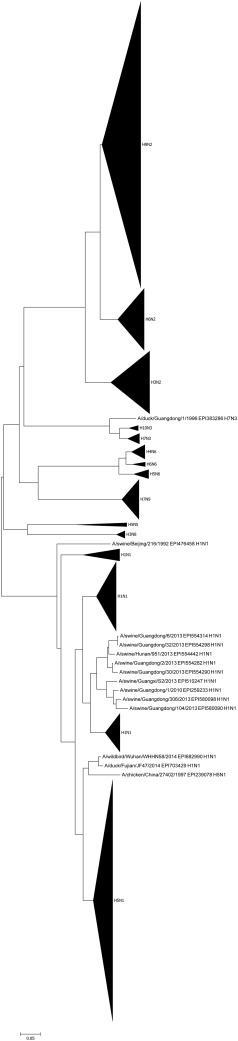


Fig. S 46 The phylogenetic tree of 1163 Influenza A viruses proteins constructed by Natural vector based method [7]


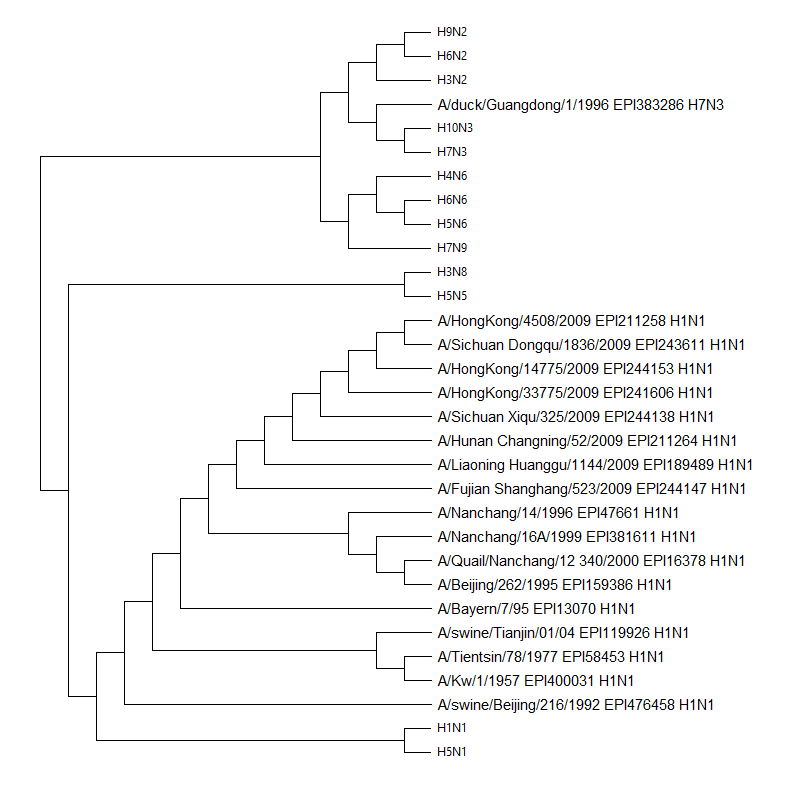


Fig. S 47 The phylogenetic tree of 1163 Influenza A viruses proteins constructed by ClustalW (NJ)


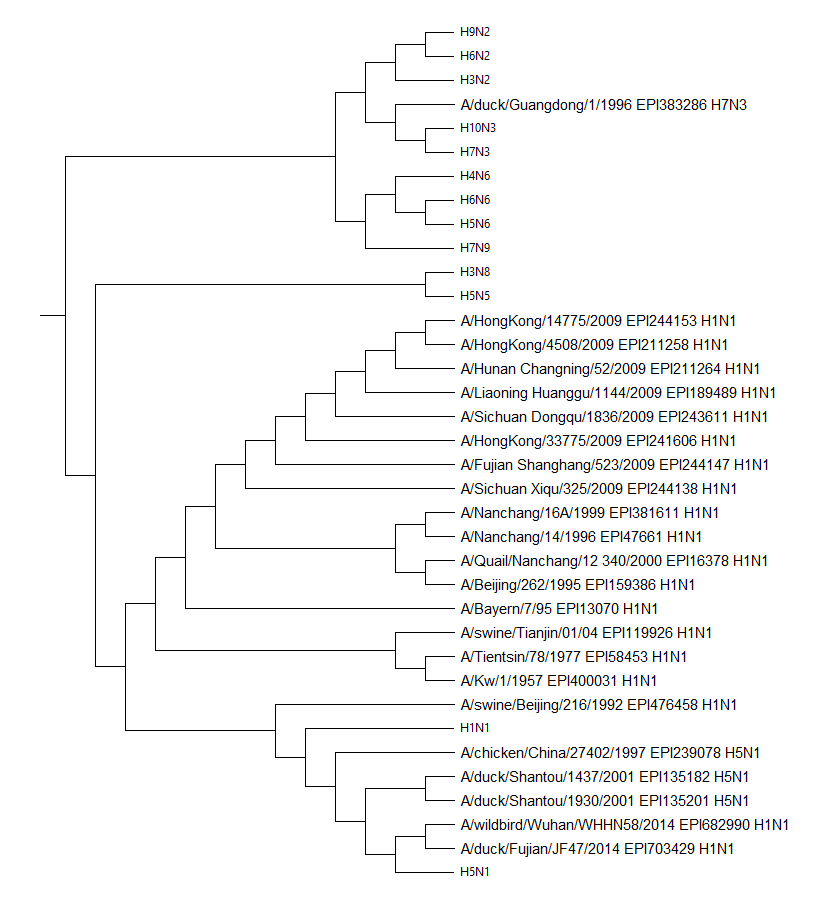


Fig. S 48 The phylogenetic tree of 1163 Influenza A viruses proteins constructed by ClustalW (UPGMA)


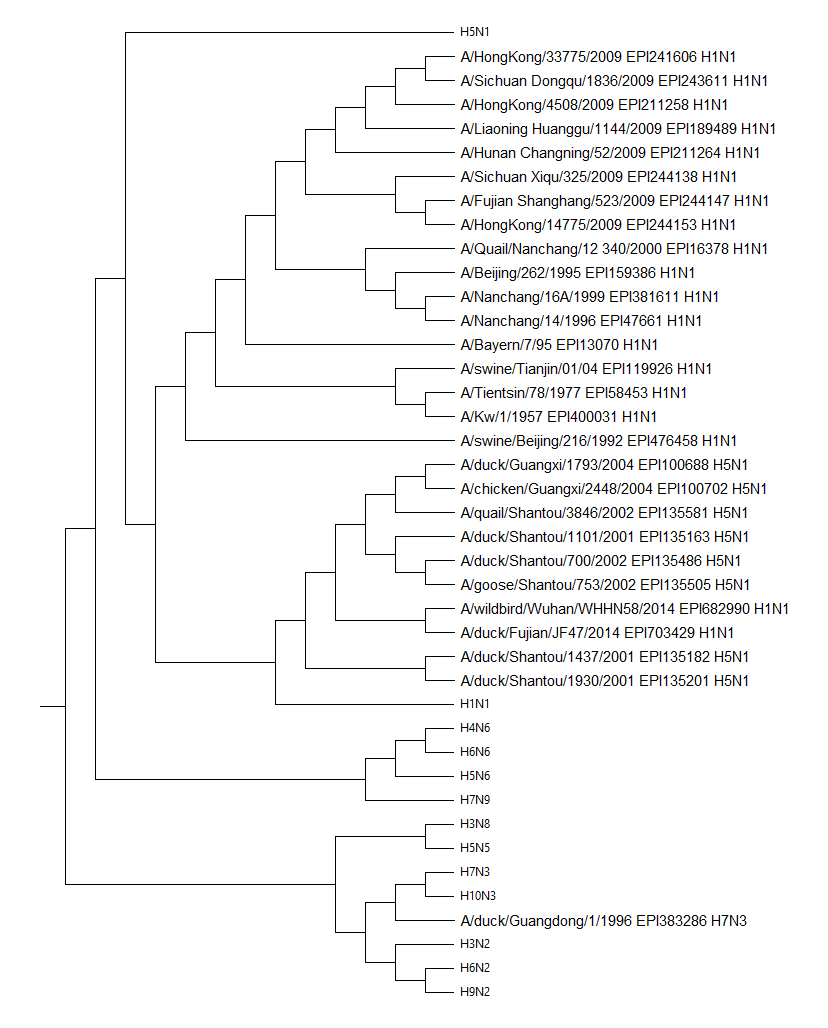


Fig. S 49 The phylogenetic tree of 1163 Influenza A viruses proteins constructed by PCV (UPGMA)

# References

[1] S. Kawashima, P. Pokarowski, M. Pokarowska, A. Kolinski, T. Katayama, and M. Kanehisa, “AAindex: amino acid index database, progress report 2008,” *Nucleic Acids Res.*, vol. 36, no. Database, pp. D202–D205, Dec. 2007.

[2] “scipy.cluster.hierarchy.linkage tutorial.” [Online]. Available: https://docs.scipy.org/doc/scipy/reference/generated/scipy.cluster.hierarchy.linkage.html.

[3] A. K. Saw, B. C. Tripathy, and S. Nandi, “Alignment-free similarity analysis for protein sequences based on fuzzy integral,” *Sci. Rep.*, vol. 9, no. 1, p. 2775, Dec. 2019.

[4] M. M. Abo-Elkhier, M. A. Abd Elwahaab, and M. I. Abo El Maaty, “Measuring Similarity among Protein Sequences Using a New Descriptor,” *Biomed Res. Int.*, vol. 2019, pp. 1–10, Nov. 2019.

[5] P. Sonego, A. Kocsor, and S. Pongor, “ROC analysis: applications to the classification of biological sequences and 3D structures,” *Brief. Bioinform.*, vol. 9, no. 3, pp. 198–209, Jan. 2008.

[6] L. Yu, Y. Zhang, I. Gutman, Y. Shi, and M. Dehmer, “Protein Sequence Comparison Based on Physicochemical Properties and the Position-Feature Energy Matrix,” *Sci. Rep.*, vol. 7, no. 1, p. 46237, May 2017.

[7] Y. Zhang, J. Wen, and S. S. T. Yau, “Phylogenetic analysis of protein sequences based on a novel k-mer natural vector method,” *Genomics*, vol. 111, no. 6, pp. 1298–1305, Dec. 2019.
